# The efficacy, safety, and cost-effectiveness of TMJ balancing therapy for temporomandibular joint disorder: study protocol for a randomized controlled trial

**DOI:** 10.1186/s12906-026-05303-8

**Published:** 2026-03-11

**Authors:** Miso S. Park, Junyoung Hur, Jungeun Choi, Bo-Hyoung Jang, Young-Jun Lee, HoRyong Yoo

**Affiliations:** 1 Department of Cardiology and Neurology of Korean Medicine, College of Korean Medicine, Daejeon University, 75 Daedeok-daero 176beon-gil, Seo-gu, Daejeon, 35235 Republic of Korea; 2 Department of Preventive Medicine, College of Korean Medicine, Kyung Hee University, 26 Kyungheedae-ro, Dongdaemun-gu, Seoul, 02447 Republic of Korea; 3 Lee Young Jun Clinic of Korean Medicine, Yongam Bldg 5th fl., 388 Bongjeong-ro, Seobuk-gu, Cheonan, Chungcheongnam-do 31103 Republic of Korea

**Keywords:** Temporomandibular joint disorder, TMJ balancing therapy, TENS, Randomized controlled trial, Protocol, Korean medicine

## Abstract

**Background:**

Temporomandibular joint disorders (TMD) are common musculoskeletal conditions affecting the masticatory system, leading to pain and functional limitations. Despite various conventional treatments, parts of the 2021 Korean Medicine Standard Clinical Practice Guidelines for TMD remain unaddressed due to insufficient supporting evidence for certain modalities like intraoral balancing appliances. This study aims to investigate a novel approach, temporomandibular joint balancing therapy (TBT), as a potential effective treatment.

**Methods:**

This will be a single-center, randomized, assessor-blinded, parallel-group clinical trial. A total of 30 eligible participants aged 19 to 70 years with chronic TMD (VAS ≥ 40 mm for ≥ 3 months) will be randomized 1:1 to either the experimental group (TBT) or the control group (Transcutaneous Electrical Nerve Stimulation, TENS). Both interventions will be administered twice a week for 6 weeks (12 sessions). The primary outcome is the mean change in temporomandibular joint pain measured by the Visual Analog Scale (VAS, 100 mm) at 6 weeks compared to baseline. Secondary outcomes include worst TMD pain VAS, jaw range of motion (pain-free opening, maximum unassisted opening), Jaw Functional Limitation Scale (JFLS-8), DC/TMD Graded Chronic Pain Scale (GCPS 2.0), Treatment Expectancy Scale, Patient Global Impression of Change (PGIC), quality-adjusted life years (QALYs), EQ-5D, EQ-VAS, medical costs, lost productivity costs, and incremental cost-effectiveness ratio (ICER). Data will be collected at baseline, 6 weeks, and 10 weeks (for follow-up). Statistical analysis will compare outcomes between groups using appropriate methods, primarily intention-to-treat analysis.

**Discussion:**

This study protocol details a randomized controlled trial designed to provide robust evidence on the efficacy, safety, and economic feasibility of TBT for TMD. The findings are expected to contribute to the evidence base for TMD management, potentially informing future clinical practice guidelines, particularly concerning the use of intraoral balancing appliances in Korean medicine.

**Trial registration:**

This trial was retrospectively registered with the CRIS (http://cris.nih.go.kr). Registry: https://cris.nih.go.kr/cris/search/detailSearch.do?seq=29064, trial registration number: KCT0010382, date of registration: 3 April 2025.

**Supplementary Information:**

The online version contains supplementary material available at 10.1186/s12906-026-05303-8.

## Background

Temporomandibular joint disorder (TMD) encompasses a group of musculoskeletal and neuromuscular conditions affecting the temporomandibular joint (TMJ), masticatory muscles, and related structures [1]. TMD is commonly characterized by pain in the jaw, face, and head, as well as limited mandibular movement and joint sounds such as clicking and popping, all of which can significantly impair quality of life and daily functioning [2]. Its multifactorial etiology includes biomechanical stressors such as occlusal overloading and oral parafunctional habits including bruxism and clenching, hormonal influences like elevated estrogen levels, and psychological factors such as stress and anxiety. Understanding these contributing elements is necessary for accurate diagnosis and effective, individualized treatment planning [3].

The prevalence of TMD varies based on the target population, diagnostic criteria, and research methodology. Large-scale studies in the United States suggest that approximately 5% to 12% of the population experiences symptoms of TMD [4]. In Korea, recent nationwide cohort data from the National Health Insurance Service revealed that the age-standardized prevalence of clinically diagnosed TMD increased steadily from 604 per 100,000 persons in 2012 to 869 per 100,000 in 2020. The prevalence was particularly high among young adults, with the highest rates observed in individuals in their 20s (1,809 per 100,000), followed by teenagers and those in their 30s. Female patients consistently showed a 1.5-fold higher prevalence than males, aligning with global trends. Among the various TMD subtypes, internal derangement of the temporomandibular joint and unspecified joint pain were the most frequently recorded diagnoses [5].

Data from the Health Insurance Review & Assessment Service (HIRA) healthcare big data open system (accessed May 3, 2025) also reveals a consistent increase in patients diagnosed with TMD (Korean Standard Classification of Diseases K076) in South Korea from 2019 to 2023. Over the past five years, the number of patients with TMD treated at medical institutions—including hospitals, clinics, Korean medicine facilities, and dental centers—increased by approximately 25.78%, exceeding 560,000 individuals in 2023. Concurrently, the total medical expenditure associated with TMD increased significantly, rising from approximately 44.9 billion Korean Won (KRW) in 2019 to about 70.3 billion KRW in 2023, representing a 56.57% increase over the five years.

Conventional treatments remain the first-line approach for managing TMD, with interventions including physical therapy, manual therapy, exercise therapy, and reversible occlusal splints being most commonly applied [6]. Recent international clinical practice guidelines, such as those proposed by the International Network for Orofacial Pain and Related Disorder Methodology (INfORM) under the International Association for Dental, Oral, and Craniofacial Research (IADR) emphasize a patient-centered approach that prioritizes self-management strategies, education, and cognitive behavioral therapy (CBT), with surgical options reserved strictly for select, refractory cases [7]. Among non-invasive modalities, techniques such as joint mobilization, trigger point therapy, and posture-corrective exercises have demonstrated clinical utility [8]. Additionally, oral parafunctional habits, which can exacerbate pain and tension in muscles [9], are often addressed through behavioral modification and psychosocial support [10]. Meanwhile, pharmacological treatments, including NSAIDs, muscle relaxants, and antidepressants, are commonly used despite concerns about adverse effects and the lack of standardized regimens [11].

Emerging adjunctive therapies, such as transcutaneous electrical nerve stimulation (TENS) [12], low-level laser therapy (LLLT) [13], and therapeutic ultrasound [14], have garnered attention for their potential to alleviate pain and improve mandibular function, though long-term evidence remains limited. LLLT, in particular, has shown promising short-term results for muscle-related TMD pain [13], outperforming TENS and therapeutic ultrasound in meta-analyses [15]. Injection therapies, including botulinum toxin (BTX), corticosteroids, and hyaluronic acid, are selectively employed when conservative measures fail [16]. BTX injections into the masticatory muscles are increasingly utilized, offering temporary relief for myofascial pain and parafunctional activity [17], though concerns about functional impairment and the need for repeated administration persist [18]. Despite the wide range of available therapies, clinical outcomes often vary based on individual patient characteristics. Therefore, multidisciplinary, personalized treatment planning and further high-quality studies are needed to guide evidence-based decision-making. In addition, it is noteworthy that despite the short-term symptom relief achieved by most conservative treatments, their long-term efficacy remains uncertain in many cases [19].

This study aims to evaluate the efficacy, safety, and cost-effectiveness of TMJ balancing therapy (TBT) for TMD in comparison to TENS, a widely used conservative treatment modality. TBT is a structural medicine approach designed to correct misalignments of the entire spine and body primarily by restoring the balance of the TMJ and the axis (C2 cervical vertebra). Unlike conventional treatments that often focus on symptom management or localized interventions, TBT offers a distinctive value proposition by addressing the underlying structural and neurological imbalances contributing to TMD. TBT regards the TMJ as a central joint that influences the structural and functional balance of the brain, cervical muscles, nervous system, and the spinal column. By restoring the optimal multidimensional balance position of the TMJ using an intraoral balancing appliance, TBT aims to address both structural and neurological imbalances that are closely linked to the misalignment of the TMJ. Once the TMJ is realigned, patients perform stretching and postural exercises to reinforce proper alignment, particularly the C2 vertebra, thereby promoting spinal stability and nervous system regulation [20].

The rationale for this approach is supported by radiographic studies demonstrating a close anatomical and functional link between the TMJ and the upper cervical spine (particularly C2). For example, Nagashima et al. reported a correlation between the occiput-C2 (O-C2) angle and the mandibular gonion-C2 distance, indicating that a decreased O-C2 angle can lead to posterior mandibular displacement and pharyngeal narrowing [21]. Similarly, Thomas et al. found that a significant majority (83%) of their study subjects exhibited posteriorly pitched crania, with 75% showing posteriorly compressed TMJs and 80% displaying rightward rotation of the atlanto-occipital joint [22]. These findings collectively suggest the dynamic structural relationship between the TMJ and C2, emphasizing the need for a comprehensive approach to TMJ disorder that considers the connections between the TMJ, cervical spine, skull, and overall body balance, especially when structural issues have become chronic [20].

In light of this evidence, therapies such as TBT that address the dynamic interrelationship between the jaw, cervical spine, and postural alignment may offer clinical benefits for patients with chronic or recurrent TMD. Formerly referred to as Functional Cerebrospinal Therapy (FCST), TBT has been reported to yield long-term symptom improvement in challenging conditions such as chronic recurrent TMJ dislocation and other treatment-resistant disorders [23]. Given that TMD is often associated with structural imbalances resulting from maladaptive oral and postural habits, conventional treatment modalities may fail to fully correct these underlying biomechanical dysfunctions. Therefore, when TBT is applied to correct these structural issues, its therapeutic effects are expected to be more sustainable, particularly in long-term follow-up.

## Methods/design

### Study design

**Fig. 1:**
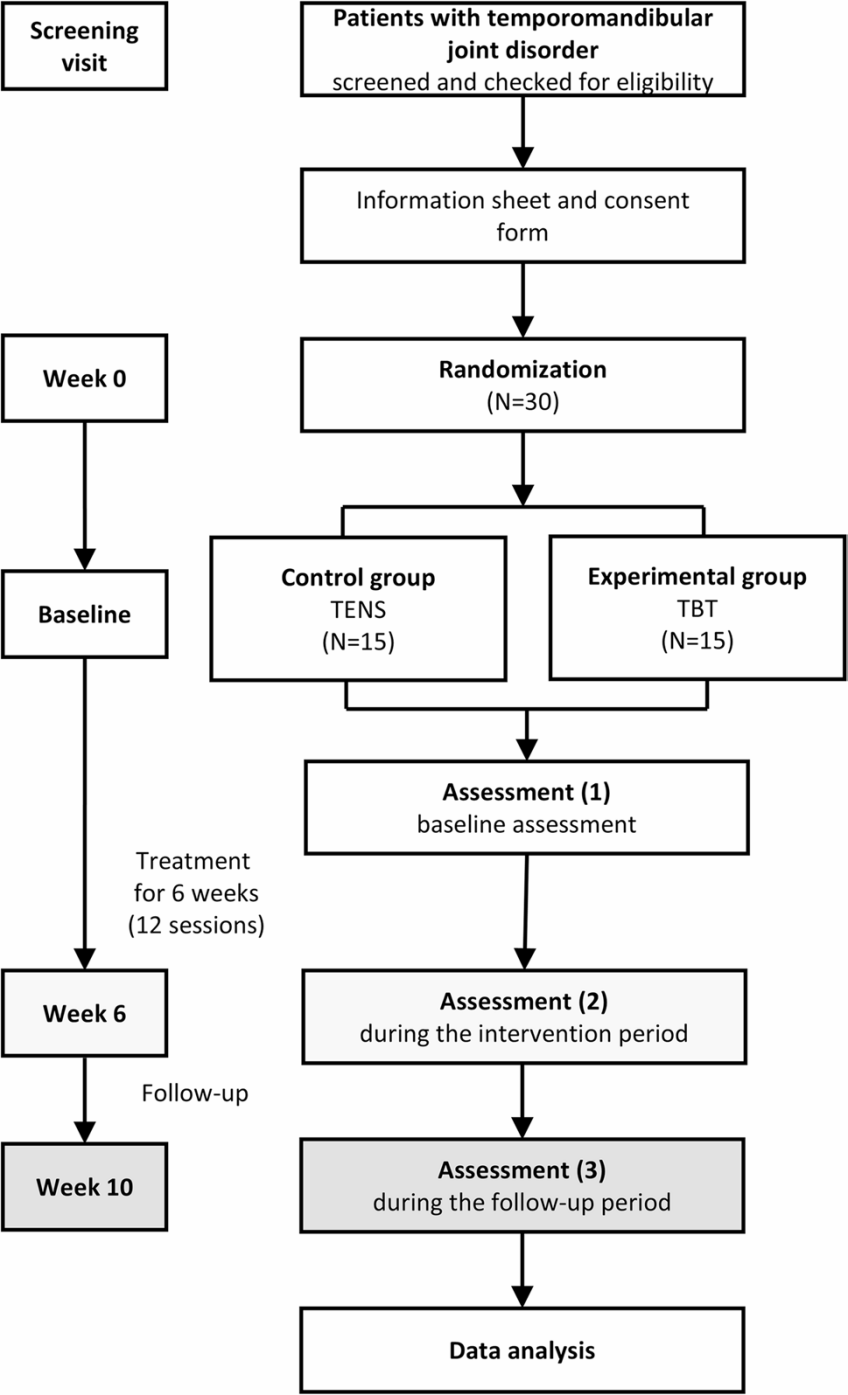
Flowchart of the study plan. TBT; temporomandibular joint-balancing-therapy, TENS; transcutaneous electrical nerve stimulation

**Fig. 2:**
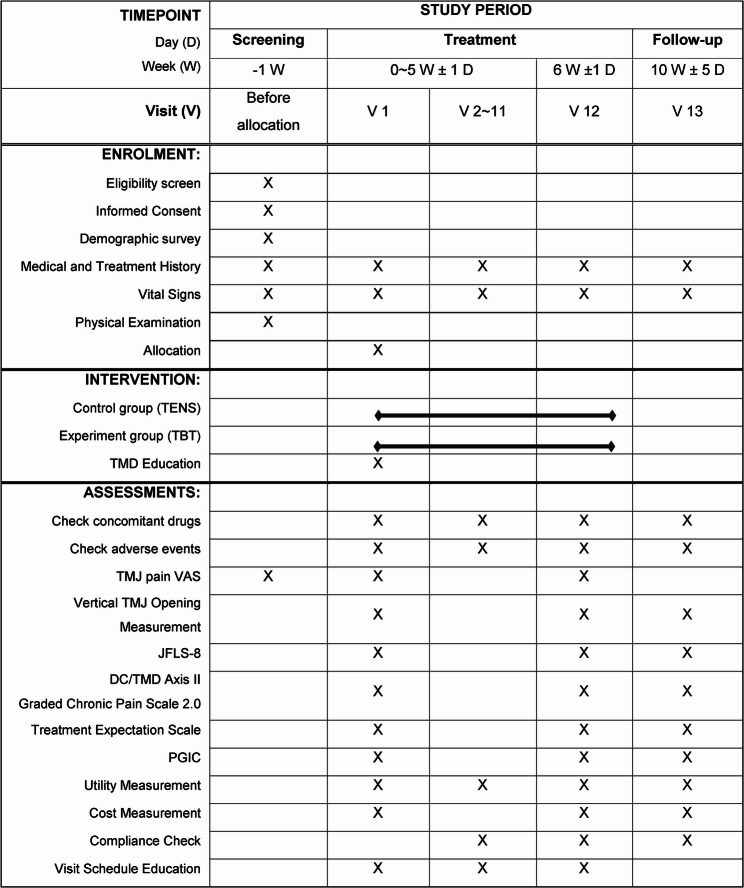
Schedule of enrollment, interventions, and outcome measurements

This study will be a single-center, randomized, assessor-blinded, active-controlled, parallel-group clinical trial designed to evaluate the efficacy, safety, and cost-effectiveness of TBT for TMD. A total of 30 participants will be recruited and randomly assigned in a 1:1 ratio to the TBT experimental group and TENS control group. The study consists of a screening visit, 12 treatment visits over 6 weeks (twice weekly), and a follow-up visit at week 10. The detailed study design is summarized in Fig. 1; Table 1. This protocol adheres to the Standard Protocol Items: Recommendations for Interventional Trials (SPIRIT) 2013 guidelines and checklist (Supplementary material 1) [24]. The flowchart of this study is shown in Fig. 1. The SPIRIT schedule of enrollment, interventions, and assessments is shown in Fig. 2.

**Table 1:** Eligibility criteria

**Inclusion Criteria**:
1. Men and women aged 19–70 years
2. Patients who experience intermittent pain persisting for more than three months in either or both temporomandibular joints (TMJ) according to the Diagnostic Criteria for Temporomandibular Disorders (DC/TMD)
3. Patients whose average pain intensity in the most painful TMJ (for bilateral pain, the more painful side was considered) over the past week is ≥ 40 mm on the Visual Analogue Scale (VAS)
4. Patients who present with localized pain in the TMJ or surrounding areas (temple, ear canal, or preauricular area) induced by jaw movement, function, or parafunction
5. Patients whose pain is confirmed by palpation of the temporalis muscle, masseter muscle, or the lateral pole of the mandibular condyle, corresponding to myalgia, myofascial pain, or arthralgia, or those with a headache located in the temporal region that is provoked or aggravated by jaw movement and is reproducible upon examination
**Exclusion Criteria**:
1. Patients with a history of surgery related to the TMJ
2. Patients with multiple pain disorders (e.g., rheumatoid arthritis) or neurological disorders (e.g., brain tumor, stroke, trigeminal neuralgia) that could interfere with treatment effects or the interpretation of results
3. Patients who are currently taking steroids, immunosuppressants, psychiatric medications, or other drugs that could affect study outcomes
4. Patients who have started or discontinued medications that could influence pain, such as non-steroidal anti-inflammatory drugs (NSAIDs), within the past month
5. Patients deemed by the investigator to be unsuitable for participation in the study (e.g., those with implanted electrical devices such as pacemakers)

### Participants

Participants will be recruited through advertisements in local hospitals and community centers. Eligibility will be assessed via a structured interview and physical examinations by a trained doctor of Korean Medicine at Daejeon Korean Medicine Hospital of Daejeon University, according to the eligibility criteria shown in Table 1. All participants must manually sign a written informed consent form (ICF) before any study procedures.

### Sample size calculation and statistical hypotheses

The sample size for this trial was determined based on statistical calculations and reference to a previous randomized controlled trial with a similar design [25]. A total of 30 participants (15 in the intervention group and 15 in the control group) will be recruited, considering a 20% dropout rate. This allows for 24 evaluable subjects (12 per group) for the primary analysis.

The hypothesis of this study is as follows:

$$\mathrm{H}_o\left(\text{null hypothesis}\right):\mu\mathrm{t}=\mu\mathrm{c}$$

$$\mathrm{H}_1\left(\text{alternative hypothesis}\right):\mu\mathrm{t}\neq\mu\mathrm{c}$$

µt denotes the mean change in Visual Analog Scale (VAS, mm) score from baseline to 6 weeks in the experimental group (TBT), and µc is the mean change in VAS (mm) score from baseline to 6 weeks in the control group (TENS). The experimental and control groups will be recruited in a one-to-one ratio.

The calculation assumed a two-sided significance level (α) of 0.05, a type II error (β) of 0.1 (power = 90%), and a 1:1 allocation ratio. A previous RCT with a similar design (10-week intervention, 12 sessions) reported a mean difference of − 2.53 in VAS between groups, with a pooled standard deviation (SD) of 0.96 (Rezaie et al., 2022). For conservative estimation, this study applied a smaller mean difference (–2) and larger SD (1.5).

The required sample size per group was calculated using the formula for a two-sample t-test:

Calculation formula: $$\:\frac{2{({z}_{1-\frac{\alpha\:}{2}}+{z}_{\beta\:})}^{2}\times\:{\sigma\:}^{2}}{\left|{\mu\:}_{T}-{\mu\:}_{c}\right|}=\:\frac{2{(1.96+1.28)}^{2}\times\:{1.5}^{2}}{{(-2)}^{2}}=11.81\approx\:12$$  

### Interventions

Participants in both groups will receive a total of 12 treatment sessions, administered twice a week for 6 consecutive weeks.

#### TBT group

The TBT treatment will be administered by a doctor of Korean Medicine with at least one year of clinical experience in the relevant field, following a standardized three-step process: To initiate treatment, the balance of the TMJ is assessed along three spatial axes—left-right (X), front-back (Y), and up-down (Z)—to detect any deviation. The primary goal is to determine a mandibular position that facilitates optimal alignment of the cervical spine, thereby stabilizing the positional relationship between the mandible and the maxilla. This process begins with a series of clinical assessments, including the restricted cervical rotation test, lateral cervical tension test, and cervical palpation, to evaluate bilateral muscle tension and cervical mobility. To assist in identifying the optimal balance point, a 1.6 mm-thick silicone bar (Jin Biotech, Cheonan, Korea) is used to establish an appropriate freeway space. The optimal mandibular position is defined as the position in which cervical rotation becomes smooth and symmetrical. This individualized assessment is essential for the fabrication of a CBA, which is used in the next step to temporarily fix the mandible in its ideal three-dimensional balance position.

Once the optimal mandibular position is identified, a CBA is fabricated to temporarily fix the jaw in that position. At this position, the CBA is created using a combination of two addition-type polyvinyl silicone impression materials (Exafine Putty, GC Dental Products Corp., Japan). While the patient is biting the silicone bars with both molars, the mixed impression material is molded directly onto the teeth to capture the correct mandibular position. After sitting for approximately 1 ~ 2 min, the hardened appliance (CBA) is ready for clinical use [26].

While the patient is wearing CBA, myofascial release will be performed 1 to 3 times. The practitioner will confirm the fascial relaxation response through palpation. This will be followed by a standardized set of jaw stretching exercises, which will be performed 5 times to enhance jaw mobility and relaxation [26].

#### TENS group

TENS will be administered to the control group. The procedure will be performed by a licensed Korean Medicine doctor with at least one year of clinical experience. The silicone electrode pads will be attached to the masseter muscle area on the affected side. If pain is present bilaterally, both sides will be treated. Low-frequency stimulator, H-3000-P (Hanil TM Co., Ltd., Korea), will be used for the stimulation. The stimulation frequency will be set at 3 Hz, and the intensity will be set so that the participant perceives a sensory stimulation without experiencing excessive muscle contraction or pain. Each TENS session will last for 15 min.

### Outcome measures

#### Primary outcome: visual analog scale

The primary outcome measure is the average temporomandibular joint pain Visual Analog Scale (VAS, 100 mm) score over the past week. It will be assessed at Week 0 (Baseline) and Week 6 (Primary endpoint).

#### Secondary outcomes

##### Vertical mandibular opening

The functional opening capacity of the TMJ will be evaluated by measuring both the pain-free opening and the maximum unassisted opening in millimeters (mm). Measurements will be taken at baseline, week 6, and week 10. Changes across these time points will be compared to assess improvements in TMJ range of motion and pain reduction.

##### Jaw functional limitation scale-8 (JFLS-8)

The JFLS-8 is an 8-item questionnaire that assesses the degree of jaw functional limitation in daily activities such as chewing, speaking, and facial expression. Each item is scored on a 0–10-point scale, with higher scores indicating greater limitation. Changes in total scores from baseline to weeks 6 and 10 will be analyzed to evaluate the persistence of treatment effects.

##### DC/TMD chronic pain grade scale 2.0

This scale comprehensively measures the severity of chronic pain related to temporomandibular disorders, including pain intensity, functional impact, and psychological factors. Grades will be assessed at baseline, week 6, and week 10, and changes over time will be analyzed to determine the effectiveness of pain management.

##### Treatment expectancy scale

Patients’ expectations regarding the effectiveness of treatment will be measured using a Likert scale (e.g., 1–5 points). The differences between pre-treatment expectations and actual experiences at weeks 6 and 10 will be compared to assess treatment satisfaction and the relationship between expectation and outcome.

##### Patient global impression of change (PGIC)

The PGIC is a 7-point scale used to evaluate the patient’s overall perception of improvement or worsening in health status (1 = very much worse, 7 = very much improved). Changes in PGIC scores at weeks 6 and 10 will be analyzed to assess the subjective effectiveness of the intervention.

### Safety assessment

For safety assessment, the occurrence of adverse events (AEs) and their potential relationship with the intervention (TBT or TENS in the control group) will be evaluated. The safety of the intervention methods will also be assessed through the measurement of vital signs and participant interviews regarding any discomfort or safety concerns experienced following the intervention. Both vital signs and AEs will be monitored and assessed at each visit from weeks 1 to 6, as well as at the follow-up visit in week 10.

The criteria and methods for safety evaluation include the documentation of all AEs and vital signs. Safety assessments will be conducted for all participants enrolled in the study (the intention-to-treat (ITT) population). Any undesirable medical findings or symptoms not observed before the start of the clinical trial will be classified as AEs.

In preparation for any adverse symptoms that may arise during the study period, appropriate medical actions will be taken if any new symptoms, not observed before the initiation of the clinical trial, occur. The investigators will record the date of onset, severity, outcome of the AE, actions taken regarding the intervention, the causal relationship between the AE and the intervention, any other suspected medications, and treatments provided for the AE. The principal investigator will ensure continuous follow-up until the AE resolves or the participant’s condition stabilizes.

### Cost-effectiveness evaluation

A cost-effectiveness evaluation will be conducted based on the cost and outcome data collected during this study to compare the cost-effectiveness between the intervention group and the control group. The primary analysis period is set at 10 weeks (corresponding to the study’s follow-up duration). If projection beyond these 10 weeks is required, long-term modeling such as a Markov model will be used. When the total analysis period (time horizon) exceeds 12 months, all costs will be standardized to 2024 Korean Won (KRW), and a 4.5% annual discount rate will be applied, based on the HIRA economic evaluation guidelines.

Effectiveness will be estimated using Quality-Adjusted Life Years (QALYs) and individual clinical outcome indicators measured according to the clinical trial schedule after randomization. QALYs will be measured using the validated Korean version of EQ-5D-5 L and calculated using the Korean-specific tariff developed by Kim et al. (2016) [27]. Individual effectiveness indicators will be derived from the primary and secondary clinical outcome measures as defined in the trial protocol.

Cost estimation will be performed using a micro-costing approach that multiplies the unit cost by usage volume for each cost item. Institutional data on resources and time required for TBT and TENS procedures will be collected internally. Participant-level cost data will be collected through surveys administered at Week 0, Week 6, and Week 10, capturing costs incurred over the preceding six weeks. Productivity losses will be assessed using the WPAI: GH 2.0 instrument, which evaluates productivity loss over the previous seven days. These results will be extrapolated using interpolation to estimate productivity losses over six weeks.

The analysis will be conducted from both the healthcare system perspective (including direct medical costs) and the societal perspective (including direct medical costs, non-medical costs, and productivity losses). Because the primary analysis period is less than one year, discounting will not be applied.

### Assignment of interventions

#### Allocation

The randomization process will be managed by a statistician who is not involved in the conduct or assessment of the clinical trial. The randomization officer will generate the randomization list using a specifically planned and reproducible method, employing Python 3.12.4 (Python Software Foundation, Wilmington, DE, USA) to ensure equal probability of assignment for each participant. Block randomization will be used to maintain balance between groups, with participants allocated to the intervention (TBT) and control (TENS) groups in a 1:1 ratio.

The randomization list will be sealed and stored in a manner that allows confirmation of whether the seal has been broken, and will be managed separately by the principal investigator. For allocation, the study coordinator will open the randomization envelopes sequentially in front of the participant at the time of assignment. Each opened envelope will have the date of opening and the study coordinator’s signature recorded and will be stored separately for documentation.

Participants who provide written informed consent and undergo screening will be assigned a screening number in the format DJ-S-001 (where DJ refers to Daejeon University Daejeon Korean Medicine Hospital, S indicates Screening, and 001 is the serial number). Only those who meet the inclusion and exclusion criteria after the eligibility assessment will be assigned a subject identification code in the format DJ-E-001 (where E indicates Enrollment).

#### Blinding

This clinical trial will be conducted in an assessor-blinded (single-blind) manner. The outcome assessors will remain blinded to group assignments until the completion of the trial, while participants and treating investigators will be aware of the assigned interventions (TBT or TENS). The investigators responsible for outcome assessment will not participate in the randomization process and will not be informed of the group allocation or the type of intervention the participant receives.

The interventions for both the TBT and TENS groups will be performed under standardized procedures and in similar environments to minimize bias. Subject identification codes will be designed to prevent assessors from distinguishing the assigned group. The assignment and management of identification codes will be handled by the study staff and will not be disclosed to the assessors until the trial is complete, except in emergencies where unblinding is necessary for participant safety. All procedures for randomization, allocation, and blinding will be thoroughly documented to ensure transparency and reproducibility.

### Data management and monitoring

Data management and monitoring will be conducted under the study protocol, institutional standard operating procedures (SOPs), Good Clinical Practice (GCP) guidelines, and applicable regulatory requirements. The principal investigator will report AEs and study progress periodically to the sponsor, who will oversee trial conduct through scheduled evaluations.

Monitoring will be conducted by a designated monitor through regular site visits and phone communications to ensure protocol compliance. This includes reviewing original medical records and research documents, as well as consulting with investigators as needed. In this trial, the clinical trial director will initiate monitoring after the enrollment of the first subject and subsequently after every 15 subjects are recruited.

All participant-related documents will use subject identification codes instead of personal names to protect confidentiality. These documents will be securely stored in compliance with data protection regulations. Upon study completion, all research documents will be archived at the trial site for three years following the final study report. Data collected during the trial will be securely stored and managed using an electronic Case Report Form (eCRF) system, ensuring data integrity and confidentiality.

### Statistical analysis

The primary analysis will be performed on the FAS, which will be defined under the ITT principle. In this study, all randomized participants will be analyzed as allocated, except for those who (1) do not meet major inclusion criteria, (2) never receive any intervention specified in the protocol, or (3) have no post-randomization data due to lack of any assessment. The PP set will include participants who complete the study without major protocol violations, such as premature discontinuation of the intervention, violation of inclusion/exclusion criteria, overall intervention compliance below 66.6%, or other significant protocol deviations.

The efficacy of the intervention will be primarily evaluated using the FAS population, with supportive analyses conducted on the PP set. In the event of missing data, the amount and mechanism of missingness will be assessed, and appropriate imputation methods will be selected and applied. All statistical tests will be two-sided, with a significance level of 5%. For all primary and secondary outcomes, point estimates of treatment effects and their corresponding 95% confidence intervals (CIs) will be reported. Effect sizes (e.g., Cohen’s d for continuous variables) will also be presented where appropriate to indicate the magnitude of the observed effects.

Demographic and baseline clinical characteristics will be summarized by treatment group. Continuous variables will be presented as means and standard deviations, and analyzed using Student’s independent t-test or Wilcoxon rank-sum test, depending on normality. Categorical variables will be summarized as frequencies and percentages, and analyzed using Pearson’s chi-squared test or Fisher’s exact test, as appropriate.

All statistical analyses will be performed using Python (version 3.12.4) with its statistical libraries, including statsmodels and scipy. The random assignment will also be conducted using Python (version 3.12.4).

#### Baseline characteristics

Baseline demographic and clinical characteristics of participants will be summarized using descriptive statistics (e.g., mean ± standard deviation for continuous variables, frequencies and percentages for categorical variables). Baseline characteristics will be compared between the two groups to ensure comparability.

#### Primary outcome analysis

The primary outcome, the mean change in TMJ pain VAS from baseline to Week 6 between the TBT and TENS groups, will be analyzed using an Analysis of Covariance (ANCOVA), with the baseline VAS score included as a covariate. The primary analysis will be conducted on an intention-to-treat (ITT) basis, including all randomized participants. In the event of missing values, multiple imputation using chained equations will be employed, assuming data are missing at random (MAR).

#### Secondary outcome analysis

The secondary outcomes include average TMJ pain VAS scores, worst TMJ pain VAS scores, vertical temporomandibular joint opening, including pain-free opening and maximum unassisted opening, JFLS-8 scores, GCPS 2.0 scores, Treatment Expectancy Scale scores, PGIC scores at 0, 6, and 10 weeks. For continuous outcomes measured at multiple time points (e.g., VAS scores at 0, 6, and 10 weeks), linear mixed models will be used to assess within-group and between-group changes over time. Categorical outcomes will be analyzed using chi-square tests.

#### Safety analysis

AEs will be recorded at each visit through spontaneous reporting and systematic questioning. The incidence, severity, and causality (relatedness to intervention) of AEs will be summarized using descriptive statistics and compared between groups using Fisher’s exact test or chi-square test.

#### Cost-effectiveness analysis

Cost-effectiveness evaluation will be primarily based on the full analysis set (FAS), with a per-protocol (PP) analysis conducted to assess sensitivity to missing data. Estimation of utility and cost will follow the same approach used in the clinical effectiveness evaluation, with missing data handled using appropriate imputation methods based on the identified mechanisms.

The decision tree model will be employed as the primary modeling framework for the cost-effectiveness evaluation. As economic evaluation indices, the Incremental Cost-Utility Ratio (ICUR), based on the EQ-5D-5 L results and cost data, and the Incremental Cost-Effectiveness Ratio (ICER), based on individual clinical outcome indicators and cost data, are calculated using the following formulas:

$$\:\mathrm{I}\mathrm{C}\mathrm{U}\mathrm{R}=\frac{\mathrm{T}\mathrm{o}\mathrm{t}\mathrm{a}\mathrm{l}\:{\mathrm{C}\mathrm{o}\mathrm{s}\mathrm{t}}_{\mathrm{C}\mathrm{P}\:\mathrm{g}\mathrm{r}\mathrm{o}\mathrm{u}\mathrm{p}}-\:\mathrm{T}\mathrm{o}\mathrm{t}\mathrm{a}\mathrm{l}\:{\mathrm{C}\mathrm{o}\mathrm{s}\mathrm{t}}_{\mathrm{U}\mathrm{s}\mathrm{u}\mathrm{a}\mathrm{l}\:\mathrm{C}\mathrm{a}\mathrm{r}\mathrm{e}\:\mathrm{G}\mathrm{r}\mathrm{o}\mathrm{u}\mathrm{p}}}{{\mathrm{Q}\mathrm{A}\mathrm{L}\mathrm{Y}}_{\mathrm{C}\mathrm{P}\:\mathrm{g}\mathrm{r}\mathrm{o}\mathrm{u}\mathrm{p}}-\:{\mathrm{Q}\mathrm{A}\mathrm{L}\mathrm{Y}}_{\mathrm{U}\mathrm{s}\mathrm{u}\mathrm{a}\mathrm{l}\:\mathrm{C}\mathrm{a}\mathrm{r}\mathrm{e}\:\mathrm{G}\mathrm{r}\mathrm{o}\mathrm{u}\mathrm{p}}}$$

$$\:\mathrm{I}\mathrm{C}\mathrm{E}\mathrm{R}\:=\frac{\mathrm{T}\mathrm{o}\mathrm{t}\mathrm{a}\mathrm{l}\:{\mathrm{C}\mathrm{o}\mathrm{s}\mathrm{t}}_{\mathrm{C}\mathrm{P}\:\mathrm{g}\mathrm{r}\mathrm{o}\mathrm{u}\mathrm{p}}-\mathrm{T}\mathrm{o}\mathrm{t}\mathrm{a}\mathrm{l}\:{\mathrm{C}\mathrm{o}\mathrm{s}\mathrm{t}}_{\mathrm{U}\mathrm{s}\mathrm{u}\mathrm{a}\mathrm{l}\:\mathrm{C}\mathrm{a}\mathrm{r}\mathrm{e}\:\mathrm{G}\mathrm{r}\mathrm{o}\mathrm{u}\mathrm{p}}}{{\mathrm{E}\mathrm{f}\mathrm{f}\mathrm{e}\mathrm{c}\mathrm{t}}_{\mathrm{C}\mathrm{P}\:\mathrm{g}\mathrm{r}\mathrm{o}\mathrm{u}\mathrm{p}}-{\mathrm{E}\mathrm{f}\mathrm{f}\mathrm{e}\mathrm{c}\mathrm{t}}_{\mathrm{U}\mathrm{s}\mathrm{u}\mathrm{a}\mathrm{l}\:\mathrm{C}\mathrm{a}\mathrm{r}\mathrm{e}\:\mathrm{G}\mathrm{r}\mathrm{o}\mathrm{u}\mathrm{p}}}$$

One-way and probabilistic sensitivity analyses will be conducted to assess the robustness of the results. In the sensitivity analysis, deterministic sensitivity analysis will be performed for all relevant parameters and visualized through a tornado diagram, followed by probabilistic sensitivity analysis using parameter distributions and representative values.

## Ethics and dissemination

The clinical trial will be performed under the Declaration of Helsinki, Korean GCP Guidelines, related laws, and protocols. This study was approved by the Institutional Review Board (IRB) of Daejeon University Daejeon Korean Medicine Hospital (approval number: DJDSKH-24-BM-13-1) on 4 November 2024. This study was registered in April 2025 at the National Clinical Trial Registry Clinical Research Information Service (https://cris.nih.go.kr) with the identifier number KCT0010382.

## Discussion

This study protocol outlines a single-center, randomized, assessor-blinded, parallel-group clinical trial designed to evaluate the effectiveness, safety, and cost-effectiveness of TBT compared to TENS for patients with TMD. Given the increasing prevalence and economic burden of TMD, coupled with the recognized gaps in current clinical practice guidelines regarding intraoral balancing appliances, this trial is poised to contribute significantly to the evidence base for TMD management.

The rising trend in TMD patient numbers and healthcare costs in South Korea underscores the urgent need for effective and economically viable treatment options. While various conventional treatments exist, the limitations of current pharmacotherapies (e.g., side effects of NSAIDs, muscle relaxants, antidepressants) and the uncertain evidence for widely used interventions like occlusal splints highlight the necessity for exploring alternative, evidence-based therapies. Our study addresses a critical unmet need by focusing on TBT, a novel Korean medicine approach utilizing intraoral balancing appliances, which has yet to be assessed through a randomized controlled trial.

A key strength of this protocol lies in its design, including randomization and assessor blinding, which aims to minimize bias and enhance the internal validity of the findings. The comprehensive set of outcome measures, encompassing pain intensity, jaw function, chronic pain grading, patient-reported outcomes, and a thorough economic evaluation, will provide a holistic understanding of TBT’s impact. Furthermore, the inclusion of an economic feasibility analysis is particularly relevant in the context of rising healthcare expenditures, offering insights into the cost-effectiveness of TBT in a real-world clinical setting. The use of a standard TENS therapy as a control group, a commonly utilized and relatively well-established physical therapy modality for TMD, allows for a direct comparison with a widely accepted intervention.

However, several practical and operational difficulties must be examined during this trial. As a single-center study, the generalizability of our findings to a larger population or different clinical settings might be limited. We intend to address this by clearly defining inclusion and exclusion criteria to ensure a homogeneous patient population. To further address the generalizability, our research team is also planning a multi-center registry study, a prospective observational study, involving various Korean medical clinics and hospitals that currently implement TBT. This will allow for the collection of real-world data from a more diverse patient population and across different clinical environments, thereby enhancing the external validity of TBT’s effects.

Participant and practitioner blinding is not feasible given the nature of the interventions, which could introduce some performance or detection bias. To address this, we have implemented assessor blinding for all outcome measurements and standardized treatment protocols to ensure consistency in intervention delivery. However, it is important to acknowledge that the lack of participant and practitioner blinding might lead to differential treatment expectancy or performance bias between groups, potentially influencing subjective outcomes. While objective measures are less susceptible, the interpretation of patient-reported outcomes (e.g., VAS, PGIC, Treatment Expectancy Scale) should consider this limitation.

In conclusion, this protocol describes a meticulously designed randomized controlled trial that will provide crucial evidence on the efficacy, safety, and economic viability of TBT for TMD. The findings are expected to inform future clinical practice guidelines, particularly those related to Korean medicine interventions for TMD, and offer valuable insights for clinicians and policymakers seeking effective and sustainable treatment strategies.

### Trial status

Protocol version number: 1.3, date: 13 January 2025.

The date recruitment began: 11 February 2025.

The approximate date when recruitment will be completed: 30 June 2025.

## Supplementary Information

Supplementary Material 1. S1 Appendix. SPIRIT checklist.

Supplementary Material 2. S2 Appendix. The institutional review board-approved protocol (Korean & English translation).

## Data Availability

All data necessary to support the findings of this study are available within the manuscript and/or its supplementary information files. The full study protocol, as approved by the institutional review board, is provided as a supplementary file (S2 Appendix). The findings of this study will be disseminated through publication in a peer-reviewed scientific journal. An anonymized participant-level dataset and statistical code used for the analyses will be made available from the corresponding author upon reasonable request, provided that the request aligns with ethical guidelines and participant privacy protection. This data will be available after the publication of the main study results.

## Abbreviations

AEs: Adverse Events
ANCOVA: Analysis of Covariance
BTX: Botulinum toxin
C2: Axis (C2 cervical vertebra)
CBA: Customized Balancing Appliance
CBT: Cognitive Behavioral Therapy
CEAC: Cost-Effectiveness Acceptability Curve
CIs: Confidence Intervals
CRIS: Clinical Research Information Service
DC/TMD: Diagnostic Criteria for Temporomandibular Disorders
eCRF: electronic Case Report Form
EMR: Electronic Medical Records
EQ-5D: EuroQol Five Dimensions
EQ-VAS: EuroQol Visual Analogue Scale
FAS: Full Analysis Set
FCST: Functional Cerebrospinal Therapy
GCP: Good Clinical Practice
GCPS 2.0: Graded Chronic Pain Scale 2.0
HIRA: Health Insurance Review & Assessment Service
ICER: Incremental Cost-Effectiveness Ratio
ICF: Informed Consent Form
ICUR: Incremental Cost-Utility Ratio
IADR: International Association for Dental, Oral, and Craniofacial Research
INfORM: International Network for Orofacial Pain and Related Disorder Methodology
IRB: Institutional Review Board
ITT: Intention-To-Treat
JFLS-8: Jaw Functional Limitation Scale-8
KRW: Korean Won
LLLT: Low-Level Laser Therapy
MAR: Missing at Random
MFDS: Ministry of Food and Drug Safety
NSAIDs: Non-Steroidal Anti-Inflammatory Drugs
O-C2: Occiput-C2
PGIC: Patient Global Impression of Change
PP: Per-Protocol
QALYs: Quality-Adjusted Life Years
RCT: Randomized Controlled Trial
SAEs: Serious Adverse Events
SD: Standard Deviation
SOPs: Standard Operating Procedures
SPIRIT: Standard Protocol Items: Recommendations for Interventional Trials
TBT: Temporomandibular Joint Balancing Therapy
TENS: Transcutaneous Electrical Nerve Stimulation
TMJ: Temporomandibular Joint
TMD: Temporomandibular Joint Disorder
VAS: Visual Analog Scale
WPAI: GH 2.0:Work Productivity and Activity Impairment: General Health 2.0
