# Supplementary material for: The efficacy, safety, and cost-effectiveness of TMJ balancing therapy for temporomandibular joint disorder: study protocol for a randomized controlled trial
Source: BMC Complement Med Ther. 2026 Mar 11;26:146. doi: 10.1186/s12906-026-05303-8 (PMC13088731; doi:10.1186/s12906-026-05303-8)
Supplement: Supplementary file 2 — Supplementary Material 2. S2 Appendix. The institutional review board-approved protocol (Korean & English translation). [file 12906_2026_5303_MOESM2_ESM.pdf]

PROTOCOL

- 임상시험계획서 -

턱관절 장애에 대한 TBT의 유효성, 안전성, 경제성 평가를 위한 무작위 대조군 임상시험

A Randomized Controlled Clinical Trial to Evaluate the Effectiveness, Safety, and Economics of TBT for Temporomandibular Disorder

**Protocol No.**	**DJDSKH-TD-01**
**Protocol Ver.**	**Ver. 1.3**
**임상시험 의뢰기관**<br>Requesting agency	**보건산업진흥원**<br>Korea Health Industry Development Institute
**임상시험 실시기관**<br>Executing agency	**대전대학교 대전한방병원**<br>Daejeon Korean Medicine Hospital of Daejeon University
**시험책임자**<br>Principal investigator	**유호룡**<br>Horyong Yoo
**발행 일자**<br>Date	**2025.01.13**

대전대학교 대전한방병원 임상시험센터
DAEJEON UNIVERSITY DAEJEON KOREAN MEDICINE HOSPITAL
CLINICAL TRIAL CENTER

개요 (Synopsis)

임상시험 제목<br>Clinical trial title	[국문] 턱관절 장애에 대한 TBT의 유효성, 안전성, 경제성 평가를 위한 무작위 대조군 임상시험<br>[영문] A Randomized Controlled Clinical Trial to Evaluate the Effectiveness, Safety, and Economics of TBT for Temporomandibular Disorder
임상시험 목적<br>Clinical trial purpose	턱관절 장애에 대한 TBT의 유효성, 안전성, 경제성 평가<br>To Evaluate the Effectiveness, Safety, and Economics of TBT for Temporomandibular Disorder
임상시험 실시기관명 및 주소<br>Name&address of clinical trial executing agency	대전대학교 대전한방병원. 대전광역시 서구 대덕대로 176번길 75.<br>전화번호: 042-470-9131<br>Daejeon Korean Medicine Hospital of Daejeon University. 75, Daedeok-daero 176beon-gil, Seo-gu, Daejeon.<br>Phone: 042-470-9131
임상시험 책임자<br>Clinical trial principal investigator	유호룡 대전대학교 한의학과 한방내과학 교수<br>HoRyong Yoo, Professor of Internal Korean Medicine, Daejeon University department of Korean Medicine
대상질환<br>대상질환<br>Target disease	턱관절 장애<br>Temporomandibular Disorder
임상시험 기간<br>Clinical trial duration	본 연구의 기간은 본 연구계획서에 대한 기관생명윤리위원회(IRB) 승인일로부터 12개월로 한다. 단, 피험자 등록에 따라 단축되거나 연장될 수 있다.<br>This study will last 12 months from the date this research plan was approved by the institutional review board (IRB). However, depending on the subject registration, it may be shortened or extended.
임상시험 디자인<br>Clinical trial design	단일기관, 무작위배정, 평가자 맹검, 평행설계 임상시험<br>Single-center, randomized, evaluator-blinded, parallel clinical trial
대상자 선정기준<br>Inclusion criteria	1) 만 19세 이상 70세 이하의 남녀<br>2) DC/TMD 기준에 따라 턱관절 편측 또는 양측에 3개월 이상 지속되는 간헐적인 통증을 호소하는 자<br>3) 턱관절 통증을 호소하는 부위(양측 통증 환자의 경우 통증이 심한 방향 기준)의 통증이 지난 1주일간 평균적으로 VAS 40mm 이상인 경우<br>4) 턱의 움직임, 기능 및 주변 기능에 의해 발생하는 턱관절 및 주변부(관자놀이, 귓속, 귀 앞)의 국소적인 통증이 있는 경우<br>5) 검사자가 촉진을 통해 관자근, 깨물근, 또는 하악 관절의 관절 융기 외측극 부위에서 통증을 확인하여 근육통, 근막통증 또는 관절통 중 하나의 타입에 해당하는 경우; 또는 턱의 움직임 시 발현되거나 악화되는 두통이 측두근 부위에 위치하며 검사 시 재현 가능한 경우<br>1) Men and women aged 19 to 70 years.<br>2) Individuals who report intermittent pain in the temporomandibular joint (unilateral or bilateral) lasting for more than 3 months based on the DC/TMD criteria.<br>3) Individuals whose pain in the temporomandibular joint (based on the side with the most severe pain for patients with bilateral pain) has an average Visual Analog Scale (VAS) score of 40 mm or higher over the past week.<br>4) Individuals with localized pain in the temporomandibular joint and surrounding areas (temple, inside the ear, or in front of the ear) associated with jaw movement, function, or related activities.<br>5) Individuals whose pain is confirmed by the examiner through

	palpation in the temporalis muscle, masseter muscle, or the lateral pole of the mandibular joint's articular prominence, corresponding to one of the following types: myalgia, myofascial pain, or arthralgia; or individuals with headaches located in the temporalis area that are reproducible during examination and are triggered or worsened by jaw movement.
대상자 제외기준<br>Exclusion criteria	1) 양악수술 등 턱관절 관련 수술을 받은 자<br>2) 치료 효과나 결과의 해석을 방해할 수 있는 다발성 통증 질환(예: 류마티스 관절염), 신경계 질환(예: 뇌종양, 뇌졸중, 삼차신경통 등)이 있는 경우<br>3) 현재 스테로이드제제, 면역억제제, 정신질환 약물 또는 연구 결과에 영향을 줄 수 있는 기타 약물을 복용하고 있는 경우<br>4) 1개월 이내에 비스테로이드성 소염제 등의 통증에 영향을 줄 수 있는 약물 복용을 시작 또는 중단한 경우<br>5) 기타 의사의 판단 하에 본 연구 참여가 적합하지 않은 자 (신체 내 전기기기, pacemaker 등을 시술 받은 자, 관절 침범 양상을 확인하였을 때 류마티스 관절염의 양상이 뚜렷하게 나타나는 환자 등)<br>6) 임산부 또는 수유부<br>1) Individuals who have undergone temporomandibular joint-related surgeries, such as orthognathic surgery.<br>2) Individuals with multiple pain disorders (e.g., rheumatoid arthritis) or neurological conditions (e.g., brain tumor, stroke, trigeminal neuralgia) that could interfere with the interpretation of treatment effects or outcomes.<br>3) Individuals currently taking steroids, immunosuppressants, psychiatric medications, or other drugs that could affect study results.<br>4) Individuals who have started or discontinued medications affecting pain, such as nonsteroidal anti-inflammatory drugs, within the past month.<br>5) Individuals deemed unsuitable for participation by the investigator (e.g., those with implanted electrical devices or pacemakers, or those showing clear signs of rheumatoid arthritis upon joint involvement assessment).<br>6) Pregnant or lactating women.
방문일정<br>Visit schedule	총 10주 동안 14회 방문[screening(10일 이내), 6주간 주 2회 방문, 10주 후 관찰방문]<br>A total of 14 visits over 10 weeks [screening (within 10 days), visits twice a week for 6 weeks, observation visit after 10 weeks]
임상시험 대상자의 수 및 산정 근거<br>Number of clinical trial subjects and calculation method	1) 대상자 수: 시험군 15명, 대조군 15명, 총 30명<br>2) 산출 근거:<br>① 본 임상시험의 가설은 다음과 같다.<br>- H0(귀무가설): μt = μc vs. H1(대립가설): μt ≠ μc - μt : 치료군(TBT)의 베이스라인(baseline) 대비 6주 시점의 VAS (mm) 평균 변화값<br>- μc : 대조군의 베이스라인(baseline) 대비 6주 시점의 VAS (mm) 평균 변화값<br>- 평가변수의 통계적 가설검정: 양측 검정 - 유의수준(α): 0.05 - 제2종 오류(β): 0.1, 검정력(1-β): 90% - 시험군과 대조군의 비율은 1:1로 동일하게 한다.<br>② 본 연구와 연구 디자인, 처치의 기간, 방법 및 횟수, 평가변수 등이 가장 유사한 선행연구를 참고하여 계산하였다.<br>- 해당 논문에서는 치료군 15명, 대조군 15명으로 총 30명의 대상자를 모집하였다. 처치 기간은 10주였으며, 총 처치 횟수는 12회였다. 1차 유효성 평가변수는 VAS에 기준한 통증 강도였다. 치료군과 대조군의 VAS(평균(표준편차))는 치료 전 각각 5.60(0.91), 5.40(1.06)이었으며,

치료 후 각각 1.67(0.62), 4.20(0.78)로 나타났으며, 대조군 대비 치료군의 효과 차이(Mean Difference)는 –2.53이고, 합동표준편차(Pooled SD)는 0.96으로 나타났다.

③ 효과 차이는 –2, 표준편차는 1.5로 보수적인 값을 설정하여 대조군 대비 치료군의 효과를 확인하기 위한 시험대상자 수를 산출하는 경우 결과는 아래와 같이 군당 약 12명이 필요한 것으로 나타난다. 중도탈락률 20%를 고려하면 군당 15명씩 총 30명의 임상시험 대상자가 필요한 것으로 나타난다.

산출 공식: $\left\{\frac{2\left(z_{1-\frac{\alpha}{2}}+z_{\beta}\right)^{2}*\sigma^{2}}{\left|\mu_{T}-\mu_{c}\right|}\right\}=\left\{\frac{2(1.96+1.28)^{2}*1.5^{2}}{(-2)^{2}}\right\}=11.81\approx 12$

중도탈락률 20%를 고려: 11.81/0.8=14.76, 약 15명, 총 30명.

④ 참고 논문: Rezaie K, Amiri A, Ebrahimi Takamjani E, Shirani G, Salehi S, Alizadeh L. The Efficacy of Neck and Temporomandibular Joint (TMJ) Manual Therapy in Comparison With a Multimodal Approach in the Patients with TMJ Dysfunction: A Blinded Randomized Controlled Trial. Med J Islam Repub Iran. 2022;36:45. doi: 10.47176/mjiri.36.45. PMID: 36128309; PMCID: PMC9448471.

1) Number of subjects: experimental group (N = 15), control group (N = 15), total (N = 30)
2) Basis for Calculation:

① The hypothesis of this clinical trial is as follows:
- H0 (Null Hypothesis): μt = μc vs. H1 (Alternative Hypothesis): μt ≠ μc
- μt: Mean change in VAS (mm) at 6 weeks compared to baseline in the treatment group (TBT)
- μc: Mean change in VAS (mm) at 6 weeks compared to baseline in the control group
- Statistical hypothesis testing for the outcome variable: Two-sided test
- Significance level (α): 0.05
- Type II error (β): 0.1, Power (1-β): 90%
- The ratio of participants in the treatment and control groups is set to 1:1.

② The sample size was calculated with reference to a prior study that most closely resembles the present study in terms of study design, duration, treatment method and frequency, and outcome variables.
- In that study, a total of 30 participants were recruited (15 in the treatment group and 15 in the control group). The treatment period was 10 weeks, with a total of 12 treatment sessions. The primary efficacy outcome was pain intensity measured by VAS.
- The mean (standard deviation) VAS scores before treatment were 5.60 (0.91) for the treatment group and 5.40 (1.06) for the control group. After treatment, the scores were 1.67 (0.62) and 4.20 (0.78), respectively.
- The mean difference in effect between the treatment and control groups was –2.53, and the pooled standard deviation was 0.96.

③ A conservative effect size of –2 and standard deviation of 1.5 were used to calculate the required number of participants to confirm the treatment effect compared to the control group. The result showed that approximately 12 participants per group are needed. Considering a 20% dropout rate, a total of 30 participants (15 per group) are required.

	- Calculation formula:<br>$$\left\{\frac{2\left(z_{1-\frac{\alpha}{2}}+z_{\beta}\right)^{2}*\sigma^{2}}{\left|\mu_{T}-\mu_{c}\right|}\right\}=\left\{\frac{2(1.96+1.28)^{2}*1.5^{2}}{(-2)^{2}}\right\}=11.81\approx 12$$<br>Considering a 20% dropout rate: 11.81 / 0.8 = 14.76 → approximately 15 participants per group, total of 30 participants.<br>④ Reference paper: Rezaie K, Amiri A, Ebrahimi Takamjani E, Shirani G, Salehi S, Alizadeh L. The Efficacy of Neck and Temporomandibular Joint (TMJ) Manual Therapy in Comparison With a Multimodal Approach in the Patients with TMJ Dysfunction: A Blinded Randomized Controlled Trial. Med J Islam Repub Iran. 2022;36:45. doi: 10.47176/mjiri.36.45. PMID: 36128309; PMCID: PMC9448471.
시험 방법<br>Test Methods	1) 중재 기간 및 간격<br>- 지원자 중에서 임상시험에 대한 충분한 설명을 듣고 동의서에 서명한 후 선정기준 및 제외기준에 따라 본 임상시험에 참여하게 되는 대상자에게 대상자 식별코드를 부여하고, 시험군과 대조군으로 나누어 임상시험을 실시한다. 시험군에 속하는 대상자는 screening 후 6주간 총 12회(주 2회) 시험기관을 방문하여 TBT 치료를 받고, 시험 시작 전(baseline)과 6주, 10주에 유효성, 안전성 및 경제성을 평가하기 위한 검사를 받는다. 대조군에 속하는 대상자는 screening 후 6주간 총 12회(주 2회) 시험기관을 방문하여 TENS 치료를 받고, 시험군과 동일하게 시험 시작 전(baseline)과 6주, 10주에 유효성, 안전성 및 경제성을 평가하기 위한 검사를 받는다.<br>2) 시험군: TBT 치료(6주 동안 12회, 주 2회 실시)<br>3) 대조군: TENS 치료(6주 동안 12회, 주 2회 실시)<br>4) 다른 처치<br>- 시험 기간 동안 턱관절 장애에 대해 한의 치료를 제외한 약물, 비약물 요법 등 모든 병용치료를 허용함. 단, 약물을 복용하는 경우에는 최근 6주 동안 의사 처방을 받은 통증에 영향을 줄 수 있는 약물(예: 비스테로이드성 소염제)의 복용량이 일정해야 하고 시험 기간 동안 역시 일정하게 유지해야 함. 복용하고 있는 약물이나 다른 치료 및 시험 도중 변경되는 약물이나 치료에 대해서는 시험자가 확인하여 기록함. 또한, 시험 기간 동안에는 개인적으로 수행하는 운동은 허용하나, 의사의 처방에 따른 턱관절 장애에 대한 도수치료, 재활치료는 허용하지 않음.<br>5) 진단 및 시술자의 요건:<br>- 한의사 중 1년 이상의 해당 분야 임상경험이 있는 자<br>1) Intervention Duration and Frequency<br>- Among the applicants, those who receive a full explanation of the clinical trial and sign the informed consent form will be assigned a subject identification code based on the inclusion and exclusion criteria. These subjects will be randomly allocated to either the treatment or control group.<br>- Participants in the treatment group will receive TBT therapy 12 times over a 6-week period (twice a week) following screening. Evaluations for efficacy, safety, and cost-effectiveness will be conducted at baseline, week 6, and week 10.<br>- Participants in the control group will receive TENS therapy under the same schedule—12 sessions over 6 weeks (twice a week) following screening—and will undergo the same evaluations at baseline, week 6, and week 10 as the treatment group.<br>2) Treatment Group:<br>- TBT therapy (12 sessions over 6 weeks, twice per week)

	3) Control Group:<br>- TENS therapy (12 sessions over 6 weeks, twice per week)<br>4) Concurrent Treatments<br>- During the study period, all concurrent treatments for temporomandibular joint (TMJ) disorders, excluding Korean medicine interventions, are permitted. However, if the subject is taking medication, the dosage of any pain-related drugs (e.g., NSAIDs) prescribed within the last 6 weeks must be consistent and should remain unchanged during the trial period.<br>- The investigator must review and record any current medications or treatments, as well as any changes made during the study.<br>- Participants are allowed to engage in personal exercise during the trial, but manual therapy or rehabilitation for TMJ disorders prescribed by a physician is not permitted.<br>5) Qualifications of Diagnosticians and Practitioners:<br>- Korean medicine doctors with at least one year of clinical experience in the relevant field.
유효성 평가변수<br>Efficacy endpoints	1) 일차 유효성 평가변수(Primary Outcomes measure)<br>① 턱관절 통증 VAS(100mm): 연구 시작 전과 6주 후 VAS 점수의 변화<br>a. 지난 1주간의 평균적인 턱관절 통증<br>2) 이차 유효성 평가변수(Secondary Outcome measures)<br>① 턱관절 통증 VAS(100mm): 연구 시작 전과 6주, 10주 후 VAS 점수의 변화<br>a. 지난 1주간의 평균적인 턱관절 통증<br>b. 지난 1주간의 가장 심했던 턱관절 통증<br>② 수직 턱관절 개구도: 시험 시작 전(baseline*)과 6주, 10주 후 턱관절 개구도의 변화<br>a. Pain free opening<br>b. Maximum unassisted opening<br>③ 턱기능 제한지수(JFLS-8): 시험 시작 전(baseline)과 6주, 10주 후 턱기능 제한지수 점수의 변화<br>④ DC/TMD 만성통증 등급 척도 2판: 시험 시작 전(baseline)과 6주, 10주 후 만성통증 등급 척도 점수의 변화<br>⑤ 치료 기대 척도: 시험 시작 전(baseline)과 6주, 10주 후 치료 기대 척도 점수의 변화<br>⑥ 전반적 평가 척도(PGIC): 시험 시작 후(baseline)와 6주, 10주 후 전반적 평가 척도 점수의 변화<br><br>3) Cost-effectiveness outcome measures<br>① Quality-adjusted life years (QALYs): 총 연구기간(10주)에 대한 질보정수명<br>② Euroqol five-dimension scale (EQ-5D): 시험 시작 전(baseline)과 1~6주(Visit 1~8), 10주(Visit 9) 후 EQ-5D 점수의 변화<br>③ Euroqol visual analog scale (EQ-VAS): 시험 시작 전(baseline)과 1~6주(Visit 1~8), 10주(Visit 9) 후 EQ-VAS 점수의 변화<br>④ 의료비용(공식적 의료비용 및 비공식적 의료비용), 생산성손실비용(이환비용): 총 연구기간(10주)에 발생한 총 비용 및 항목별 비용<br>⑤ Incremental cost-effectiveness ratio(ICER, cost per QALYs)<br><br>* Baseline: 방문1<br>6주(Treatment phase): 시험 시작 후 6주 후<br>10주(Follow-up phase): 시험 시작 후 10주 후(추적관찰)

	1) Primary Outcome Measure<br>① Temporomandibular Joint (TMJ) Pain VAS (100 mm): Change in VAS score from baseline to 6 weeks<br>a. Average TMJ pain over the past week<br><br>2) Secondary Outcome Measures<br>① TMJ Pain VAS (100 mm): Change in VAS score from baseline to 6 weeks and 10 weeks<br>a. Average TMJ pain over the past week<br>b. Worst TMJ pain experienced over the past week<br>② Vertical Mandibular Opening: Change in mandibular opening from baseline to 6 and 10 weeks<br>a. Pain-free opening<br>b. Maximum unassisted opening<br>③ Jaw Functional Limitation Scale (JFLS-8): Change in JFLS-8 score from baseline to 6 and 10 weeks<br>④ DC/TMD Chronic Pain Grade Scale (Version 2): Change in chronic pain grade from baseline to 6 and 10 weeks<br>⑤ Treatment Expectation Scale: Change in treatment expectation scores from baseline to 6 and 10 weeks<br>⑥ Patient Global Impression of Change (PGIC): Change in PGIC scores from baseline to 6 and 10 weeks<br><br>3) Cost-effectiveness Outcome Measures<br>① Quality-Adjusted Life Years (QALYs): QALYs over the total study period (10 weeks)<br>② EuroQol Five-Dimension Scale (EQ-5D): Change in EQ-5D scores from baseline to weeks 1–6 (Visit 1–8) and week 10 (Visit 9)<br>③ EuroQol Visual Analog Scale (EQ-VAS): Change in EQ-VAS scores from baseline to weeks 1–6 (Visit 1–8) and week 10 (Visit 9)<br>④ Medical Costs (formal and informal) and Productivity Loss Costs (morbidity costs): Total and itemized costs incurred during the 10-week study period<br>⑤ Incremental Cost-Effectiveness Ratio (ICER): Cost per QALY gained<br><br>* Baseline: Visit 1<br>6 weeks (Treatment Phase): 6 weeks after the start of the study<br>10 weeks (Follow-up Phase): 10 weeks after the start of the study (follow-up)
안전성 평가변수<br>Safety endpoints	1) 이상반응 평가<br>- 이상반응 유무 및 중재 방법과의 관련 여부 평가<br>2) 활력징후 측정<br>- 활력징후 측정을 통한 중재 방법의 안전성 평가<br>3) 면담 시행<br>- 중재 이후 불편감 및 중재 방법의 안전성을 평가<br><br>1) Adverse Event Assessment – Evaluation of the presence or absence of adverse events and their potential relationship to the intervention method<br>2) Vital Signs Measurement – Assessment of the safety of the intervention method through measurement of vital signs<br>3) Interview Assessment – Evaluation of discomfort and safety of the intervention method following the intervention through participant interviews

통계분석<br>Statistical analysis	1) 유효성 분석 방법<br>① 1차 유효성 평가변수<br>- 시험군과 대조군 간의 평균 차이의 추정값과 95% 신뢰구간, 그리고 p-value 값을 제시한다.<br>- 무작위 배정을 받은 시험대상자 중 적어도 한 번 이상 중재를 받고 시험 전과 중재 시술 이후 적어도 한 번 이상 VAS 점수(a. 지난 1주간의 평균적인 턱관절 통증)가 측정된 대상자를 분석군에 포함한다. VAS 점수의 결측이 발생한 경우, 결측량과 기전에 대한 진단을 먼저 시행한 후 적합한 imputation 방법을 선정하여 FAS 분석을 시행한다.<br>- VAS 점수의 변화에 대한 두 군의 차이를 independent t test를 통해 검증한다. 기저 값의 유의한 차이가 있는 경우는 이를 보정한 공분산 분석을 시행하고, 기타 기저 변수의 유의한 차이를 보정하기 위해서는 다중회귀분석을 시행한다.<br>- VAS 점수에 대한 반복 측정된 값을 대상으로 반복측정분산분석을 통해 시간과 치료간의 교호작용을 검정한다.<br><br>② 2차 유효성 평가변수<br>- VAS 점수, 수직 턱관절 개구도, 턱기능 제한지수(JFLS-8), DC/TMD 만성통증 등급 척도 2판, 치료 기대 척도, 전반적 평가 척도(PGIC)는 공분산분석, 혹은 기저조사의 변수를 보정한 다중회귀분석을 사용하여 분석한다. 이때, 시험 시작 전 점수가 측정된 경우는 이를 공변량으로 하며, 결측치에 대한 별도의 처리는 하지 않는 per-protocol 분석을 시행한다.<br><br>2) 경제성평가 분석<br>- 분석 기간: 기본 분석 기간은 10주로 시행하며, 이후의 기간에 대한 추정이 필요할 경우 Markov model 등을 이용하여 장기 비용과 효과를 모델링한다.<br>- 모형: Decision tree model (결정수형모형)<br>- 관점: 보건의료체계 관점 및 사회적 관점<br>- 질보정수명(QALY): EQ-5D로 도출된 삶의 질을 주 평가변수로 사용한다(Area under the curve method).<br>- 점증적 비용효용비(incremental cost-utility ratio, ICUR):<br>EQ-5D-5L 결과 및 비용 평가 결과를 바탕으로 다음과 같이 산출한다.<br>$ICUR = \frac{전체비용_{CP적용군} - 전체비용_{일반치료군}}{질보정수명_{CP적용군} - 질보정수명_{일반치료군}}$<br>- 점증적 비용효과비(incremental cost-effectiveness ratio, ICER):<br>개별 유효성 지표 및 비용 평가 결과를 바탕으로 다음과 같이 산출한다.<br>$ICER = \frac{전체비용_{CP적용군} - 전체비용_{일반치료군}}{효과_{CP적용군} - 효과_{일반치료군}}$<br>- 기본 분석: 연구에서 수집되는 모수(parameter)들의 대푯값(평균 등)을 사용하여 시행한다.<br>- 민감도 분석: 가능한 모든 모수들에 대하여 일원민감도분석(deterministic sensitivity analysis)을 시행하여 토네이도 다이어그램을 제시하고, 가능한 모든 모수들의 분포 및 대푯값을 사용하여 확률적 민감도분석(probabilistic sensitivity analysis)을 시행한다.<br><br>3) 안전성 평가변수<br>- 이상반응의 빈도, 발현율, 각각의 목록, 상세한 발현시간, 심각한 정도

및 시험약물과의 인과관계 등을 제시하며, 필요한 경우 그래프 형태로 보고한다. 통계적 분석이 필요한 경우는 변수의 특성과 목적에 따라 paired t-test, Mcnemar test, ANOVA, independent t-test, chi-square test 혹은 Fisher's exact test 등을 실시한다.

1) Efficacy Analysis Methods

① Primary Outcome Measure

- The estimated mean difference between the treatment and control groups, along with a 95% confidence interval and p-value, will be presented.
- The analysis set will include participants who were randomized, received at least one intervention, and had VAS scores (a. average TMJ pain over the past week) measured at least once before and once after the intervention.
- In cases where VAS score data are missing, a diagnosis of the pattern and mechanism of missingness will first be performed. Based on this, an appropriate imputation method will be selected for Full Analysis Set (FAS) analysis.
- The difference in VAS score change between the two groups will be tested using an independent t-test. If there is a significant difference in baseline values, analysis of covariance (ANCOVA) will be conducted. To adjust for differences in other baseline variables, multiple regression analysis will be used.
- To examine time-by-treatment interaction effects on repeated VAS measurements, repeated measures ANOVA will be conducted.

② Secondary Outcome Measures

- For variables including VAS score, vertical mandibular opening, Jaw Functional Limitation Scale (JFLS-8), DC/TMD Chronic Pain Grade Scale (Version 2), Treatment Expectation Scale, and Patient Global Impression of Change (PGIC), either ANCOVA or multiple regression analysis adjusting for baseline variables will be used.
- In this case, the baseline score will be used as a covariate, and a per-protocol analysis without separate treatment of missing data will be performed.

2) Cost-Effectiveness Analysis

- Analysis period: The base analysis period is 10 weeks. If longer-term estimations are needed, long-term cost and effect projections will be modeled using approaches such as the Markov model.
- Model: Decision tree model
- Perspective: Both healthcare system and societal perspectives will be considered.
- Quality-Adjusted Life Years (QALYs): Quality of life derived from EQ-5D will be used as the primary outcome, calculated using the area under the curve method.
- Incremental Cost-Utility Ratio (ICUR): Calculated based on EQ-5D-5L results and cost data.

$$ICUR = \frac{\text{전체비용}_{CP\text{적용군}} - \text{전체비용}_{\text{일반치료군}}}{\text{질보정수명}_{CP\text{적용군}} - \text{질보정수명}_{\text{일반치료군}}}$$

- Incremental Cost-Effectiveness Ratio (ICER): Calculated based on individual effectiveness outcomes and cost data.

	$ICER = \frac{전체비용_{CP적용군} - 전체비용_{일반치료군}}{효과_{CP적용군} - 효과_{일반치료군}}$<br>- Base-case analysis: Conducted using representative parameter values (e.g., means) collected during the study.<br>- Sensitivity analysis:<br>- Deterministic sensitivity analysis will be conducted for all relevant parameters, and results will be presented using a tornado diagram.<br>- Probabilistic sensitivity analysis will also be conducted using distributions and representative values for all parameters.<br>3) Safety Outcome Measures<br>- Frequency, incidence rate, detailed listing, time of onset, severity, and causality with the investigational treatment will be presented. If necessary, results will be visualized using graphs.<br>- If statistical analysis is required, the appropriate test will be selected based on the nature and purpose of the variable, including: paired t-test, McNemar test, ANOVA, independent t-test, chi-square test, or Fisher's exact test.

임상시험 일정 (Clinical Trial Schedule)

기간period	스크리닝screening & 처치treatment				추적관찰 follow-up
주week	-1	0~5W±1		6W±1	10W±5
방문visit[1)]	스크리닝	1	2~11	12	13
동의서 취득<br>Obtain informed consent form	●				
인구학적 조사<br>Demographic survey	●				
병력 및 치료력 조사<br>Medical and treatment history	●	●	●	●	●
활력징후 측정<br>Vital signs	●	●	●	●	●
선정/제외기준 확인<br>Check inclusion/exclusion criteria	●				
X-Ray검사[2)]<br>X-Ray examination	●				
이학적 검진[3)]<br>Physical examination	●				
임신진단검사[4)]<br>Pregnancy test	●				
무작위 배정<br>Random assignment		●			
병용약물 확인<br>Check concomitant medications		●	●	●	●
이상반응 확인<br>Check for adverse events		●	●	●	●
TBT[5)] 또는 TENS[6)]<br>TBT or TENS		◇	◇	◇	
턱관절 장애 교육<br>Patient education		●			
턱관절 통증 VAS<br>TMJ pain VAS	●	●		●	●
수직 턱관절 개구도 확인<br>Check the vertical TMJ opening		●		●	●
JFLS-8<br>Jaw Functional Limitation Scale-8		●		●	●
만성통증 척도 2.0[7)]<br>DC/TMD The Graded Chronic Pain Scale (GCPS 2.0)		●		●	●
치료 기대 척도<br>Treatment Expectancy Scale		●		●	●
PGIC<br>Patient Global Impression of Change		●		●	●
효용측정[8)]<br>Utility measurement		●		●	●

**비용측정[9)]** Cost measurement		●		●	●
**순응도 확인[10)]** Check compliance			●	●	●
**방문일정 교육** Visit schedule information		●	●	●	

1) 방문1은 Screening으로부터 0-10일 이내에 행해져야 한다. Screening에서 선정제외 기준을 모두 충족하는 것을 확인한다면, 방문1이 동일한 날에 시행될 수 있다. 방문1은 Baseline 방문이며, 중재 시술 후 주 2회 방문한다. 방문허용일은 Baseline으로부터 각 주차별 해당하는 날짜±1일로 하며, 해당 날짜가 주말인 경우 가장 가까운 평일로 대체한다. 추적관찰은 Baseline으로부터 10주±5일의 허용일을 둔다. Screening과 방문1이 동일한 날인 경우 중복되는 검사나 설문은 1회만 시행한다. (Visit 1 must be conducted within 0–10 days from the screening. If the subject meets all inclusion and exclusion criteria during the screening, Visit 1 may be conducted on the same day. Visit 1 serves as the Baseline visit, after which the participant will visit twice per week for the intervention. Visit windows are defined as ±1 day from the scheduled date based on the Baseline date; if the scheduled date falls on a weekend, it may be rescheduled to the nearest weekday. Follow-up is scheduled at 10 weeks ±5 days from the Baseline. If Screening and Visit 1 occur on the same day, any overlapping tests or questionnaires will be conducted only once.)
2) T.M Joint View 및 Open Mouth View (T.M. Joint View and Open Mouth View will be conducted.)
3) 이학적 검진은 '측두하악장애 진단기준(DC/TMD): 평가도구(한국어)'의 Axis Ⅰ 의 'Examination: Pain-related Interview and Examiner Commands' 및 'DC/TMD Examination Form' 중 디스크 장애에 대한 내용을 제외하고 근육통, 근막통증, 관절통의 진단에 대한 내용을 발췌하여 수행한다. (Physical examination will follow the "Diagnostic Criteria for Temporomandibular Disorders (DC/TMD): Assessment Instruments (Korean Version)", using Axis I's "Examination: Pain-related Interview and Examiner Commands" and the "DC/TMD Examination Form" excluding items related to disc disorders. Only diagnostic procedures related to myalgia, myofascial pain, and arthralgia will be performed.)
4) 임신진단검사: 폐경이 되지 않은 여성 대상자는 Urine HCG를 시행하며 결과는 음성이어야 한다. 단, Screening에서 menstruation에는 visit1에 시행한다. (Pregnancy Test: Female participants who are not postmenopausal must undergo a urine hCG test, and the result must be negative. If menstruation is ongoing during screening, the test will be performed at Visit 1.)
5) ◇로 표기한 항목은 배정군에 따라 차등적으로 실시하며, TBT로 표시한 항목은 모두 시험군에 한해 실시한다. (Items marked with ◇ will be conducted differently depending on the assigned group. Items marked TBT will be performed only for the treatment group.)
6) ◇로 표기한 항목은 배정군에 따라 차등적으로 실시하며, TENS로 표시한 항목은 모두 대조군에 한해 실시한다. (Items marked with ◇ will be conducted differently depending on the assigned group. Items marked TENS will be performed only for the control group.)
7) 만성통증 척도 2.0은 '측두하악장애 진단기준(DC/TMD): 평가도구(한국어)'의 Axis Ⅱ 의 '만성통증 등급척도 2판'을 사용하여 수행한다. (The Chronic Pain Grade Scale 2.0 will be conducted using the "DC/TMD: Assessment Instruments (Korean Version)", specifically Axis II's "Graded Chronic Pain Scale Version 2.0".)
8) 효용측정은 EQ-5D, EQ-VAS를 사용하여 수행한다. (Utility measurement will be conducted using EQ-5D and EQ-VAS.)
9) 비용측정은 의료비용, 생산성손실비용의 조사를 위하여 별도 개발된 비용 조사지를 사용하여 조사한다. (Cost measurement will be conducted using a separately developed cost survey form to assess medical costs and productivity loss costs.)
10) Visit 2부터 Visit 12까지는 매 방문 시 대상자의 순응도를 평가할 때에는 예상되는 최대 중재횟수를 12로 나눈 값을 활용하여 계산한다. (From Visit 2 to Visit 12, participant compliance will be evaluated at each visit. Compliance will be calculated by dividing the number of completed sessions by the expected total number of interventions (12).)

1. 임상시험의 명칭 (Name of clinical trial)

[국문] 턱관절 장애에 대한 TBT의 유효성, 안전성, 경제성 평가를 위한 무작위 대조군 임상시험
[Englush] A Randomized Controlled Clinical Trial to Evaluate the Effectiveness, Safety, and Economics of TBT for Temporomandibular Disorder

2. 임상시험 실시기관, 임상시험 책임자/담당자 (Clinical trial implementation institution, clinical trial director/person in charge)

2.1 주관기관 (Host organization)

대전대학교 대전한방병원. 대전광역시 서구 대덕대로 176번길 75
전화번호: 042-470-9131
Daejeon Korean Medicine Hospital of Daejeon University. 75, Daedeok-daero 176beon-gil, Seo-gu, Daejeon.
Phone: 042-470-9131

2.2 실시기관 (Implementing organization)

대전대학교 대전한방병원. 대전광역시 서구 대덕대로 176번길 75
전화번호: 042-470-9131
Daejeon Korean Medicine Hospital of Daejeon University. 75, Daedeok-daero 176beon-gil, Seo-gu, Daejeon.
Phone: 042-470-9131

2.2.1 임상시험 책임자 (Clinical trial director (Principal Intestigator))

유 호 룡 대전대학교 한의학과 한방내과 교수
HoRyong Yoo, Professor of Internal Korean Medicine,
Daejeon University College of Korean Medicine

2.2.2 임상시험 공동 시험자 (Clinical trial co-investigator)

[별첨 1] 임상시험 담당자 명단 참조
[Appendix 1] See list of clinical trial personnel

2.2.3 연구의사 (Research clinician)

[별첨 1] 임상시험 담당자 명단 참조
[Appendix 1] See list of clinical trial personnel

2.2.4 통계담당자 (Statistician)

[별첨 1] 임상시험 담당자 명단 참조
[Appendix 1] See list of clinical trial personnel

2.2.5 임상시험 코디네이터 (Clinical Research Coordinator)

[별첨 1] 임상시험 담당자 명단 참조
[Appendix 1] See list of clinical trial personnel

2.3 대상자 모집 방법 (Method of recruting subjects)

대상자 확보를 위하여 실시기관 내외 게시판 등에 홍보물을 부착한다.
In order to secure subjects, promotional materials are attached to bulletin boards inside and outside the implementing agency.

3. 임상시험 의뢰자 (Clinical trial client)

유 호 룡 대전대학교 한의학과 한방내과 교수
대전대학교 대전한방병원. 대전광역시 서구 대덕대로 176번길 75
전화번호: 042-470-9131
HoRyong Yoo, Professor of Internal Korean Medicine
Daejeon Korean Medicine Hospital of Daejeon University. 75, Daedeok-daero 176beon-gil, Seo-gu, Daejeon.
Phone: 042-470-9131

4. 임상시험 모니터 (Clinical trial monitor)

임상시험책임자는 연구 개시 이후 최초 2개월간 1개월마다 이상반응, 연구진행, 상황, 결과 등에 대하여 파악하며 진행 상황을 확인한다. 이후 2개월마다 진행 상황을 확인한다.
The clinical trial director will check the progress of the study by identifying adverse reactions, study progress, situation, results, etc. every month for the first two months after the start of the study. Thereafter, the progress will be checked every two months.

5. 임상시험의 목적 및 배경 (Purpose and background of the clinical trial)

5.1 목적 (Purpose)

• 턱관절 장애에 대한 TBT의 유효성, 안전성, 경제성을 평가한다.
• To Evaluate the Effectiveness, Safety, and Economics of TBT for Temporomandibular Disorder

5.2 배경 (Background)

5.2.1 연구배경 (Research background)

- 턱관절 장애는 저작근과 측두하학관절 기능에 영향을 미치는 통증 질환의 그룹이다.
- 턱관절 장애로 인한 대표적인 증상으로는 구강안면통증, 근육 결림, 뻣뻣함, 제한된 목소리 및 하악의 움직임 등이 있다.
- 턱관절 장애는 또한 1차 두통 장애 및 만성 두통과도 관련이 있으며, 환자의 얼굴 미학 뿐 아니라 신체적, 심리적 건강에도 영향을 미친다.
- 턱관절 장애는 인구 집단의 약 5 ~ 12% 정도에 영향을 미치는 것으로 알려져 있다. 일부 역학 연구에 따르면 인구 집단의 약 50~75% 정도가 어느 정도 턱관절 장애의 증상을 가지고 있다.
- 국민건강보험공단 건강보험 진료데이터에 따르면 2019년 턱관절 장애 환자는 41만 명에 이르고, 2015~2019년 연평균 증가율은 4.0%이다.
- 물리치료, 약물치료, 교합 조절, 심리적 접근 등 다양하지만, 장기적인 효과는 제한적이며, 일부 약물치료의 경우 부작용이 있으며, 비침습적 치료법의 효과는 개인차가 크다.
- DC/TMD(Diagnostic Criteria for Temporomandibular Disorders)는 턱관절장애(TMD)를 진단하려고 만든 표준화된 기준이며, 생체-심리-사회적 관점에서 턱관절과 주변 근육의 통증과 기능 장애를 평가함. 1992년 RDC/TMD로 시작되었으며, DC/TMD로 개정되었음. 두 가지 축으로 나뉘는데, Axis I은 근육 장애, 관절 장애와 같은 생물학적 진단을 다루고, Axis II는 통증 강도, 기능 장애, 심리 상태와 같은 심리사회적 요인을 평가함. 이를 바탕으로 환자의 증상을 체계적으로 분석 가능하고, 치료 계획도 더 정확히 세울 수 있음. 지금은 전 세계적으로 많이 쓰이고 있는 턱관절장애 진단의 표준에 해당함.
- DC/TMD 진단 기준에 따르면 턱관절 장애는 다음과 같은 3가지 주요 진단군으로 분류될 수 있음: (1) 근육 장애, (2) 관절 디스크 장애, (3) 관절 질환. 3가지 주요 진단군 중 (1) 근육 장애를 반드시 가지고 있는 턱관절 장애 환자를 대상으로 본 연구를 수행하고자 함. 연구 대상자는 (2) 관절 디스크 장애, (3) 관절 질환을 동반할 수도, 동반하지 않을 수도 있음.

  (1) 근육 장애(Muscle Disorders)
  특징: 저작근 부위의 통증 및 기능 장애.
  분류 기준:
  근막통(Masticatory Muscle Pain): 촉진 또는 움직임으로 유발되는 저작근 부위의 국소 통증.
  근막통과 연관된 두통(Headache Attributed to TMD): 근막통에 의해 두통이 유발되거나 악화됨.

  (2) 관절 디스크 장애(Disk Displacement Disorders)
  특징: 턱관절 내 디스크의 위치 이상으로 인해 관절 움직임 및 기능에 영향을 미침.
  분류 기준:
  디스크 변위(Disk Displacement)
  복원성 디스크 변위(Disk Displacement with Reduction): 개구 시 디스크 위치가 정상으로 복원되며 클릭음이 들림.
  비복원성 디스크 변위(Disk Displacement without Reduction): 디스크 위치가 복원되지 않아 관절 운동 제한이 동반됨.
  제한성 비복원성: 개구량 제한이 있음.
  비제한성 비복원성: 개구량 제한은 없음.

(3) 관절 질환(Joint Disorders)
특징: 관절 자체의 구조적 또는 염증성 변화로 인한 장애.
분류 기준:
관절염(Arthralgia): 턱관절의 통증 및 압통.
관절염증(Osteoarthritis): 관절 구조의 염증성 변화 및 퇴행성 손상.
관절파괴(Osteoarthrosis): 관절의 퇴행성 변화가 있지만 염증 증상은 없음.

- Temporomandibular disorders (TMD) refer to a group of painful conditions that affect the masticatory muscles and the temporomandibular joint (TMJ).
- Common symptoms of TMD include orofacial pain, muscle tightness, stiffness, limited jaw mobility, and restricted mandibular movement.
- TMD is also associated with primary and chronic headache disorders, and it can impact not only facial aesthetics but also the patient's physical and psychological health.
- It is estimated that approximately 5–12% of the general population is affected by TMD. Some epidemiological studies suggest that 50–75% of the population experience some symptoms related to TMD.
- According to data from the National Health Insurance Service of Korea, 410,000 patients were treated for TMD in 2019, and the average annual increase in patient numbers between 2015 and 2019 was 4.0%.
- While various treatments such as physical therapy, pharmacotherapy, occlusal adjustments, and psychological interventions are used, their long-term efficacy is limited. Some medications can cause side effects, and the effectiveness of non-invasive treatments varies widely between individuals.
- The Diagnostic Criteria for Temporomandibular Disorders (DC/TMD) is a standardized diagnostic tool for TMD that evaluates pain and dysfunction of the TMJ and surrounding muscles from a biopsychosocial perspective.
- It was first introduced in 1992 as the RDC/TMD and was later revised into the DC/TMD.
- It consists of two axes: Axis I focuses on biological diagnoses such as muscle and joint disorders, while Axis II assesses psychosocial factors such as pain intensity, functional limitation, and psychological status.
- This system allows for a structured analysis of patient symptoms and facilitates more accurate treatment planning.
- It is now widely used as the international standard for diagnosing TMD.
- According to the DC/TMD diagnostic framework, TMD can be classified into the following three major diagnostic groups: (1) Muscle Disorders, (2) Disk Displacement Disorders, (3) Joint Disorders
- This study aims to include TMD patients who must present with (1) Muscle Disorders. They may or may not also have (2) Disk Displacement Disorders or (3) Joint Disorders.

(1) Muscle Disorders
- Characteristics: Pain and functional impairment in the masticatory muscles
- Subtypes:

Myalgia: Localized pain in the masticatory muscles provoked by palpation or movement
Headache Attributed to TMD: Headache that is induced or aggravated by myalgia

(2) Disk Displacement Disorders
- Characteristics: Impaired movement and function due to abnormal positioning of the articular disk within the TMJ
- Subtypes:
- Disk Displacement with Reduction: The displaced disk returns to its normal position during mouth opening, often accompanied by clicking sounds
- Disk Displacement without Reduction: The disk does not return to its normal position, resulting in restricted joint movement
- With limited opening: Reduced range of jaw opening
- Without limited opening: No reduction in jaw opening despite displacement

(3) Joint Disorders
- Characteristics: Disorders caused by structural or inflammatory changes within the joint
- Subtypes:
  Arthralgia: Pain and tenderness localized to the TMJ
  Osteoarthritis: Inflammatory and degenerative changes in joint structures
  Osteoarthrosis: Degenerative joint changes without inflammatory symptoms

5.2.2 국내·외 기술개발현황 및 본 연구의 필요성 (Domestic and International Technology Development Status and the Necessity of This Study)

- 2015~2019년 '턱관절장애(K07.6)' 건강보험 진료 현황을 살펴보면 5년간 총진료 인원은 2015년 35만 명에서 2019년 41만 명으로 17.1%(6만명)가 증가, 연평균 증가율은 4.0%로 나타남.
- 2010~2018년 건강보험심사평가원(HIRA-NPS) 자료 분석 결과 20대 환자가 27%로 가장 많고, 여성 환자가 59%로 남성보다 1.44배 많음. 치과 진료 비율이 2010년 57.7%에서 2018년 77.2%로 증가, 의과 진료는 42.6%에서 21.0%로 감소, 한의과 진료는 6.7%에서 8.3%로 증가함.
- 약물치료로는 비스테로이드성 소염진통제(NSAIDs)와 근육이완제를 주로 사용, 아편유사제, 항불안제, 항우울제 처방은 감소함.
- 비약물치료로는 물리치료와 침치료 비율 증가(침치료: 2010년 6.6%에서 2018년 8.0%).
- 여성 환자가 남성보다 외래 방문율, NSAIDs, 근육이완제, 물리치료 사용률이 높음.
- 2021년 턱관절 장애에 대한 한의표준임상진료지침 권고안을 보면 R13~R14 부분이 비어있는데 각각 구강내 균형장치와 매선에 대한 임상질문을 설정하였으나 관련 근거가 부족하여 근거수준과 권고등급을 부여하지 못하였음. 이에 턱관절 장애에서 구강내 균형장치에 대한 근거 확보가 필요함.
- According to the National Health Insurance data on Temporomandibular Disorders (K07.6) from 2015 to 2019, the total number of patients increased from 350,000 in 2015 to 410,000 in 2019, representing a 17.1% increase (60,000 individuals) over five years, with an average annual growth rate of 4.0%.

- An analysis of HIRA-NPS data from 2010 to 2018 showed that patients in their 20s accounted for the largest proportion (27%), and female patients (59%) were 1.44 times

more numerous than males.

- The proportion of dental visits increased from 57.7% in 2010 to 77.2% in 2018, while medical (Western medicine) visits decreased from 42.6% to 21.0%, and Korean medicine visits increased from 6.7% to 8.3%.
- In terms of pharmacotherapy, non-steroidal anti-inflammatory drugs (NSAIDs) and muscle relaxants were most commonly used, while prescriptions for opioids, anxiolytics, and antidepressants decreased.
- For non-pharmacological treatments, both physical therapy and acupuncture usage increased (acupuncture: from 6.6% in 2010 to 8.0% in 2018).
- Female patients had higher rates of outpatient visits, and greater use of NSAIDs, muscle relaxants, and physical therapy compared to male patients.
- According to the 2021 Korean Medicine Clinical Practice Guideline (CPG) for TMD, items R13 to R14, which were meant to address intraoral balancing appliances and thread embedding therapy, lacked sufficient evidence. As a result, no evidence level or recommendation grade could be assigned. Therefore, there is an urgent need to establish scientific evidence regarding the efficacy of intraoral balancing appliances in the treatment of temporomandibular disorders.

6. 대상질환과 증상 (Target diseases and symptoms)

- 턱관절장애로 인한 통증과 기능장애
- Pain and functional disorders due to temporomandibular joint disorder

7. 중재방법 (Intervention methods)

7.1 TBT(기능적 뇌척주요법) 방법 (TBT(FCST) method)

7.1.1 TBT 치료 방법 및 설정의 합리성 (Rationality of TBT Treatment Method and Settings)

- 한의사 중 1년 이상의 해당 분야 임상경험이 있는자가 시행한다.
- TBT 치료는 다음의 3단계 과정을 통해 진행된다.
- The procedure must be performed by a licensed Korean medicine doctor with at least one year of clinical experience in the relevant field.
- TBT (Temporomandibular Balancing Therapy) is conducted in the following three-step process:

1. 턱관절의 균형점 찾기 및 편차 수정

- 맞춤형 교정을 위하여 턱관절의 좌우, 전후, 상하(X-Y-Z축)의 균형을 만족시키고, 경추가 최대한 정렬되는 균형점을 찾아 상악에 대한 하악 관절의 위치를 고정시키며, 이를 위해 측정바*(진바이오텍, 대한민국)*를 사용한다.

1. Identifying the TMJ Balance Point and Correcting Deviations

- To ensure customized correction, the balance of the temporomandibular joint along the

X (left-right), Y (front-back), and Z (up-down) axes is assessed. The goal is to identify a balance point where the cervical spine is optimally aligned, and to fix the mandibular joint in a stable position relative to the maxilla. For this, a measuring bars (Jin Biotech, Republic of Korea) are used.

2. 하악 관절 임시 고정
- 하악 관절의 위치를 일시적으로 고정시키기 위해 부가 중합형 폴리비닐실리콘 인상제(제품명: Exafine Putty, GC Dental products cor., 일본*)를 사용한다.
- 경부 촉진, 측경부 근긴장도 검사, 경추 회전 검사를 통해 좌우 긴장도를 확인하고, 경추 회전 정도가 같아지는 높이를 찾아 측정바와 측정 페이퍼를 양쪽 어금니로 물게 한 다음 두가지 인상제를 섞어서 치아 모양을 본떠서 굳힌 다음 약 2분간 착용한다.

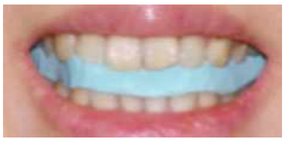

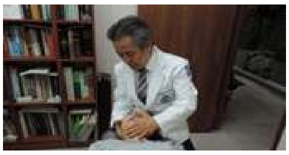

2. Temporary Fixation of the Mandibular Joint
- To temporarily fix the position of the mandibular joint, addition-curing vinyl polysiloxane impression material (Exafine Putty, GC Dental Products Corp., Japan) is used.
- Palpation of the neck, lateral neck muscle tone assessment, and cervical rotation tests are performed to assess muscle tone on both sides.
- The appropriate vertical height is determined when left and right cervical rotation become equal.
- The participant bites the measuring bar and paper with both molars, after which two types of impression material are mixed, molded to the teeth, and held in place for approximately 2 minutes.

3. 근막 이완
- 근막 이완 추나 치료를 진행해 손에서 느껴지는 근막의 이완 반응을 확인한다.
- 턱 스트레칭을 5회 시행한다.

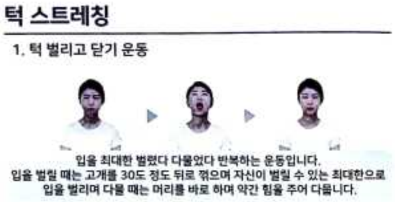

3. Myofascial Release
- Myofascial release manipulation (Chuna therapy) is performed, during which the practitioner confirms the softening of fascial tension through palpation.
- Jaw stretching is performed five times.

- TMJ 주변에 분포된 9개의 뇌신경 중 5번 뇌신경은 상부경추 지배근육과 연관되어있어, 턱관절의 불균형으로 인해 부정적인 시그널이 발생하면 상부경추와 연관된 근육군이 신장 또는 수축되며 상부 경추(C1, C2)의 아탈구가 진행된다. TBT 치료의 1단계를 통해 턱관절이 균형위치에 놓이게 되면 부정적 시그널이 꺼지면서 아탈구를 유발시키고 있던 상부 경추 주변 근육이 이완상태에 놓이게 되며, 좀 더 안전하고 정확하게 교정이 가능해진다.

- 추나 시행 전 근육의 촉진 및 상부 경추 회전 제한 확인, 측경부 긴장 및 압통 확인 등 경추 진단검사를 실시한다. 이는 경추의 정렬을 확인하여 보다 가벼운 동작으로 안전하고 손쉽게 경추교정이 이루어질 수 있도록 한다.
- Among the nine cranial nerves that innervate the TMJ area, the trigeminal nerve (CN V) is particularly associated with muscles governed by the upper cervical spine. If imbalance in the TMJ generates abnormal signals, it can lead to elongation or contraction of associated muscles, contributing to subluxation of the upper cervical spine (C1, C2). When the TMJ is repositioned to its balanced position in Step 1 of TBT, the abnormal signals are reduced, allowing the surrounding upper cervical muscles to relax. This enables safer and more precise spinal alignment.
- Before performing Chuna therapy, cervical assessments such as muscle palpation, upper cervical rotation limitation, lateral neck tension, and tenderness evaluation are conducted. These assessments help identify cervical misalignment, ensuring that correction can be performed more safely and with lighter movements.

* 측정바 (Measuring bar)
- 1.6mm, 실리콘 재질로 제작되었다.
- 1.6mm, silicone-based bars

* Exafine Putty(제조사(manufactured by): GC, 원산지(country): 일본(Japan))
- Vinyl polysiloxane impression material의 정밀인상재로서 인상 채득용으로 사용된다.
- A vinyl polysiloxane impression material used for precision molding of dental impressions.

7.1.2 TBT의 고려사항 (Considerations for TBT)

- 치료 과정 중 턱관절은 이상적이고 안정화된 균형점'에 도달하기까지 '임시적 균형상태'와 '임시적 불균형상태'를 오가며 반복되는 변화과정을 거치게 된다. 이때 발생하는 '임시적 불균형상태'를 '편차'라고 한다. 편차가 발생하게 되면, 평소 가지고 있던 불편한 증상이 다시 나타나거나 더욱 불편해지는 양상으로 나타날 수 있다. 이때 이를 완화시키기 위해 새롭게 변화된 임시적 균형점을 찾는 편차 수정이 필요하다. 편차 수정은 대상자의 상태에 따라 매 Visit마다 1~3회 시행하도록 한다.
- During the course of treatment, the temporomandibular joint (TMJ) undergoes repeated transitional changes, alternating between temporary balanced states and temporary unbalanced states before reaching an ideal and stabilized balance point. These temporary unbalanced states are referred to as "deviations". When a deviation occurs, previously existing discomfort may reappear or worsen temporarily. In such cases, it is necessary to identify a new, temporarily balanced point to alleviate symptoms — this process is referred to as deviation correction. Deviation correction should be performed 1 to 3 times per visit, depending on the participant’s condition.

7.2 TENS(경피적전기신경자극치료, Transcutaneous Electrical Nerve Stimulation)

7.2.1 TENS 수행방법 (TENS Procedure)

- 대조군에 대해 실시한다.
- TENS is performed on the control group.
- 1년 이상의 임상경력을 가진 한의사가 시행한다.
- 처치 부위 : 환측 교근 부위
- 시술 방법 : TENS 치료는 환측(통증이 양쪽에 있을 시 양쪽)의 교근 부위에 실리콘 패드 1쌍을 부착하여 3Hz의 주파수로 시행하되, 자극 강도는 자극 감각은 있되 과도한 근육 수축이나 통증은 느껴지지 않는 강도로 15분간 치료한다.
- 치료 횟수 : 주당 2회, 6주간 총 12회
- The procedure is carried out by a Korean medicine doctor with at least one year of clinical experience.
- Treatment area: The masseter muscle on the affected side
- Procedure: A pair of silicone pads is attached to the masseter area on the affected side (or both sides if pain is bilateral). TENS is applied at a frequency of 3 Hz, with an intensity that produces a sensory stimulation but does not induce excessive muscle contraction or pain. Each session lasts 15 minutes.
- Treatment frequency: Twice per week for 6 weeks, totaling 12 sessions.

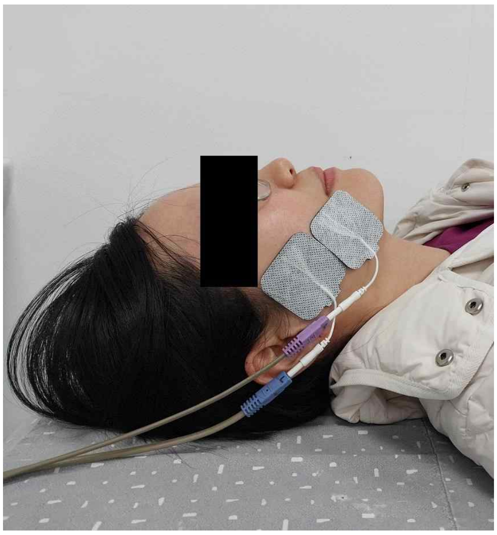

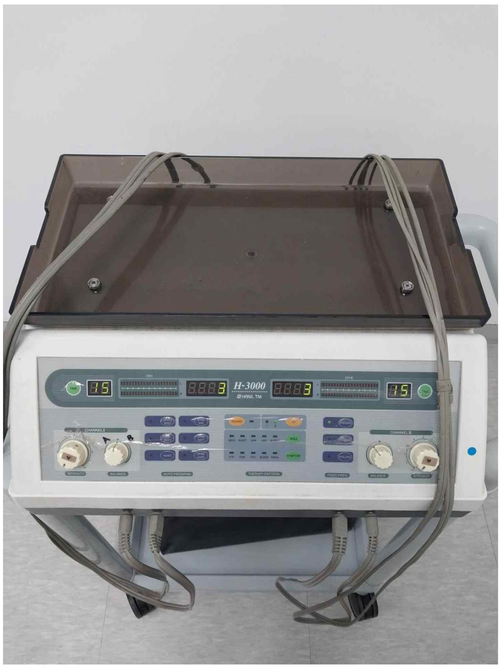

* 저주파 자극기 / H-3000-P (제조사: (주)한일티엠, 제조국: 한국)

* Device used: Low-frequency stimulator / H-3000-P (Manufacturer: Hanil TM Co., Ltd., Republic of Korea)

7.2.2 TENS의 고려사항 (Considerations for TENS)

턱관절 장애에 가장 일반적으로 시행되는 치료로는 자가관리, 행동요법, 물리치료, 교합스플린트, 약물 등이 사용되는데, 그 중 물리치료가 폭 넓게 사용된다. 물리치료는 통증을 경감시키고 ROM을 증가시키기 위해 시행되는데, 그 중 전기치료로는 경피전기신경자극(TENS), 초음파, 레이저 등이 사용되며, 염증감소, 국소 혈류 증가, 근이완 촉진을 목표로 사용된다.[1]

TENS는 여러 통증 상태에 대해 경제적이고 안전하며 비침습적 치료 방식인데, 전극을 피부 표면에 적용하여 과활성화된 근육을 이완시키고 통증 완화를 유도한다. 전극은 자체 접착형 실리콘 패드 도자를 적용할 수도 있고, 흡입형 도자를 사용할 수도 있다. 전극 부착 위치는 통증처와 동일한 피부분절, 근분절 및 근막 통증 유발점 내에서 통증의 시작점이나 가장 통증이 심한 부위에 배치할 수 있으며 통증 발생 및 유지와 관련된 말초 신경 경로에 이를 배치할 수도 있다.

턱관절 장애에 적용되는 TENS는 만성 통증을 조절 하고 저작근을 이완시키는 것을 목표로 하는데, 주파수는 다양하게 사용할 수 있다. 고주파(50–150Hz) 장치는 강도가 낮고 장기적인 통증을 완화하는 것을 목표로 하며, 저주파(<10Hz) 장치는 일반적으로 근육 이완을 목표로 한다. 장치의 강도는 환자의 민감도에 따라 조절하여 근육의 과도한 수축이나 감각 이상을 피하도록 해야한다.[2] 적용 시간은 특별히 권고된 바는 없으나 여러 연구에서 대부분 15~20분간 시행하였다.[3-6]

위의 내용을 토대로 근육 이완을 통한 통증 완화를 목표로 TENS 저주파 자극(<10Hz)을 선택하되 TENS 기계 셋팅값이 3Hz로 고정되어 있어, 3Hz로 하여 환측에(양쪽 모두 통증 있을 시 양측에) 15분간 시행하는 것으로 하였다. 도자는 시술 자리에 자국이 남지 않도록 실리콘 패드 도자를 선택하였다.

The most common treatments for temporomandibular disorders (TMD) include self-care, behavioral therapy, physical therapy, occlusal splints, and medications. Among these, physical therapy is widely used to relieve pain and improve range of motion (ROM). Among the electrical modalities, transcutaneous electrical nerve stimulation (TENS), ultrasound, and laser therapy are commonly employed, with goals such as reducing inflammation, increasing local blood flow, and promoting muscle relaxation.[1]

TENS is a cost-effective, safe, and non-invasive method for treating various pain conditions. It works by placing electrodes on the skin surface to relax hyperactive muscles and alleviate pain. Electrodes can be self-adhesive silicone pads or suction-type electrodes. The placement of electrodes can be at:

- The same cutaneous dermatomes or myotomes as the pain site
- The initial site of pain onset or most painful area within a myofascial trigger zone
- Peripheral nerve pathways associated with pain initiation or maintenance

In TMD, TENS is used to manage chronic pain and relax masticatory muscles.

Various frequencies may be used:

- High-frequency TENS (50–150 Hz): generally applied at low intensity for long-term pain relief
- Low-frequency TENS (<10 Hz): typically aimed at muscle relaxation

The intensity of stimulation should be adjusted to each patient's sensitivity to avoid excessive muscle contraction or paresthesia.[2]

Although there are no strict guidelines for duration, most studies apply TENS for 15–20 minutes.[3-6]

Based on the above, low-frequency TENS (<10 Hz) was selected for this study, targeting muscle relaxation and pain relief. As the TENS device used in this study has a fixed frequency setting of 3 Hz, treatment is applied at 3 Hz for 15 minutes on the affected side (or both sides if pain is bilateral). Silicone pad electrodes are used to avoid leaving marks at the treatment site.

TENS 금기사항
1) 인공심장박동기를 한 경우와 경동맥부위
2) 임신 중인 여성의 자궁 위나 복부 및 허리 부위

Contraindications for TENS
1) Patients with a cardiac pacemaker or application over the carotid sinus region
2) Application over the uterus, abdomen, or lower back in pregnant women

1. Murphy MK, MacBarb RF, Wong ME, Athanasiou KA. Temporomandibular disorders: a review of etiology, clinical management, and tissue engineering strategies. The International Journal of Oral & Maxillofacial Implants. 2013 Nov-Dec;28(6):e393-414.
2. Awan, K. H., & Patil, S. (2015). The role of transcutaneous electrical nerve stimulation in the management of temporomandibular joint disorder. J Contemp Dent Pract, 16(12), 984-6.
3. Chellappa, D., & Thirupathy, M. (2020). Comparative efficacy of low-Level laser and TENS in the symptomatic relief of temporomandibular joint disorders: A randomized clinical trial. Indian Journal of Dental Research, 31(1), 42-47.
4. Mishra, S., Bajoria, A. A., Sangamesh, N. C., Swain, A. K., Sahoo, S. K., & Mohapatra, A. (2024). Low-Level Laser and TENS Therapy Assessment for the Treatment of Temporomandibular Joint Disorder. Journal of Pharmacy and Bioallied Sciences, 16(Suppl 3), S2179-S2181.
5. Kirupa, K., Divya Mary, S., Vaishnavi, G., Nisha, R. N., Mercy, J. R., & Jaiganesh, G. (2019). A comparative study of ultrasound therapy and transcutaneous electrical nerve stimulation in reducing pain for temporomandibular joint disorder. Drug Invent Today, 12(3), 515-7.
6. Anupriya, C., Nahar, P., Singh, M. P., Bhuvaneshwari, S., Goel, S., & Mathur, H. (2023). TENS therapy or low-level laser therapy? In the management of morbidities associated with temporomandibular joint disorders: A comparative study. Journal of Indian Academy of Oral Medicine and Radiology, 35(2), 187-190.

8. 임상시험 기간 (Clinical trial duration)

- 본 임상시험의 기간은 본 임상시험계획서에 대한 기관생명윤리위원회(IRB) 승인일로부터 12개월로 한다. 단, 피험자 등록 속도에 따라 연장될 수 있다.
- This study will last 12 months from the date this research plan was approved by the institutional review board (IRB). However, depending on the subject registration, it may be shortened or extended.

9. 대상자 선정기준, 제외기준, 목표한 대상자 수 및 그 근거, 배정방법

9.1 선정기준 (Inclusion criteria)

1) 만 19세 이상 70세 이하의 남녀
2) DC/TMD 기준에 따라 턱관절 편측 또는 양측에 3개월 이상 지속되는 간헐적인 통증을 호소하는 자
3) 턱관절 통증을 호소하는 부위(양측 통증 환자의 경우 통증이 심한 방향 기준)의 통증이 지난 1주일간 평균적으로 VAS 40mm 이상인 경우
4) 턱의 움직임, 기능 및 주변 기능에 의해 발생하는 턱관절 및 주변부(관자놀이, 귓속, 귀 앞)의 국소적인 통증이 있는 경우
5) 검사자가 촉진을 통해 관자근, 깨물근, 또는 하악 관절의 관절 융기 외측극 부위에서 통증을 확인하여 근육통, 근막통증 또는 관절통 중 하나의 타입에 해당하는 경우; 또는 턱의 움직임 시 발현되거나 악화되는 두통이 측두근 부위에 위치하며 검사 시 재현 가능한 경우

1) Men and women aged 19 to 70 years.
2) Individuals who report intermittent pain in the temporomandibular joint (unilateral or bilateral) lasting for more than 3 months based on the DC/TMD criteria.
3) Individuals whose pain in the temporomandibular joint (based on the side with the most severe pain for patients with bilateral pain) has an average Visual Analog Scale (VAS) score of 40 mm or higher over the past week.
4) Individuals with localized pain in the temporomandibular joint and surrounding areas (temple, inside the ear, or in front of the ear) associated with jaw movement, function, or related activities.
5) Individuals whose pain is confirmed by the examiner through palpation in the temporalis muscle, masseter muscle, or the lateral pole of the mandibular joint's articular prominence, corresponding to one of the following types: myalgia, myofascial pain, or arthralgia; or individuals with headaches located in the temporalis area that are reproducible during examination and are triggered or worsened by jaw movement.

9.2 제외기준 (Exclusion criteria)

1) 양악수술 등 턱관절 관련 수술을 받은 자
2) 치료 효과나 결과의 해석을 방해할 수 있는 다발성 통증 질환(예: 류마티스 관절염), 신경계 질환(예: 뇌종양, 뇌졸중, 삼차신경통 등)이 있는 경우
3) 현재 스테로이드제제, 면역억제제, 정신질환 약물 또는 연구 결과에 영향을 줄 수 있는 기타 약물을 복용하고 있는 경우
4) 1개월 이내에 비스테로이드성 소염제 등의 통증에 영향을 줄 수 있는 약물 복용을 시작 또는 중단한 경우
5) 기타 의사의 판단 하에 본 연구 참여가 적합하지 않은 자 (신체 내 전기 기기, pacemaker 등을 시술 받은 자, 관절 침범 양상을 확인하였을 때 류마티스 관절염의 양상이 뚜렷하게 나타나는

환자 등)
6) 임산부 또는 수유부
1) Individuals who have undergone temporomandibular joint-related surgeries, such as orthognathic surgery.
2) Individuals with multiple pain disorders (e.g., rheumatoid arthritis) or neurological conditions (e.g., brain tumor, stroke, trigeminal neuralgia) that could interfere with the interpretation of treatment effects or outcomes.
3) Individuals currently taking steroids, immunosuppressants, psychiatric medications, or other drugs that could affect study results.
4) Individuals who have started or discontinued medications affecting pain, such as nonsteroidal anti-inflammatory drugs, within the past month.
5) Individuals deemed unsuitable for participation by the investigator (e.g., those with implanted electrical devices or pacemakers, or those showing clear signs of rheumatoid arthritis upon joint involvement assessment).
6) Pregnant or lactating women.

9.3 목표한 대상자 수 및 그 근거 (Targeted number of subjects and its basis)

9.3.1 무작위배정 시험대상자 수 (Number of randomized trial subjects)

- 총 30명(시험군 15명, 대조군 15명)
- total (N = 30) (Experimental group (N = 15), control group (N = 15))

	시험군 Experimental group	대조군 Control group	총 대상자수 Total subjects
유효성 평가 대상자 수 Number of subjects for effectiveness evaluation	15	15	30
탈락률, 순응도를 고려한 대상자수 Number of subjects considering dropout rate and compliance (Drop-out: 20%)	12	12	24

9.3.2 산출근거 (Basis of calculation)

1) 본 임상시험의 가설은 다음과 같다.
- H0(귀무가설): μt = μc vs. H1(대립가설): μt ≠ μc - μt : 치료군(TBT)의 베이스라인(baseline) 대비 6주 시점의 VAS (mm) 평균 변화값
- μc : 대조군의 베이스라인(baseline) 대비 6주 시점의 VAS (mm) 평균 변화값
- 평가변수의 통계적 가설검정: 양측 검정 - 유의수준(α): 0.05 - 제2종 오류(β): 0.1, 검정력(1-

β): 90% - 시험군과 대조군의 비율은 1:1로 동일하게 한다.

2) 본 연구와 연구 디자인, 처치의 기간, 방법 및 횟수, 평가변수 등이 가장 유사한 선행연구를 참고하여 계산하였다.
   - 해당 논문에서는 치료군 15명, 대조군 15명으로 총 30명의 대상자를 모집하였다. 처치 기간은 10주였으며, 총 처치 횟수는 12회였다. 1차 유효성 평가변수는 최대 개구도(maximum mouth opening (MMO))를 사용하였으며, VAS는 2차 유효성 평가변수로 사용되었다. 치료군과 대조군의 VAS(평균(표준편차))는 치료 전 각각 5.60(0.91), 5.40(1.06)이었으며, 치료 후 각각 1.67(0.62), 4.20(0.78)로 나타났으며, 대조군 대비 치료군의 효과 차이(Mean Difference)는 –2.53이고, 합동표준편차(Pooled SD)는 0.96으로 나타났다.
3) 효과 차이는 –2, 표준편차는 1.5로 보수적인 값을 설정하여 대조군 대비 치료군의 효과를 확인하기 위한 시험대상자 수를 산출하는 경우 결과는 아래와 같이 군당 약 12명이 필요한 것으로 나타난다. 중도탈락률 20%를 고려하면 군당 15명씩 총 30명의 임상시험 대상자가 필요한 것으로 나타난다.
   산출 공식: $\left\{\frac{2\left(z_{1-\frac{\alpha}{2}}+z_{\beta}\right)^{2}*\sigma^{2}}{|\mu_{T}-\mu_{c}|}\right\}=\left\{\frac{2(1.96+1.28)^{2}*1.5^{2}}{(-2)^{2}}\right\}=11.81\approx 12$
   중도탈락률 20%를 고려: 11.81/0.8=14.76, 약 15명, 총 30명.
4) 참고 논문: Rezaie K, Amiri A, Ebrahimi Takamjani E, Shirani G, Salehi S, Alizadeh L. The Efficacy of Neck and Temporomandibular Joint (TMJ) Manual Therapy in Comparison With a Multimodal Approach in the Patients with TMJ Dysfunction: A Blinded Randomized Controlled Trial. Med J Islam Repub Iran. 2022 May 4;36:45. doi: 10.47176/mjiri.36.45. PMID: 36128309; PMCID: PMC9448471.

1) The hypothesis of this clinical trial is as follows:
   - H0 (Null Hypothesis): μt = μc vs. H1 (Alternative Hypothesis): μt ≠ μc
   - μt: Mean change in VAS (mm) at 6 weeks compared to baseline in the treatment group (TBT)
   - μc: Mean change in VAS (mm) at 6 weeks compared to baseline in the control group
   - Statistical hypothesis testing for the outcome variable: Two-sided test
   - Significance level (α): 0.05
   - Type II error (β): 0.1, Power (1-β): 90%
   - The ratio of participants in the treatment and control groups is set to 1:1.
2) The sample size was calculated with reference to a prior study that most closely resembles the present study in terms of study design, duration, treatment method and frequency, and outcome variables.
   - In that study, a total of 30 participants were recruited (15 in the treatment group and 15 in the control group). The treatment period was 10 weeks, with a total of 12 treatment sessions. The primary efficacy outcome was pain intensity measured by VAS.
   - The mean (standard deviation) VAS scores before treatment were 5.60 (0.91) for the treatment group and 5.40 (1.06) for the control group. After treatment, the scores were 1.67 (0.62) and 4.20 (0.78), respectively.
   - The mean difference in effect between the treatment and control groups was –2.53, and the pooled standard deviation was 0.96.
3) A conservative effect size of –2 and standard deviation of 1.5 were used to calculate the required number of participants to confirm the treatment effect compared to the control group. The result showed that approximately 12 participants per group are needed. Considering a 20% dropout rate, a total of 30 participants (15 per group) are required.
   - Calculation formula:

$$\left\{\frac{2\left(z_{1-\frac{\alpha}{2}}+z_{\beta}\right)^{2}*\sigma^{2}}{\left|\mu_{T}-\mu_{c}\right|}\right\}=\left\{\frac{2(1.96+1.28)^{2}*1.5^{2}}{(-2)^{2}}\right\}=11.81\approx 12$$

Considering a 20% dropout rate: 11.81 / 0.8 = 14.76 → approximately 15 participants per group, total of 30 participants.

4) Reference paper: Rezaie K, Amiri A, Ebrahimi Takamjani E, Shirani G, Salehi S, Alizadeh L. The Efficacy of Neck and Temporomandibular Joint (TMJ) Manual Therapy in Comparison With a Multimodal Approach in the Patients with TMJ Dysfunction: A Blinded Randomized Controlled Trial. Med J Islam Repub Iran. 2022;36:45. doi: 10.47176/mjiri.36.45. PMID: 36128309; PMCID: PMC9448471.

9.4 무작위배정 방법, 시험대상자 식별 방법 (Randomization method, subject identification method)

- 임상시험의 수행 및 평가에 관여하지 않는 무작위배정 담당자(또는 통계 담당자)가 구체적 계획 및 재현 가능한 방법으로 무작위배정 목록을 생성하고 무작위 배정표는 봉인하여 봉인해제 여부를 확인할 수 방식으로 보관하며 시험책임자가 별도로 관리한다. (A randomization officer (or statistician) who is not involved in the conduct or assessment of the clinical trial will generate a randomization list using a detailed and reproducible method. The list will be sealed and stored in a manner that allows verification of any seal breakage, and it will be managed separately by the principal investigator.)
- 무작위배정 담당자는 Python 3.12.4 (Python Software Foundation, Wilmington, DE, USA)를 사용하여 각 개체가 뽑힐 가능성이 동일한 상태에서 무작위로 각 군에 배정한다. 블록 무작위배정 방법(block randomization)을 이용한다. 시험군과 대조군의 무작위 배정은 1:1 비율로 실시한다. (The randomization officer will use Python 3.12.4 (Python Software Foundation, Wilmington, DE, USA) to randomly assign each subject to a group under conditions where each individual has an equal chance of being selected. A block randomization method will be applied, with a 1:1 allocation ratio between the treatment group and control group.)
- 시험담당자는 대상자 앞에서 순서대로 무작위배정 봉투를 개봉하여 대상자를 배정하고 개봉한 봉투에는 개봉한 날짜와 시험담당자의 서명을 기재하여 별도 보관한다. (The trial coordinator will open the randomization envelope in front of each subject, in sequence, and assign the subject accordingly. The envelope will be labeled with the date of opening and the signature of the coordinator, and will be stored separately.)
- 임상시험 참여에 서면으로 동의하고, 스크리닝 검사를 시행하는 시험대상자에게 다음의 스크리닝 번호를 부여한다. (Subjects who provide written informed consent and undergo screening assessments will be assigned a screening number as follows:)

- DJ-S-001
  [DJ: 대전대학교 대전한방병원, S: Screening의 첫 글자, 001: 일련번호(001, 002~)]
  [DJ: Daejeon University Daejeon Korean Medicine Hospital, S: First letter of "Screening", 001: Serial number (001, 002, etc.)]

- 임상시험 참여에 동의하고, 시험대상자 적합성 평가를 통하여 선정 및 제외기준에 적합한 자에 한하여 시험대상자 식별코드를 부여한다. (Only those who provide informed consent and meet both the inclusion and exclusion criteria based on the eligibility assessment will be

assigned a subject identification code for the clinical trial:)

- DJ-E-001
  [DJ: 대전대학교 대전한방병원, E: Enrollment의 첫 글자, 001: 일련번호(001, 002~)]
  [DJ: Daejeon University Daejeon Korean Medicine Hospital, E: First letter of "Enrollment", 001: Serial number (001, 002, etc.)]

10. 임상시험 방법 (Clinical Trial Methods)

10-1 임상시험 디자인 (Clinical Trial Design)

- 본 임상시험은 무작위배정, 평가자맹검, 평행설계로 진행한다. (This clinical trial will be conducted as a randomized, assessor-blinded, parallel-group design.)
- 시험군: TBT 치료 (주 2회, 총 12회 방문하여 TBT 실시) (Experimental group: TBT (Temporomandibular Balancing Therapy), administered twice per week for 6 weeks (total 12 sessions))
- 대조군: TENS 치료 (주 2회, 총 12회 방문하여 TENS 실시) (Control group: TENS (Transcutaneous Electrical Nerve Stimulation), administered twice per week for 6 weeks (total 12 sessions)

10-2 임상시험 과정 요약 (Summary of the Clinical Trial Procedure)

- 본 시험에서는 턱관절 장애 환자를 대상으로 턱관절 장애에 대한 TBT의 안전성, 유효성 및 경제성을 확인하고자 한다. (This study aims to evaluate the safety, efficacy, and cost-effectiveness of TBT in patients with temporomandibular disorders (TMD).)
- 본 시험에 참여를 신청한 지원자 중에서 인구학적 조사, 병력 및 치료력 조사, 활력징후(혈압, 맥박, 체온), VAS 및 이학적 검진을 통하여 선정 및 제외기준에 부합하는 대상자를 선정한다. (Among applicants for participation, those who meet the inclusion and exclusion criteria will be selected through demographic survey, medical and treatment history, vital signs (blood pressure, pulse, temperature), VAS, and physical examination.)
- 대상자를 시험군(TBT 치료), 대조군(TENS 치료)로 무작위배정한 후 각 배정군에 해당하는 치료를 6주간 주 2회, 총 12회 실시한다. (Eligible participants will be randomly assigned to either the treatment group (TBT) or the control group (TENS). Each group will receive their assigned intervention twice per week for 6 weeks, for a total of 12 sessions.)
  시험 기간 6주와 추적관찰 10주 동안
  턱관절 통증 VAS(a. 지난 1주간의 평균적인 턱관절 통증, b. 지난 1주간의 가장 심했던 턱관절 통증)는 3회(스크리닝, 0주, 6주) 실시하고,
  수직 턱관절 개구도(a. Pain free opening, b. Maximum unassisted opening) 확인, JFLS-8, DC/TMD 만성통증 등급 척도 2판, 치료 치료 기대 척도 측정, PGIC, QALYs, 비용측정 및 효용측정(EQ-5D, EQ-VAS) 설문은 3회(0주, 6주, 10주) 실시한다.
  (During the 6-week treatment period and 10-week follow-up, the following assessments will be performed:
  TMJ pain VAS (a. average pain over the past week, b. worst pain over the past week): 3 times (Screening, Week 0, Week 6)

Vertical mandibular opening (a. pain-free opening, b. maximum unassisted opening), JFLS-8, DC/TMD Chronic Pain Grade Scale Version 2, Treatment Expectation Scale, PGIC, QALYs, Cost and Utility Measurement (EQ-5D, EQ-VAS): 3 times (Week 0, Week 6, Week 10))

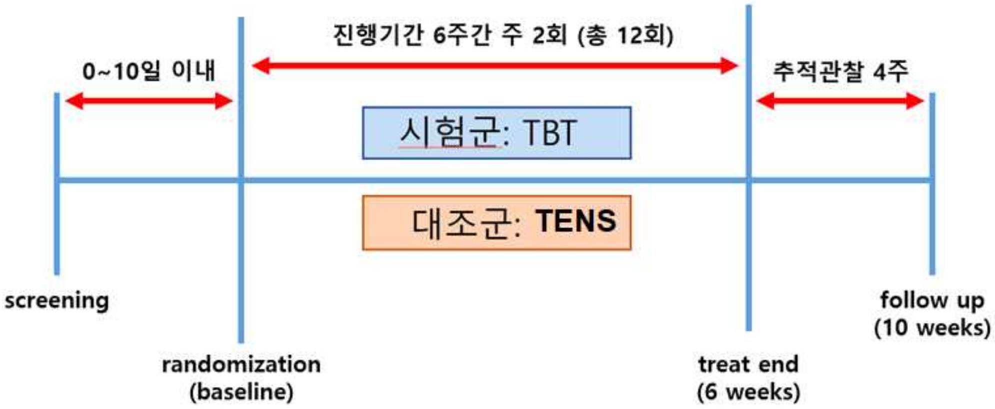

10-3 눈가림의 유지를 위한 고려 (Considerations for Maintaining Blinding)

10-3-1 시험대상자의 눈가림 유지 (Blinding of Trial Participants)

- 본 임상시험의 중재 방법은 시험대상자의 눈가림을 유지할 수 없다.
- Due to the nature of the interventions, blinding of participants is not feasible.

10-3-2 시험자 그룹의 눈가림 유지 (Blinding of Trial Personnel)

1) 임상시험 책임자 및 임상시험 담당자
   - 임상시험 담당자를 중재 담당자와 평가 담당자로 분리한다.
   - 중재 담당자는 시험대상자에 대한 중재 및 면담을 수행하고, 증례기록지를 작성한다. EMR로 인한 눈가림 해제 실수를 범하지 않기 위해 EMR 및 오더는 모두 평가자가 아닌 중재자의 이름으로 기록한다. CRF로 인한 눈가림 해제 실수를 범하지 않기 위해 CRF 서명은 중재자 또는 임상시험 책임자가 시행한다. 중재 담당자의 눈가림은 유지할 수 없다.
   - 평가 담당자는 시험대상자의 적합성 판단, 동의서 획득, 시험대상자의 일련번호 부여 및 평가를 수행한다. 평가 담당자는 시험대상자가 어떤 종류의 치료를 받는지 모르도록 한다.

1) Principal Investigator and Clinical Trial Staff
   - The clinical trial staff will be divided into intervention staff and assessment staff.
   - The intervention staff will administer the intervention and conduct interviews, and will also complete the Case Report Forms (CRFs).
   - To prevent unblinding via the Electronic Medical Record (EMR) system, all EMR entries and orders will be recorded under the intervention staff's name, not the assessor's.
   - To prevent unblinding via CRFs, CRF signatures will be completed only by the intervention staff or principal investigator.
   - Blinding of intervention staff is not maintained.
   - The assessment staff will be responsible for screening, obtaining informed consent,

assigning participant numbers, and performing evaluations.
- Assessment staff must remain blinded to the treatment group assignments.

2) 임상시험 코디네이터
- 시험대상자별 일정 관리 등 임상시험 기초업무를 수행한다.
- 증례기록지를 작성한다.
- 코디네이터의 눈가림은 유지할 수 없다.

2) Clinical Trial Coordinator
- Responsible for basic trial tasks, including schedule management for each participant.
- Also responsible for completing CRFs.
- Blinding of the coordinator is not maintained.

10.4 병용약물 투약 기준 (Criteria for Concomitant Medication)

10.4.1 병용가능 약물 (Permissible Concomitant Medications)

- 임상시험 시작 전 6주 동안 의사 처방에 따른 통증에 영향을 줄 수 있는 약물(예: 비스테로이드성 소염제)의 복용이 일정한 경우는 허용한다. 시험 기간 동안 의사 처방에 따른 약물 복용량은 일정하게 유지되어야 한다. 기타 질환의 일과성 치료를 목적으로 사용되는 약물은 연구의사와 상의를 통하여 병용 투여하기로 한다. 모든 병용약물 투여 시(타 질환 또는 이상반응 발현 시 치료약물 포함) 그 약물에 대한 정보(제품명, 투여목적, 투여용량, 투여기간 등)를 증례기록지에 상세히 기록한다. 만약 병용가능 약물의 복용량이 일정하게 유지되지 못하는 경우 시험자의 판단에 따라 임상시험에서 탈락처리될 수 있다. 6~10주 사이에는 병용 약물은 허용하며, 투여 시 그 약물에 대한 정보를 증례기록지에 기록한다. (If a participant has been consistently taking physician-prescribed medications that may affect pain (e.g., non-steroidal anti-inflammatory drugs [NSAIDs]) during the 6 weeks prior to trial initiation, they may continue these medications. During the trial period, the dosage must remain consistent. Medications used for transient treatment of other conditions may be allowed upon consultation with the study physician. For any concomitant medication (including those used for other conditions or to manage adverse events), detailed information such as product name, indication, dosage, and duration must be recorded in the Case Report Form (CRF). If dosage consistency cannot be maintained, the subject may be excluded from the trial at the investigator’s discretion. From week 6 to week 10, concomitant medications are permitted, and all details must be documented in the CRF.)

10.4.2 병용금지 약물 (Prohibited Concomitant Medications)

- 스테로이드 제제, 면역억제제, 정신질환 약물은 연구 기간 내에 병용 금지해야한다. 또한 기타 시험자 판단에 따라 시험에 영향을 미칠 수 있는 약물은 금지할 수 있다. (Steroids, immunosuppressants, and psychiatric medications are prohibited during the study period. Other medications may also be prohibited at the discretion of the investigator if they may affect the trial outcomes.)

11. 관찰항목별 검사 및 시험 일정 (Assessments and Study

Schedule)

11.1 관찰항목별 방법 (Assessment Methods by Category)

11.1.1 시험대상자 동의, 스크리닝 번호 부여 및 인구학적 조사 (Informed Consent, Screening Number Assignment, and Demographic Data Collection)

- 본 임상시험을 실시하기에 앞서, 시험자는 ‘시험대상자 동의 설명서’에 관한 내용을 대상자 본인에게 설명하고, 대상자가 내용을 잘 이해한 것을 확인한 다음, 본인의 자유의사에 따른 임상시험 참가 동의를 문서로 받는다. 동의를 받는 순서에 따라 스크리닝 번호를 부여한 후 인구학적 정보를 조사한다. (Prior to participating in the clinical trial, the investigator will explain the contents of the "Informed Consent Form" to the subject. Written informed consent must be obtained after confirming that the subject fully understands and voluntarily agrees to participate. A screening number is assigned in the order of consent, and demographic data are collected.)
- 시험대상자 스크리닝 식별코드는 다음 방법에 따라 기록한다. (Screening identification codes are recorded as follows:)
  - DJ-S-ZZZ
  [DJ: 대전대학교 대전한방병원, S: Screening의 첫 글자, ZZZ: 일련번호(001, 002~)]
  [DJ: Daejeon University Daejeon Korean Medicine Hospital, S: First letter of "Screening", ZZZ: serial number (001, 002, ...)]
  (예: DJ-S-012: 대전대학교 대전한방병원에서 12번째 스크리닝 지원자)
  (e.g., DJ-S-012: 12th screening participant at Daejeon University Daejeon Korean Medicine Hospital)
- 기록사항은 서면 동의 여부 및 동의 일자, 시험대상자 이니셜, 성별, 생년월일, 연령, 주소 및 연락처 등이다. (Collected information includes informed consent status and date, subject initials, gender, date of birth, age, address, and contact information.)

11.1.2 이학적 조사, 병력 및 치료력 조사 (Physical Examination, Medical History, and Treatment History)

- 임상시험에 들어가기 전에 시험대상자의 이학적 조사, 병력 및 치료력 등에 대해 면담을 통하여 확인하고 증례기록지에 기록한다. (Before trial initiation, the investigator conducts interviews to assess the subject’s physical status, medical history, and prior treatments, and records the findings in the CRF.)

11.1.3 시험대상자 적합성 평가 (Eligibility Assessment)

- 선정 및 제외기준에 따라 시험대상자로 적합한지 인구학적 조사, 병력 및 치료력 조사, 활력징후, VAS, 이학적 검진 등을 통해 평가한다. (Eligibility is assessed based on inclusion and exclusion criteria, using demographic data, medical and treatment history, vital signs, VAS, and physical examination.)

11.1.4 시험대상자 식별코드 부여 (Assignment of Subject Identification Code)

- 선정 및 제외기준에 따라 시험 참여가 적합한 자에 한해 시험대상자 식별코드를 부여한다. (Only participants who meet the inclusion and exclusion criteria will be assigned a subject identification code.)
- 시험대상자 식별코드는 다음 방법에 따라 기록한다. (Subject codes are recorded as follows:)
  - DJ-S-ZZZ
  [DJ: 대전대학교 대전한방병원, E: Enrollment의 첫 글자, ZZZ: 일련번호(001, 002~)]
  [DJ: Daejeon University Daejeon Korean Medicine Hospital, E: First letter of "Enrollment", ZZZ: serial number (001, 002, ...)]
  (예) DJ-E-015: 대전대학교 대전한방병원에서 15번째 등록 시험대상자)
  (e.g., DJ-E-015: 15th enrolled subject at Daejeon University Daejeon Korean Medicine Hospital)

11.1.5 임상검사항목 (Clinical Assessments)

1) 활력징후 (Vital signs)
   - 매 방문마다 급격한 체위 변동 없이 앉은 자세를 최소 3분 이상 유지한 상태에서 혈압, 맥박수를 측정한다. 체온은 실내온도에서 충분히 안정을 취한 상태에서 측정한다. (At each visit, blood pressure and pulse are measured after the subject remains seated for at least 3 minutes without postural changes. Body temperature is measured after the subject is sufficiently stabilized at room temperature.)

2) 임신진단검사 (Pregmancy test)
   - 폐경이 되지 않은 여성 대상자는 Urine HCG를 시행하며 결과는 음성이어야 한다. 단, Screening에서 menstruation에는 visit1에 시행한다. (Female participants who are not postmenopausal must undergo a urine hCG test, and results must be negative. If menstruation occurs during screening, the test will be performed at Visit 1.)

3) 턱관절 통증 VAS (100mm) (TMJ Pain VAS, 100mm)
   - VAS(Visual analog scale)는 최대 100mm의 시각평가가로자를 이용하여 환자가 직접 불편한 정도를 표기하게 하는 검사이다. 환자가 느끼는 주관적인 통증의 정도를 수치화하여 명확하게 시각화한다. a. 지난 1주간의 평균적인 턱관절 통증, b. 지난 1주간의 가장 심했던 턱관절 통증으로 나누어 평가한다. (The Visual Analog Scale (VAS) is a 100 mm horizontal line on which the subject marks their level of discomfort. It provides a quantified and visual representation of the subject’s subjective pain. Pain is evaluated based on: a. Average TMJ pain over the past week b. Worst TMJ pain experienced over the past week)
   - 시각평가가로자 상 0에 가까울 수록 '전혀없다', 100에 가까울 수록 '극심하다'를 나타낸다. 지난 1주간의 평균적인 턱관절 통증정도와 지난 1주간의 가장 심했던 통증 정도를 응답하도록 되어있다. (On the scale, 0 mm indicates "no pain at all" and 100 mm indicates "worst imaginable pain". Subjects are instructed to report both the average and the most intense TMJ pain they experienced during the past week.)

4) 이학적 검진 (Physical examination)

• 검사자는 ‘측두하악장애 진단기준(DC/TMD): 평가도구(한국어)’의 Axis I 중 'Examination: Pain-related Interview and Examiner Commands' 및 ‘DC/TMD Examination Form’ 내용에 따라, 환자와의 인터뷰 및 환자의 턱관절 부위의 촉진을 통해 저작근 또는 턱관절의 통증 여부를 확인한다. 환자의 턱관절 통증이 근육통, 근막통증, 관절통에 해당하는지 확인해야하며, 이를 기록한다.

• ‘DC/TMD Examination Form’은 TMD의 진단 및 세부 진단을 위한 평가도구로, 12가지 카테고리로 구성된다.

Location of pain & headache (last 30 days) 지난 30일 동안 통증과 두통의 위치.
Pain duration and intensity 통증의 지속 시간과 강도에 대한 정보.
Incisal relationships 상하악 절치의 교합 상태(오버젯, 오버바이트 등).
Opening pattern 하악 개구 시 움직임의 패턴(직선, 곡선 등).
Opening movements 하악 개구 시 운동 범위 및 제한 여부.
Maximum unassisted and assisted opening 환자 스스로 가능한 최대 개구량 및 외부 보조를 통해 가능한 최대 개구량.
Lateral and protrusive movements 하악의 측방 및 전진 운동 범위.
Joint sounds 턱관절에서 들리는 잡음.
Palpation tenderness (TMJ and muscles) 턱관절과 저작근 촉진 시 압통 여부.
Deviation during opening 개구 시 하악의 편위 여부.
Pain provocation tests 특정 움직임이나 촉진 시 통증 유발 검사 결과.
Headache and referred pain 두통 및 연관 통증의 유무 및 위치.

• DC/TMD 진단 기준에 따르면 턱관절 장애는 (1) 근육 장애, (2) 관절 디스크 장애, (3) 관절 질환의 3가지 주요 진단군으로 분류될 수 있는데, 본 연구에서는 3가지 주요 진단군 중 (1) 근육 장애를 반드시 가지고 있는 턱관절 장애 환자를 대상으로 본 연구를 수행하고자 하였으며, 연구 대상자는 (2) 관절 디스크 장애, (3) 관절 질환을 동반할 수도, 동반하지 않을 수도 있다. 따라서 본 연구에서 이학적 검진은 ‘DC/TMD Examination Form’ 중 디스크 장애에 대한 내용을 제외하고 근육통, 근막통증, 관절통의 진단에 대한 내용을 발췌하여(‘DC/TMD 평가표’로 명명함) 수행한다.

• According to the DC/TMD diagnostic criteria, temporomandibular disorders (TMD) are classified into three major diagnostic groups: (1) Muscle Disorders, (2) Disc Displacement Disorders, and (3) Joint Disorders. This study will be conducted exclusively on TMD patients who have Muscle Disorders (Group 1). Participants may or may not also present with Disc Displacement or Joint Disorders (Groups 2 and 3). Therefore, the physical examination in this study will extract only items related to myalgia, myofascial pain, and arthralgia from the DC/TMD Examination Form, excluding items pertaining to disc displacement. This adapted form is referred to as the “DC/TMD Evaluation Form” in the study.

5) 수직 턱관절 개구도 확인 (Vertical Mandibular Opening Measurement)

• 통증이 없는 범위 내에서의 환자의 최대 개구도와 통증과 상관없이 환자 스스로 가능한 최대 개구도를 확인한다. 수직 턱관절 개구도의 확인은 술자를 이용하여 측정하며, 필요 시 설압자도 함께 사용하여 수치를 측정한 뒤 기록하도록 한다. (a. Pain free opening, b. Maximum

unassisted opening)

• Two types of vertical opening are assessed: a. Pain-free opening (maximum opening without pain), b. Maximum unassisted opening (maximum opening regardless of pain). Measurements are taken using a standardized measuring tool and, if necessary, with a tongue depressor to aid in accurate measurement.

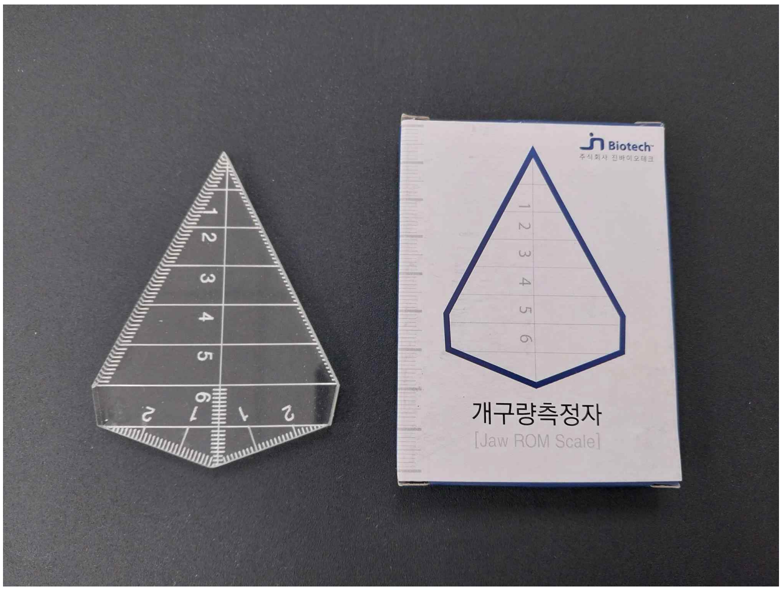

* 개구량 측정자 (제조사: (주)진바이오테크, 제조국: 한국)

* Measuring Device: Manufactured by Jin Biotech Co., Ltd., Republic of Korea

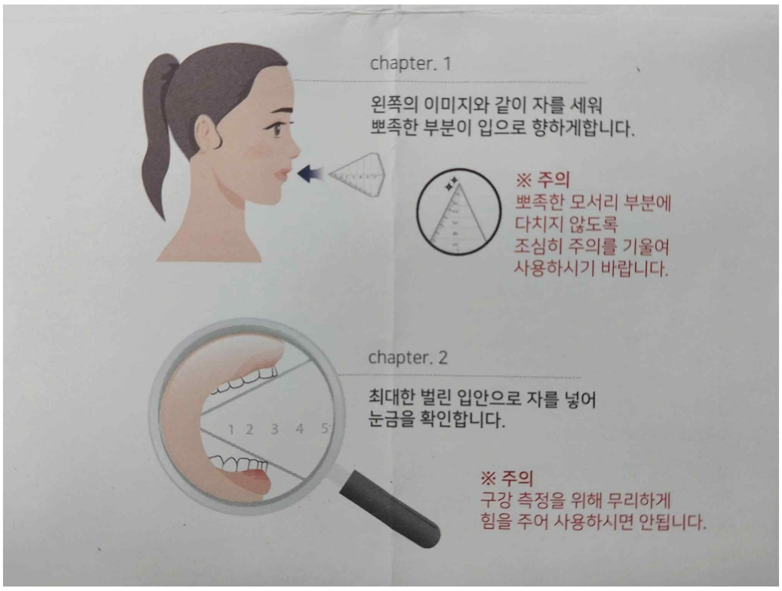

* 개구량 측정 방법 (Measuring method)

6) 턱기능 제한지수 (Jaw Functional Limitation Scale ,JFLS-8)

• 턱기능 제한지수(JFLS-8)는 턱관절 장애 환자의 전반적인 턱기능에 대해 평가하기 위해 8개의

항목으로 구성된 설문지이다. 턱 운동, 저작기능에 대한 문항이 있다. (The JFLS-8 is an 8-item questionnaire designed to assess overall jaw function in TMD patients, including jaw movement and masticatory ability.)

• 각 문항마다 0점~10점으로 채점되며 총점은 0점~10점으로 기록된다. 점수가 낮을 수록 장애 정도가 경미한 상태를 의미하며, 점수가 높을 수록 장애 정도가 심한 상태를 의미한다. (Each item is scored from 0 to 10, and the total score ranges from 0 to 10 (averaged). Lower scores indicate milder dysfunction, and higher scores indicate more severe dysfunction.)

7) DC/TMD 만성통증 등급 척도 2판 (DC/TMD Chronic Pain Grade Scale Version 2)

• DC/TMD 만성통증 등급 척도 2판은 '측두하악장애 진단기준(DC/TMD): 평가도구(한국어)'의 Axis II 평가도구 중 하나로 턱관절 장애 환자의 통증 및 사회적 상태에 대해 평가한다. (This scale is part of Axis II in the "Diagnostic Criteria for Temporomandibular Disorders (DC/TMD): Assessment Tools (Korean version)" and evaluates both pain intensity and its impact on social functioning.)

• 환자들은 통증의 강도 및 사회적 활동에 지장을 받는 정도에 대한 질문에 0점~10점 사이로 수치화하여 기록한다. 0점에 가까울수록 "통증 없음" 또는 "방해받지 않음"이며, 10점에 가까울수록 "가능한 최고의 통증" 및 "어떤 활동도 할 수 없음"이다. (Patients rate their pain and its interference with daily activities on a scale of 0 to 10, where 0 indicates "no pain or interference," and 10 indicates "worst pain imaginable or complete functional disability.")

• 원본에서는 "지난 6개월간" 또는 "지난 30일 동안"에 대한 내용을 평가하게 되어있으나, 본 연구에서는 본 척도를 유효성 평가변수로 사용하기 위해 "지난 6개월간"과 "지난 30일 동안"을 "지난 4주간"으로 기간을 통일한다. (While the original scale assesses pain over the past 6 months or past 30 days, this study standardizes the reference period to the past 4 weeks for consistency in efficacy evaluation.)

8) 치료 기대 척도 (Treatment Expectancy Scale (Credibility and Expectancy))

• 치료 기대 척도는 턱관절 장애 환자들의 치료에 대한 기대감을 평가하기 위한 척도로, 5점 척도인 Likert scale을 이용한다. (This scale measures the patient's expectation regarding treatment effectiveness, using a 5-point Likert scale.)

• 환자들은 "TBT(대조군의 경우 TENS) 치료가 턱관절 장애와 관련된 통증과 증상을 얼마나 경감시킬 것으로 생각되십니까?" 라는 질문에 대한 환자의 대답을 1점~5점 사이로 수치화하여 기록한다. 1점에 가까울 수록 "전혀 경감시키지 못할 것으로 생각한다."이며, 5점에 가까울 수록 "몹시 경감시킬 것으로 생각한다."이다. (Patients respond to the question: "How much do you think the TBT treatment (or TENS, for the control group) will reduce your TMJ-related pain and symptoms?" A score of 1 means "Not at all effective," and 5 means "Extremely effective.")

9) PGIC (Patient Global Impression of Change)

• PGIC는 턱관절 장애 환자가 자신의 증상에 대한 호전 정도를 주관적으로 평가하도록 하는 방법이다. (PGIC is a self-rated scale that allows participants to assess the overall change in their condition.)

• 이는 7단계로 분류하며, 1점에 가까울 수록 "매우 호전되었다"이며, 7점에 가까울 수록 "몹시 악화되었다."이다. 중간 점수인 4점은 "변화가 없다."를 나타낸다. (The scale consists of 7 levels: 1 = Very much improved, 4 = No change, 7 = Very much worse)

10) Quality-adjusted life years (QALYs)

• 질보정수명(QALY)은 경제성 평가 결과지표의 일종으로, 의료기술로 인한 건강결과 개선을 측정할 때 생존연수(life years) 뿐만 아니라 해당 생존기간 동안의 삶의 질을 보정하기 위해 만들어진 지표로, 삶의 양과 삶의 질을 모두 고려하는 지표이다. 일반적으로 1년 동안의 1 QALY는 1년 동안 완벽한 건강상태를 의미한다. 질보정수명은 건강상태에 머문 기간별로 해당 건강상태의 질 가중치를 곱하여 총합으로 산출하며(area under the curve 방법), 효용(utility) 또는 건강관련 삶의 질(health-related quality of life, HRQOL)은 건강상태에 대한 선호(preference)를 기반으로 측정된다. (QALY is a health economic outcome that reflects both the quantity and quality of life gained through medical intervention. 1 QALY equates to one year in perfect health. QALYs are calculated using the area under the curve (AUC) method by multiplying the time spent in each health state by the corresponding utility weight, a value reflecting the preference for that state.)

• 건강관련 삶의 질은 가치평가(valuation) 또는 HRQOL 측정도구를 활용하여 측정할 수 있으며, 일반적으로 선호도 기반 도구인 EQ-5D에 인구집단 별로 개발된 질 가중치 산출식을 적용하여 국가별 질 가중치를 도출할 수 있다. 본 연구에서는 Kim 등(2016)의 연구에서 개발한 한국인 대상 질 가중치 점수체계(tariff)를 적용하여 시험대상자별 질보정수명을 산출한다. (Utility is typically measured using preference-based instruments like the EQ-5D. In this study, QALYs are derived by applying the Korean-specific value set (tariff) developed by Kim et al. (2016).)

11) The 5-level EuroQol-5 dimension (EQ-5D-5L)

• EQ-5D는 1987년에 설치된 EuroQol 그룹에 의해 설치되고 연속적으로 개발되었다. Korean EuroQol-5 dimension (KEQ-5D)는 건강관련 삶의 질을 효용(utility)으로 측정하는 도구로 다차원적 선호도 근거 건강 관련 삶의 질 측정도구(multidimensional preference based HRQOL measure)이다. 본 시험에서는 교차-문화적 적응 작업과 확인 과정이 완료된 한국어판 EQ-5D-5L을 사용한다. EQ-5D는 두 가지 방법으로 삶의 질을 평가한다. 먼저 기술체계(descriptive system)를 이용하여 운동능력, 자기관리, 일상활동, 통증/불편, 불안/우울 등 5개 영역에서 삶의 질을 평가한다. 각 영역은 5단계로 평가하는데, 문제가 없을 때 1점에 표시를 하고 문제가 심각할 때 5점에 표시를 한다. 따라서 점수가 낮을수록 HRQoL은 높다. 둘째로 20cm의 세로 선으로 구성된 유추 척도(visual analogue scale: VAS)를 이용하여, 전반적인 건강상태에 대한 평가를 시행한다. 점수는 최저의 건강상태를 0점으로 최고의 건강상태를 100점으로 표시하는데, 점수가 높을수록 HRQoL이 높다. (The EQ-5D is a standardized preference-based HRQoL instrument developed by the EuroQol Group to measure health-related quality of life. The Korean version of the EQ-5D-5L, adapted through cross-cultural validation, will be used in this study. It consists of: a. A descriptive system that assesses five dimensions: mobility, self-care, usual activities, pain/discomfort, and anxiety/depression. Each dimension is scored on a 5-point scale, where 1 = no problems

and 5 = extreme problems. Lower scores indicate better health. b. A Visual Analogue Scale (EQ-VAS): a vertical scale from 0 (worst imaginable health) to 100 (best imaginable health), on which patients rate their overall health.)

12) EuroQol Visual analog scale (EQ-VAS)
  • 전반적인 건강상태에 대한 평가를 시행한다. 점수는 최저의 건강상태를 0점으로 최고의 건강상태를 100점으로 표시하며 점수가 높을수록 HRQoL이 높다. (The EQ-VAS is a self-rated visual scale that measures overall health status. A score of 0 indicates the worst imaginable health, and 100 indicates the best imaginable health. Higher scores reflect better HRQoL.)

13) 비용 측정 (Cost measurement)
  • 경제성 평가를 위한 비용 평가 항목은 의료비용, 비의료비용, 생산성손실비용으로 구성되며 분석 관점에 따라 포함되는 비용 항목이 달라진다. 본 연구는 기본 분석으로 보건의료체계 관점을 채택하여 의료비용(공식적 의료비용, 비공식적 의료비용)을 포함하며, 민감도 분석으로 사회적 관점을 채택하여 의료비용(공식적 의료비용, 비공식적 의료비용), 비의료비용(교통비용), 생산성손실비용(이환비용)을 포함한다. 비용 산출을 위한 자료원으로 기관 비용자료, 시험대상자 설문 결과, 통계청 자료, 선행 연구 문헌 등을 활용하여 시험대상자별 총비용을 산출한다. (For economic evaluation, cost categories include: Direct medical costs (e.g., consultations, procedures), Non-medical costs (e.g., transportation), Productivity loss costs (e.g., absenteeism, presenteeism). The base-case analysis adopts a healthcare system perspective, including direct medical costs (both formal and informal). The sensitivity analysis adopts a societal perspective, incorporating all categories: direct medical costs, non-medical costs (e.g., transportation), and productivity loss. Data sources include: Institutional cost records, Participant questionnaires, Statistics Korea data, Published literature, Total costs per participant will be calculated.)
  • 비용 평가 항목 중 생산성손실비용은 Work Productivity and Activity Impairment Questionnaire: General Health V2.0 (WPAI:GH 2.0) 한국어 버전을 이용하여 수집한다. (Productivity loss is assessed using the Korean version of the Work Productivity and Activity Impairment Questionnaire: General Health V2.0 (WPAI:GH 2.0).)

[비용 평가 항목]
[Cost Evaluation Items]

대분류 Categroy	세분류 Subcategory	항목 Item	자료원 Source	수집 시기 Collection period
의료비용 Medical expenses	공식적 의료비용 Official medical expenses	TBT 적용 비용 TBT application cost	기관 자료 Institutional data	-
		TENS 적용 비용 TENS application cost	기관 자료 Institutional data	-
		타의료기관 진료 비용 (입원/외래/응급실/처방약물)	시험대상자 설문(CRF) Subject Response Form (CRF)	방문 8, 9 (6주, 10주) Visits 8, 9 (weeks 6 and 10)
	비공식적 의료비용 Unofficial medical expenses	비처방약물 및 건강기능식품 Non-prescription drugs and health functional foods	시험대상자 설문(CRF) Subject Response Form (CRF)	방문 8, 9 (6주, 10주) Visits 8, 9 (weeks 6 and 10)
		기타 의료기기, 기구, 치료재료, 민간요법 등 Other medical devices, instruments, therapeutic materials, folk remedies, etc.	시험대상자 설문(CRF) Subject Response Form (CRF)	방문 8, 9 (6주, 10주) Visits 8, 9 (weeks 6 and 10)
비의료비용 Non-medical expenses		교통비용 Transportation costs	국민건강영양조사, 통계청 자료 National Health and Nutrition Survey, Statistics Korea data	-
생산성손실비용 Loss of productivity costs		이환비용 Morbidity costs	시험대상자 설문(CRF), 통계청 자료 Subject Response Form (CRF), Statistics Korea data	방문 1, 8, 9 (0주, 6주, 10주) Visits 1, 8, 9 (weeks 0, 6, 10)

14) X-Ray 검사 (X-Ray Examination)

스크리닝에서 대상자 안전성 확보(개구도 평가, 턱관절에 수기요법 적용, TENS를 저작근에 직접 적용, 수술 이력 대상자 제외 등)를 위한 X-Ray 검사를 시행한다. T.M Joint View 및 Open Mouth View를 촬영한다.

In screening, X-Ray examination is performed to ensure the safety of the subjects (evaluate mouth opening, apply manual therapy to temporomandibular joint, apply TENS directly to masticatory muscles, exclude subjects with a history of surgery, etc.). T.M Joint View and Open Mouth View are taken.

11.1.7 순응도 평가 (Compliance Assessment)

- Visit 13 이후 중재에 대한 순응도를 아래와 같이 평가하도록 한다.
- 시험 참여 기간 동안의 전체 순응도는 2/3(66.6%) 이상이어야 하며, 2/3(66.6%) 미만인 경우 해

당 시험대상자는 순응도가 나쁜 것으로 간주하여 PP분석군에서 제외한다.

$$\text{순응도} = \frac{\text{실제 실수된 중재횟수}}{\text{계획된 중재횟수}} \times 100$$

- 단, 위 순응도 계산식은 연구가 종결된 시점에서 활용하는 계산식이며, Visit 2부터 Visit 12까지 매 방문 시 대상자의 순응도를 평가할 때에는 '예상되는 최대 중재횟수를 12로 나눈 값'을 활용하여 순응도를 계산한다.
- After Visit 13, the compliance to the intervention will be evaluated as follows.
- The overall compliance during the trial participation period must be 2/3 (66.6%) or higher. If it is less than 2/3 (66.6%), the subject will be considered to have poor compliance and will be excluded from the PP analysis group.
- However, the above compliance calculation formula is used at the end of the study, and when evaluating the subject's compliance at each visit from Visit 2 to Visit 12, the 'value calculated by dividing the expected maximum number of interventions by 12' will be used to calculate the compliance.

$$\text{Compliance} = \frac{\text{Number of interventions actually performed}}{\text{Number of interventions planned}} \times 100$$

11.1.8 이상반응 조사 (Adverse Event Monitoring)

- 시험책임자 혹은 연구의사는 임상시험 참여 후 나타나는 이상반응 여부를 방문 시 마다 시험대상자에 대한 진찰로서 관찰하여 시험 치료법과의 인과관계에 대하여 '이상반응 기록표'에 기록한다.
- 이상반응 발현 시 발현일 및 소실일, 이상반응의 정도 및 결과, 시험 치료법과 관련하여 취해진 조치 및 시험 치료법과의 인과관계, 시험 치료법 이외 의심되는 원인, 이상반응에 대한 치료 여부 및 내용 등을 상세히 기록한다.
- The principal investigator or study physician will examine each participant at every visit to observe the occurrence of any adverse events (AEs) following participation in the clinical trial.
- The findings, including the causal relationship between the adverse event and the intervention, will be recorded in the Adverse Event Report Form. In the event of an adverse reaction, the following details will be thoroughly documented: Date of onset and resolution, Severity and outcome of the event, Actions taken in response to the event, Causal relationship to the intervention, Suspected causes other than the intervention, and Whether treatment was administered for the adverse event and the details of such treatment

11.1.9 활력징후에 대한 점검 (Vital Signs Monitoring)

- 각 방문 시 체온, 맥박, 호흡수 및 혈압을 기록하고 이상이 있다면 약물투여 및 시험약과의 인과관계를 평가한다.
- At each visit, body temperature, pulse, respiratory rate, and blood pressure will be

measured. If any abnormalities are detected, the necessity of medication and the causal relationship with the intervention will be evaluated.

11.1.10 면담을 통한 점검 (Interview-Based Monitoring)

- 중재자는 매 중재 수행 이후 대상자의 불편감을 확인하고 및 중재 방법의 안전성을 평가하기 위해 면담을 진행한다.
- 평가자는 시험 시작 후 6주(12회차), 10주(13회차) 방문 시에 시행하는 평가와 함께 간단한 면담을 통해 검사 내용에 지장을 줄 수 있는 요인(음주, 심각한 피로 등)이 있었는지 확인하며, 해당 사항이 있을 시 이를 기록한다.
- After each intervention session, the interventionist will conduct an interview to assess the participant’s discomfort and to evaluate the safety of the intervention.
- The evaluator will also conduct brief interviews during the assessments at Week 6 (Visit 12) and Week 10 (Visit 13) to check for any factors that may have influenced the results of the evaluation (e.g., alcohol consumption, severe fatigue, etc.). If such factors are present, they will be recorded accordingly.

11.2 시험 일정

11.2.1 임상시험 일정 (Clinical Trial Schedule)

기간period	스크리닝screening & 처치treatment				추적관찰 follow-up
주week	-1	0~5W±1		6W±1	10W±5
방문visit[1]	스크리닝	1	2~11	12	13
동의서 취득 Obtain informed consent form	●				
인구학적 조사 Demographic survey	●				
병력 및 치료력 조사 Medical and treatment history	●	●	●	●	●
활력징후 측정 Vital signs	●	●	●	●	●
선정/제외기준 확인 Check inclusion/exclusion criteria	●				
X-Ray검사[2] X-Ray examination	●				
이학적 검진[3] Physical examination	●				
임신진단검사[4] Pregnancy test	●				
무작위 배정 Random assignment		●			
병용약물 확인 Check concomitant medications		●	●	●	●
이상반응 확인 Check for adverse events		●	●	●	●
TBT[5] 또는 TENS[6] TBT or TENS		◇	◇	◇	
턱관절 장애 교육 Patient education		●			
턱관절 통증 VAS TMJ pain VAS	●	●		●	●
수직 턱관절 개구도 확인 Check the vertical TMJ opening		●		●	●
JFLS-8 Jaw Functional Limitation Scale-8		●		●	●
만성통증 척도 2.0[7] DC/TMD The Graded Chronic Pain Scale (GCPS 2.0)		●		●	●
치료 기대 척도 Treatment Expectancy Scale		●		●	●
PGIC Patient Global Impression of Change		●		●	●
효용측정[8] Utility measurement		●		●	●

**비용측정**[9)] Cost measurement		●		●	●
**순응도 확인**[10)] Check compliance			●	●	●
**방문일정 교육** Visit schedule information		●	●	●	

1) 방문1은 Screening으로부터 0-10일 이내에 행해져야 한다. Screening에서 선정제외 기준을 모두 충족하는 것을 확인한다면, 방문1이 동일한 날에 시행될 수 있다. 방문1은 Baseline 방문이며, 중재 시술 후 주 2회 방문한다. 방문허용일은 Baseline으로부터 각 주차별 해당하는 날짜±1일로 하며, 해당 날짜가 주말인 경우 가장 가까운 평일로 대체한다. 추적관찰은 Baseline으로부터 10주±5일의 허용일을 둔다. Screening과 방문1이 동일한 날인 경우 중복되는 검사나 설문은 1회만 시행한다. (Visit 1 must be conducted within 0–10 days from the screening. If the subject meets all inclusion and exclusion criteria during the screening, Visit 1 may be conducted on the same day. Visit 1 serves as the Baseline visit, after which the participant will visit twice per week for the intervention. Visit windows are defined as ±1 day from the scheduled date based on the Baseline date; if the scheduled date falls on a weekend, it may be rescheduled to the nearest weekday. Follow-up is scheduled at 10 weeks ±5 days from the Baseline. If Screening and Visit 1 occur on the same day, any overlapping tests or questionnaires will be conducted only once.)
2) T.M Joint View 및 Open Mouth View (T.M. Joint View and Open Mouth View will be conducted.)
3) 이학적 검진은 ‘측두하악장애 진단기준(DC/TMD): 평가도구(한국어)’의 Axis I 의 'Examination: Pain-related Interview and Examiner Commands' 및 ‘DC/TMD Examination Form’ 중 디스크 장애에 대한 내용을 제외하고 근육통, 근막통증, 관절통의 진단에 대한 내용을 발췌하여 수행한다. (Physical examination will follow the "Diagnostic Criteria for Temporomandibular Disorders (DC/TMD): Assessment Instruments (Korean Version)", using Axis I’s "Examination: Pain-related Interview and Examiner Commands" and the "DC/TMD Examination Form" excluding items related to disc disorders. Only diagnostic procedures related to myalgia, myofascial pain, and arthralgia will be performed.)
4) 임신진단검사: 폐경이 되지 않은 여성 대상자는 Urine HCG를 시행하며 결과는 음성이어야 한다. 단, Screening에서 menstruation에는 visit1에 시행한다. (Pregnancy Test: Female participants who are not postmenopausal must undergo a urine hCG test, and the result must be negative. If menstruation is ongoing during screening, the test will be performed at Visit 1.)
5) ◇로 표기한 항목은 배정군에 따라 차등적으로 실시하며, TBT로 표시한 항목은 모두 시험군에 한해 실시한다. (Items marked with ◇ will be conducted differently depending on the assigned group. Items marked TBT will be performed only for the treatment group.)
6) ◇로 표기한 항목은 배정군에 따라 차등적으로 실시하며, TENS로 표시한 항목은 모두 대조군에 한해 실시한다. (Items marked with ◇ will be conducted differently depending on the assigned group. Items marked TENS will be performed only for the control group.)
7) 만성통증 척도 2.0은 ‘측두하악장애 진단기준(DC/TMD): 평가도구(한국어)’의 Axis II 의 ‘만성통증 등급척도 2판’을 사용하여 수행한다. (The Chronic Pain Grade Scale 2.0 will be conducted using the "DC/TMD: Assessment Instruments (Korean Version)", specifically Axis II’s "Graded Chronic Pain Scale Version 2.0".)
8) 효용측정은 EQ-5D, EQ-VAS를 사용하여 수행한다. (Utility measurement will be conducted using EQ-5D and EQ-VAS.)
9) 비용측정은 의료비용, 생산성손실비용의 조사를 위하여 별도 개발된 비용 조사지를 사용하여 조사한다. (Cost measurement will be conducted using a separately developed cost survey form to assess medical costs and productivity loss costs.)
10) Visit 2부터 Visit 12까지는 매 방문 시 대상자의 순응도를 평가할 때에는 예상되는 최대 중재횟수를 12로 나눈 값을 활용하여 계산한다. (From Visit 2 to Visit 12, participant compliance will be evaluated at each visit. Compliance will be calculated by dividing the number of completed sessions by the expected total number of interventions (12).)

11.2.2 선정 방문(-1주) Screening Visit (Week -1)

- 내원 혹은 전화로 임상시험 참여를 신청한 지원자를 대상으로 다음 순서에 따라 스크리닝을 한다.
- 지원자가 서명한 시험대상자 동의서를 얻는다. 이 시험에 참여하는 지원자는 시험대상자 동의를 얻기 위한 설명을 듣고 자유의사에 의해 동의서에 서면 동의를 한 후 시험에 참여할 수 있다.
- 스크리닝 번호 부여 후, 인구학적 정보 및 병력, 치료력 등에 대한 문진을 실시한다.
  - 인구학적 정보: 시험대상자 이니셜, 성별, 생년월일, 연령, 흡연력, 음주력 등
  - 병력 및 치료력 조사: 주소증, 발병일, 발병인자, 현병력, 과거력, 약물투여력 등
  - 활력징후 측정: 혈압, 맥박, 체온, 호흡수
- 턱관절 통증에 대한 VAS 점수를 확인한다. (a. 지난 1주간의 평균적인 턱관절 통증, b. 지난 1주간의 가장 심했던 턱관절 통증)
- X-Ray 검사를 시행한다.
  - T.M Joint View
  - Open Mouth View
- 이학적 검진을 시행한다.
  - 검사자는 '측두하악장애 진단기준(DC/TMD): 평가도구(한국어)'의 Axis I 중 'Examination: Pain-related Interview and Examiner Commands' 및 'DC/TMD Examination Form'중 디스크 장애에 대한 내용을 제외하고 근육통, 근막통증, 관절통의 진단에 대한 내용을 발췌하여('DC/TMD 평가표'로 명명함) 이학적 검진을 시행한다. 이는 시험 대상자와의 인터뷰 및 대상자의 턱관절 부위의 촉진을 통해 저작근 또는 턱관절의 통증 여부를 확인하는 것으로, 이를 통해 환자의 턱관절 통증이 근육통, 근막통증, 관절통에 해당하는지 확인하여, 이를 기록한다.
- 종합적으로 선정, 제외기준을 확인한다.
- 다음 방문일을 지정한다.
- Screening will be conducted in the following sequence for applicants who visit the clinic or contact by phone to participate in the clinical trial.
- The participant must sign the Informed Consent Form (ICF). Only those who receive a full explanation about the trial and voluntarily provide written consent are eligible to participate.
- After assigning a screening number, the following interviews and assessments are conducted:
- Demographic information: participant initials, sex, date of birth, age, smoking history, alcohol consumption history
- Medical and treatment history: chief complaint, date of onset, contributing factors, present illness, past medical history, medication history
- Vital signs: blood pressure, pulse, body temperature, respiratory rate
- The VAS (Visual Analog Scale) score for TMJ pain is evaluated:
  a. Average TMJ pain over the past week
  b. Worst TMJ pain experienced over the past week
- X-ray imaging is performed:
  TM Joint View
  Open Mouth View
- A physical examination is conducted:
- The examiner uses the "Diagnostic Criteria for Temporomandibular Disorders (DC/TMD): Assessment Instruments (Korean version)", specifically Axis I's "Examination: Pain-related Interview and Examiner Commands".
- Only the diagnostic items related to myalgia, myofascial pain, and arthralgia are extracted and used, excluding items related to disc disorders.
- This adapted version is referred to as the "DC/TMD Evaluation Form" in this study.
- The physical examination includes an interview and palpation of the masticatory muscles and TMJ area, to determine whether the participant's TMJ pain is due to muscle pain, myofascial pain, or joint pain, and this is documented.
- The participant's eligibility is assessed according to the inclusion and exclusion criteria.
- The date for the next visit is scheduled.

11.2.3 시험 시행 (Trial Implementation)

11.2.3.1 방문 1(Baseline, 0주) (Visit 1 (Baseline, Week 0))

- 선정방문 후 무작위배정 번호를 부여하고 다음의 평가를 실시한다.
- 무작위배정을 실시하여 식별 코드를 부여한다.
- 시험대상자의 병력이나 치료력의 변화여부를 확인한다.
- 병용약물 변화여부를 확인한다.
- 활력징후를 측정한다.
- 중재 시술 전 턱관절 통증 VAS(a. 지난 1주간의 평균적인 턱관절 통증, b. 지난 1주간의 가장 심했던 턱관절 통증), 수직 턱관절 개구도(a. Pain free opening, b. Maximum unassisted opening), JFLS-8, 만성통증 척도 2.0, 치료 기대 척도 평가를 실시한다.
- 시험군은 TBT 치료를 실시하며, 대조군은 TENS 치료를 실시한다.
- 턱관절 장애 교육에 대해 실시한다.
  - 턱관절 장애 교육은 군별 배정에 관계없이 모든 참가자를 대상으로 실시한다.
  - 턱관절 장애의 원인, 예방, 치료와 관리 및 자가교정, 스트레칭법에 대한 교육 자료를 통해 시행될 것이다.
- 중재 시술 이후 이상반응을 확인한다.
- 중재 시술 이후 PGIC, 비용측정 및 효용측정(EQ-5D, EQ-VAS)을 실시한다.
- 다음 방문일을 교육한다.
- After the screening visit, participants will be assigned a randomization number, and the following assessments will be conducted.
- A subject identification code will be assigned based on randomization.
- Changes in the participant's medical history or treatment history will be reviewed.
- Any changes in concomitant medications will be reviewed.
- Vital signs (blood pressure, pulse, temperature, respiratory rate) will be measured.
- Before the intervention, the following evaluations will be conducted:
- TMJ pain VAS
  a. Average pain over the past week
  b. Worst pain over the past week
- Vertical mandibular opening
  a. Pain-free opening
  b. Maximum unassisted opening
- JFLS-8, Chronic Pain Grade Scale 2.0, Treatment Expectancy Scale
- The TBT group will receive TBT treatment; the control group will receive TENS treatment.
- TMJ disorder education will be provided to all participants regardless of group allocation, including educational materials on:
- Causes, prevention, treatment and management strategies for TMJ disorders
- Self-correction techniques and stretching exercises
- Adverse events will be checked after the intervention.
- PGIC, cost measurement, and utility assessment (EQ-5D, EQ-VAS) will be conducted post-intervention.
- The next visit date will be scheduled and explained.

11.2.3.2 방문 2~11(0~5주) (Visits 2–11 (Weeks 0–5))

- 시험대상자의 병력이나 치료력의 변화여부를 확인한다.
- 병용약물 변화여부를 확인한다.
- 활력징후를 측정한다.
- 이상반응을 확인한다.
- 시험대상자의 병력이나 치료약물의 변화여부를 확인하여 기록한다.
- 시험군은 TBT 치료를 실시하며, 대조군은 TENS 치료를 실시한다.
- 중재 시술 이후 효용측정(EQ-5D, EQ-VAS)을 실시한다.
- 해당 방문까지의 순응도(예상되는 최대 중재횟수를 12로 나눈 값에 해당)를 확인한다.
- 다음 방문일을 지정한다(단, 계획된 방문일로부터 계산한다).
- Changes in medical history or treatment history will be reviewed.
- Changes in concomitant medications will be reviewed.

- Vital signs will be measured.
- Adverse events will be assessed.
- Any changes in treatment history or medications will be documented.
- The TBT group will receive TBT; the control group will receive TENS.
- Utility assessments (EQ-5D, EQ-VAS) will be conducted after each intervention.
- Compliance up to the current visit will be assessed based on the number of completed sessions divided by the total of 12.
- The next visit will be scheduled based on the planned schedule.

11.2.3.3 방문 12(6주) (Visit 12 (Week 6))

- 시험대상자의 병력이나 치료력의 변화여부를 확인한다.
- 병용약물 변화여부를 확인한다.
- 활력징후를 측정한다.
- 이상반응을 확인한다.
- 시험대상자의 병력이나 치료약물의 변화여부를 확인하여 기록한다.
- 시험군은 TBT 치료를 실시하며, 대조군은 TENS 치료를 실시한다.
- 중재 시술 후 턱관절 통증 VAS(a. 지난 1주간의 평균적인 턱관절 통증, b. 지난 1주간의 가장 심했던 턱관절 통증), 수직 턱관절 개구도(a. Pain free opening, b. Maximum unassisted opening), JFLS-8, 만성통증 척도 2.0, 치료 기대 척도 평가를 실시한다.
- PGIC, 비용측정(의료비용, 생산성손실비용) 및 효용측정(EQ-5D, EQ-VAS)을 실시한다.
- 해당 방문까지의 순응도(예상되는 최대 중재횟수를 12로 나눈 값에 해당)를 확인한다.
- 다음 방문일을 지정한다(단, 계획된 방문일로부터 계산한다).
- Review of medical and treatment history changes
- Review of concomitant medication changes
- Vital signs measurement
- Adverse event monitoring
- Documentation of any changes in medical history or medications
- TBT treatment for the treatment group, TENS treatment for the control group
- Post-treatment evaluations:
- TMJ pain VAS (a. average over the past week, b. worst over the past week)
- Vertical mandibular opening (a. pain-free opening, b. maximum unassisted opening)
- JFLS-8, Chronic Pain Grade Scale 2.0, Treatment Expectancy Scale
- PGIC, cost measurement (medical and productivity loss costs), and utility assessments (EQ-5D, EQ-VAS)
- Compliance will be reassessed
- The next visit will be scheduled based on the protocol

11.2.3.5 방문 13(10주) (Visit 13 (Week 10))

- 시험대상자의 병력이나 치료력의 변화여부를 확인한다.
- 병용약물 변화여부를 확인한다.
- 활력징후를 측정한다.
- 이상반응을 확인한다.
- 시험대상자의 병력이나 치료약물의 변화여부를 확인하여 기록한다.
- 턱관절 통증 VAS(a. 지난 1주간의 평균적인 턱관절 통증, b. 지난 1주간의 가장 심했던 턱관절 통증), 수직 턱관절 개구도(a. Pain free opening, b. Maximum unassisted opening), JFLS-8, 만성통증 척도 2.0, 치료 기대 척도 평가를 실시한다.
- PGIC, 비용측정(의료비용, 생산성손실비용) 및 효용측정(EQ-5D, EQ-VAS)을 실시한다.
- 해당 방문까지의 순응도를 확인한다.
- Review of medical and treatment history changes
- Review of concomitant medication changes
- Vital signs measurement
- Adverse event monitoring
- Documentation of any changes in medical history or medications

- Final evaluations:
- TMJ pain VAS (a. average over the past week, b. worst over the past week)
- Vertical mandibular opening (a. pain-free opening, b. maximum unassisted opening)
- JFLS-8, Chronic Pain Grade Scale 2.0, Treatment Expectancy Scale
- PGIC, cost measurement (medical and productivity loss costs), and utility assessments (EQ-5D, EQ-VAS)
- Final compliance will be recorded

11.2.3.9 추가방문 (Unscheduled Visits)

- 추가방문은 예정된 방문 외에 시험대상자 요청 또는 시험자의 판단에 의해 필요하다고 판단될 때 수시로 이루어질 수 있다. 시험대상자가 예정되지 않은 날에 방문한 경우에는 이상반응, 병용약물, 시행한 검사의 결과 및 그에 따른 의학적 처치 등을 증례기록지에 기록하여야 한다. 중도탈락된 시험대상자의 경우라도 추가방문을 실시하면 이학적인 검진, 면담, 유효성평가 검사를 시행할 수 있다.
- Unscheduled visits may occur as needed based on participant request or investigator judgment. For unscheduled visits, any findings (e.g., adverse events, concomitant medication changes, test results, and medical management) must be recorded in the Case Report Form (CRF). If a participant who had dropped out returns for an unscheduled visit, physical examination, interview, and efficacy assessments may still be conducted.

12. 예측 이상반응 및 사용상의 주의사항 (Expected Adverse Events and Precautions for Use)

- TBT 중재와 관련하여 발생 가능한 adverse event의 세부 종류는 다음과 같다. (The following are the detailed types of adverse events that may occur in association with TBT)
  - 턱관절 균형요법 및 경추 추나로 인하여 신체의 관절과 근육이 점차 정상적인 위치를 찾아감에 따라 몸의 균형점 변화로 인하여 근육통이나 관절통, 두통 등이 일시적으로 발생할 수 있으나 대부분 며칠 내 소실된다. (As the joints and muscles gradually return to their normal positions through TMJ balancing and cervical Chuna therapy, changes in the body's equilibrium may cause transient muscle pain, joint pain, or headaches. These symptoms are typically mild and resolve within a few days.)
- TENS 중재와 관련하여 발생 가능한 adverse event의 세부 종류는 다음과 같다. (The following are the detailed types of adverse events that may occur in association with TENS)
  - 신체 내 전기기기, pacemaker 등을 착용한 사람은 제외기준에 해당한다. 전기자극에 민감한 환자는 주의가 필요하며, 치료 중 통증 및 불편감이 발생할 수 있다. 전극 pad가 접촉되는 부위에 발진이나 알레르기 반응이 발생할 수 있다. (Individuals with implanted electrical devices (e.g., pacemakers) are excluded from participation. Patients who are sensitive to electrical stimulation should be treated with caution, as pain or discomfort may occur during treatment. Skin rashes or allergic reactions may occur at the electrode pad contact sites.)
- 상기에 언급된 예측된 이상반응 외에 새롭게 나타날 가능성을 배제할 수 없으며 본 임상시험 중 나타나는 이상반응에 대해서는 해당 기관의 연구의사가 적절한 조치를 취할 것이다. 또한, 수집되는 이상반응은 대상자 또는 대상자의 법정대리인에게 즉시 정보를 제공할 예정이다.

(Apart from the above-mentioned anticipated adverse events, there is the possibility of unexpected new adverse events arising during the clinical trial. If such events occur, the study physician at the investigational site will take appropriate action. Additionally, any adverse events that are identified will be promptly communicated to the participant or their legally authorized representative.)

13. 중지 및 탈락 기준 (Discontinuation and Withdrawal Criteria)

13.1 중지의 정의 (Definition of Discontinuation)

- 부작용, 이상사례 발생 등으로 인하여 임상시험을 진행할 수 없거나 임상시험의 진행이 대상자의 안전보호를 위협하여 그 진행을 멈추는 것으로 정의한다. (Discontinuation is defined as the suspension of the clinical trial due to adverse events or other safety-related concerns, which prevent the continuation of the study or pose a threat to the participant's well-being.)

13.2 중지의 기준 (Criteria for Discontinuation)

1) 대상자의 안전과 임상시험의 진행에 심각한 영향을 줄 수 있는 이상반응이 발생한 경우 (Occurrence of adverse events that significantly affect participant safety or the progress of the clinical trial)
2) 기타 시험자의 판단에 의해 시험 진행이 적합하지 못하다고 판단되는 경우 (Any other situation in which the investigator deems continuation of the study to be inappropriate)
   - 임상시험 진행 중 시험대상자의 안전과 임상시험의 심각한 영향을 줄 수 있는 이상반응이 발생할 경우, 그 시점에서 진행 중인 시험대상자의 시험은 가능한 한 종료하고, 추가 시험대상자를 대상으로 한 시험 역시 일단 중지한다. 다만 임상시험계획서에 명시된 안전성 평가는 그대로 시행한다. 중재 방법의 안전성에 대해 임상시험책임자와 임상시험의뢰자가 논의하여 임상시험의 진행 또는 중단 여부를 결정하고, 결정사항에 대하여 IRB에 보고한다. (If a serious adverse event affecting the participant's safety or the overall study arises during the trial, the ongoing treatment for current participants should be terminated if necessary, and recruitment or treatment of new participants will be suspended. However, safety assessments as outlined in the protocol will continue. The principal investigator and the sponsor will jointly review the safety of the intervention and determine whether to continue or terminate the study, and report the decision to the IRB.)

13.3 탈락의 정의 (Definition of Withdrawal)

- 계획서에 명시되어 있는 대로 치료시행과 그 전후의 관찰과정을 완료한 경우 시험대상자가 시험을 완료한 것으로 정의한다. 이상반응 또는 다른 이유로 인해 시험을 완료하지 못한 경우 시험대상자가 탈락된 것으로 본다. 시험대상자의 시험탈락은 시험 기간 중 어느 시점에서나 판정 가능하며, 탈락 시에는 시험대상자와 시험책임자에게 보고하도록 한다. (Participants who complete the intervention and all pre- and post-treatment assessments according to the protocol are considered to have completed the study. Participants who fail to complete the

study due to adverse events or other reasons are considered withdrawn. A subject may be withdrawn at any time during the study, and such withdrawal must be reported to both the participant and the principal investigator.)

13.4 탈락의 기준 (Criteria for Discontinuing)

- 시험자는 치료와 관찰을 중단시키고 시험대상자를 탈락시킬 수 있으며, 시험대상자는 언제나 시험에서 자유의사로 탈락할 수 있다. (The investigator may discontinue treatment and withdraw the subject from the trial, and participants may withdraw from the study at any time of their own free will.)
- 탈락의 기준은 다음과 같다. (The criteria for withdrawal include:)

1) 시험대상자에게 중대한 이상반응이 발생한 경우 혹은 이상반응으로 인하여 시험 진행이 어려운 경우 (A serious adverse event occurs, or an adverse event makes continuation of the trial difficult)
2) 투여 전 검사에서 발견치 못한 전신질환이 발견된 경우 (A previously undetected systemic disease is identified before or during the trial)
3) 임상시험 기간 중 만족스럽지 못한 효과로 인하여 시험대상자 또는 시험대상자의 법정 대리인이 시험 중단을 요구하는 경우 (The participant or legal guardian requests to discontinue due to unsatisfactory results)
4) 시험대상자가 시험자의 지시에 불응하는 경우 (The participant fails to follow the investigator’s instructions)
5) 시험대상자가 임상시험 참가 동의를 철회한 경우 (The participant withdraws informed consent)
6) 시험대상자의 추적이 안 되는 경우 (The participant becomes lost to follow-up)
7) 시험 기간 또는 경과 관찰 기간 동안 연구의사의 지시나 동의 없이 시험결과 판정에 영향을 미칠 수 있는 약물 등을 복용한 경우 (The participant takes prohibited medication or undergoes other treatments without approval during the study period that may affect the trial outcome)
8) 기타 연구의사의 판단에 의해 시험의 진행이 적합하지 못하다고 판단되는 경우 (Any other circumstance in which the investigator determines continuation is inappropriate)

13.5 순응도와 임상시험 계획서 위반에 대한 처리 (Handling of Compliance and Protocol Deviations)

- 임상시험 책임자와 담당자는 임상시험 계획서의 위반이 발생하지 않도록 계획서에 대해 충분히 숙지하고 철저히 이행한다. 시험담당자는 대상자의 방문, 검사 일정 준수를 위하여 적절한 조치, 예를 들어 휴대폰 문자메시지, 전화 모니터링, 서면 통보, 이메일 등을 실시한다. 부득이하게 발생된 계획서 위반사항에 대해서는 다음에 따라 처리한다. (The principal investigator and clinical trial staff must thoroughly understand and comply with the study protocol to prevent any violations. The study team will support compliance through measures such as SMS reminders, phone calls, letters, or emails to ensure visit and assessment schedules are followed. If protocol violations occur, the following procedures apply:)

- 중대한 임상시험계획서 위반의 경우 예를 들어 선정/제외기준 위반, 동의서 미취득, 임상시험 결과에 영향을 미칠 수 있는 약물 등을 복용한 경우 등 시험대상자의 안전과 시험결과에 중대한 영향을 미칠 수 있는 위반의 경우, 해당 시험대상자의 정보는 분석에서 제외함을 원칙으로 한다. (Major protocol violations (e.g., failure to meet inclusion/exclusion criteria, lack of informed consent, use of prohibited medications that may affect trial outcomes) will result in exclusion of the subject's data from analysis.)
- 기타 경미한 위반사항은 위반 또는 지연 정도와 사유를 정확히 기재하여 분석할 때 임상시험 결과에 영향을 주었는지 평가한다. (Minor violations will be evaluated based on their degree and reason, and it will be assessed whether they affected the study outcome.)

13.6 중지 및 탈락의 처리 과정 (Procedures for Discontinuation and Withdrawal)

- 시험대상자가 탈락 및 중지한 날짜와 시간, 사유를 종료보고서에 기록한다. 어떠한 사유든지 탈락 및 중지한 시험대상자를 추적 관찰하기 위한 모든 노력을 기울여야 하며 안전성에 대한 추적관찰 평가를 탈락 및 중지 후 1주일 이내에 수행하여야 한다. 시험대상자는 어떠한 사유든지 언제나 시험에서 자유의사로 탈락할 수 있으며 탈락 사유에 대하여 진술할 의무는 없다. 그러나 시험자는 탈락 및 중지한 사유 또는 사유 파악의 실패 등을 종료보고서에 기록하여야 하며 탈락한 시험대상자를 추적 관찰하는 데 모든 노력을 기울여야 한다. 시험자는 시험대상자를 시험에서 제외시킬 수 있는 선택권 또한 가지고 있다. 중지 및 탈락한 시험대상자는 새로운 시험대상자로 대체되지 않는다. (The date, time, and reason for withdrawal or discontinuation will be recorded in the termination report. Every effort should be made to follow up with withdrawn or discontinued subjects, and safety follow-up evaluations must be conducted within one week of withdrawal. Participants have the right to withdraw from the study at any time for any reason and are not obligated to provide a reason. However, the investigator must document the reason for withdrawal or the failure to determine the reason in the termination report, and make all efforts to follow up. The investigator also reserves the right to withdraw subjects from the study. Withdrawn or discontinued participants will not be replaced by new subjects.)
- 임상시험 참여 중 발생한 부작용 등의 사유로 인해 연구 참여를 중단하는 경우에는 예측하지 못한 추가 비용의 발생이 가능하므로, 해당 비용 발생 항목 및 금액에 대해 수집하기 위하여 경제성평가 증례기록 설문을 시행할 수 있다. (If a participant discontinues the study due to adverse events or related issues, unanticipated costs may occur. In such cases, a cost-related economic evaluation questionnaire may be administered to collect data on the incurred expenses.)

13.7 임상시험의 조기 종결 (Early Termination of the Clinical Trial)

- 시험 기간 중에 시험자, 모니터요원, 해당 정부기관에서 본 임상시험을 중지하여야 하는 상황이 발생했을 때 임상시험 책임자는 본 시험을 조기 종결할 수 있다. (The study may be terminated early if necessary, based on the judgment of the investigator, monitor, or regulatory authorities. In such cases, the principal investigator has the authority to terminate

the study prematurely.)

14. 평가기준, 평가방법 및 통계분석방법(Evaluation criteria, evaluation methods, and statistical analysis methods)

14.1. 평가기준(Evaluation criteria)

14.1.1 일차 유효성 평가변수(Primary efficacy evaluation variable)

1) 턱관절 통증 VAS(100mm): 연구 시작 전(baseline)과 6주 후 VAS 점수의 변화 (Temporomandibular joint (TMJ) pain VAS (100 mm): Change in VAS scores before the start of the study (baseline) and 6 weeks later)
   a. 지난 1주간의 평균적인 턱관절 통증(Average TMJ pain over the past week)

14.1.2 이차 유효성 평가변수

1) 턱관절 통증 VAS(100mm): 연구 시작 전과 6주, 10주 후 VAS 점수의 변화(Temporomandibular joint (TMJ) pain VAS (100 mm): Change in VAS scores before the start of the study and at 6 and 10 weeks)
   a. 지난 1주간의 평균적인 턱관절 통증(Average TMJ pain over the past week)
   b. 지난 1주간의 가장 심했던 턱관절 통증(The worst TMJ pain experienced over the past week)

2) 수직 턱관절 개구도: 시험 시작 전(baseline)과 6주, 10주 후 턱관절 개구도의 변화(Vertical mouth opening: Change in vertical mouth opening before the start of the trial (baseline) and at 6 and 10 weeks)
   a. Pain free opening
   b. Maximum unassisted opening

3) 턱기능 제한지수(JFLS-8): 시험 시작 전(baseline)과 6주, 10주 후 턱기능 제한지수 점수의 변화 (Jaw Functional Limitation Scale (JFLS-8): Change in JFLS-8 scores before the start of the trial (baseline) and at 6 and 10 weeks)
4) DC/TMD 만성통증 등급 척도 2판(만성통증 척도 2.0): 시험 시작 전(baseline)과 6주, 10주 후 만성통증 등급 척도 점수의 변화(DC/TMD Chronic Pain Grading Scale, Version 2 (Chronic Pain Scale 2.0): Change in chronic pain grading scale scores before the start of the trial (baseline) and at 6 and 10 weeks)
5) 치료 기대 척도: 시험 시작 전(baseline)과 6주, 10주 후 치료 기대 척도 점수의 변화(Treatment Expectation Scale: Change in treatment expectation scale scores before the start of the trial (baseline) and at 6 and 10 weeks)
6) 전반적 평가 척도(PGIC): 시험 시작 후(baseline)과 6주, 10주 후 전반적 평가 척도 점수의 변화치의 변화(Patient Global Impression of Change (PGIC): Change in PGIC scores before the start of the trial (baseline) and at 6 and 10 weeks)

14.1.3 안전성 평가변수(Safety evaluation variable)

• 이상반응 평가(Adverse event assessment)

- 이상반응의 유무 및 TBT(대조군의 경우 TENS)와의 관련 여부를 평가(Evaluate the presence of adverse events and their relationship to TBT (or TENS in the control group)

• 활력징후 측정을 통해 중재 방법의 안전성을 평가(Assess the safety of the intervention method by measuring vital signs)

• 면담을 통해 중재 이후 불편감 및 중재 방법의 안전성을 평가(Assess discomfort after the intervention and the safety of the intervention method through interviews)

• 이상반응을 포함한 안전성의 평가 기준 및 평가방법(Criteria and methods for safety assessment, including adverse events)

- 부작용, 활력징후를 기록(Record adverse events and vital signs)
- 안전성 평가 대상: 시험에 참여한 전례 (ITT군)에 대하여 평가(Safety assessment population: All subjects who participated in the trial (ITT group))
- 임상시험 시작 전에 관찰되지 않은 증상이 발생하는 모든 바람직하지 않은 의학적 소견을 이상반응으로 분류(Any undesirable medical findings that were not observed before the start of the clinical trial are classified as adverse events)

• 시험 기간 중 발생할 수 있는 이상 증상에 대한 대비(Preparedness for possible adverse symptoms during the trial period)

- 임상시험 시작 전에 관찰되지 않은 증상이 발생하면 이에 대한 의학적 조치를 제시(If symptoms not observed before the start of the clinical trial occur, provide appropriate medical measures)

14.1.4 경제성 평가변수(Economic evaluation variable)

• 주 경제성평가지표(Primary economic endpoint): cost per QALY(Quality Adjusted Life Years) gained

- QALYs: QALY 산출을 위한 삶의 질 추정은 EQ-5D로 도출된 삶의 질을 주 평가변수로 사용한다.(For estimating quality of life for QALY calculation, the quality of life derived from the EQ-5D is used as the primary evaluation variable.) (Area under the curve method)
- Cost: 임상시험과 관련하여 발생하는 치료비용은 치료 횟수와 단위비용을 결합하여 산출하며, 임상시험 기관 내에서 발생하는 치료비용은 맹검 해제 후 시험자가 전산 자료를 조사하여 기록한다.(Treatment costs related to the clinical trial are calculated by combining the number of treatments with the unit cost. Treatment costs incurred within the clinical trial site are recorded by the investigator after unblinding by reviewing electronic data.)

• 이차평가지표(Secondary outcome measures): Cost per EQ-VAS

14.2 통계분석의 일반적 원칙(General principles of statistical analysis)

14.2.1 분석군

1) 전체 분석군(Full Analysis Set, FAS)

• ITT 분석 원칙에 가능한 부합하게 분석 대상군을 정한다. 본 시험에서는 아래의 제외기준에 해당하는 대상자를 제외한 모든 대상자에 대하여 무작위 배정된 대로 시험결과를 분석한다. 다음에 해당하는 시험대상자는 FAS 분석에서 제외한다.(The analysis set is determined to be as consistent as possible with the principles of ITT (intention-to-treat) analysis. In this trial, all participants, except those who meet the exclusion criteria below, are analyzed according to the group to which they were randomized. The following participants are excluded from the FAS (full analysis set) analysis.)

- 주요 선정기준을 만족하지 못한 경우(If participants do not meet the main inclusion criteria)
- 임상시험계획서에 명시된 중재를 한 번도 받지 않은 경우(If participants did not receive any intervention specified in the clinical trial protocol)
- 무작위 배정 이후 한 번도 평가되지 않아 어떠한 자료도 수집하지 못한 경우(If participants were not assessed at all after randomization, resulting in no data being collected)

2) 계획서 순응 분석군(Per-Protocol, PP)

• 계획서에 명시되어 있는 대로 시험 시작 후 임상시험 계획서 위배 없이 시험의 전 과정을 완료한 시험대상자를 대상으로 분석하며, 다음에 해당하는 시험대상자는 PP분석에서 제외한다.(Analysis is performed on participants who completed the entire trial process without any violations of the clinical trial protocol as specified in the protocol, starting from the beginning of the trial. The following participants are excluded from the per-protocol (PP) analysis)

- 중재 기간 동안 중도 탈락한 대상자(Participants who dropped out during the intervention period)
- 선정/제외 기준을 위반한 대상자(Participants who violated the inclusion/exclusion criteria)
- 전체 중재 시술 순응도가 66.6% 미만인 대상자(Participants with overall intervention procedure compliance of less than 66.6%)
- 그 외 중대한 임상시험계획서 위반으로 간주할 수 있는 경우(Other cases considered to be a major violation of the clinical trial protocol)

14.2.2 자료분석의 일반적 원칙(General Principles of Data Analysis)

• 중재방법의 유효성은 1차 평가변수의 FAS 분석 결과에 근거하여 평가하며, PP 분석군의 결과는 보조적으로 활용한다. 결측이 발생한 경우 결측량과 기전에 대한 진단을 먼저 시행한 후 적합한 imputation 방법을 선정하여 처리한다. 이 방법은 시험결과를 보수적으로 평가할 것으로 기대된다. 통계적 검정은 양측검정을 원칙으로 하며 유의수준은 5%로 한다.(The efficacy of the intervention is evaluated based on the results of the primary outcome variable from the FAS (full analysis set) analysis, and the results from the PP (per protocol) analysis set are used as supplementary evidence. When missing data occur, the extent and mechanism of the missing data are first assessed, and then an appropriate imputation method is selected and applied. This approach is expected to provide a conservative evaluation of the trial results. Statistical testing is performed as a two-sided test by default, with the significance level set at 5%.)

14.3 인구학통계학적 자료와 시험 시작 시 임상병력 자료의 분석

(Analysis of demographic and baseline clinical history data)

• 본 임상시험에 포함된 시험대상자의 인구통계학적 자료 및 시험 시작 시 임상병력 자료(이하 기초자료)들은 각 시험군 별로 연속형 자료는 평균(mean), 표준편차(SD) 등을 제시하고 범주형 자료는 빈도(frequency), 백분율(percentile)을 제시한다. 처리군 별로 인구통계학적 자료와 기초자료의 비교 평가방법은 연속형 변수의 경우, 정규성 여부에 따라 Student`s independent sample t-test 또는 Wilcoxon rank sum test를 시행하고 범주형 변수의 경우, Pearson chi-squared test 또는 Fisher's exact test를 시행한다.(For the demographic data and baseline clinical history data (hereinafter referred to as baseline data) of participants included in this clinical trial, continuous data are presented as mean and standard deviation (SD) for each treatment group, and categorical data are presented as frequency and percentage. To compare demographic and baseline data between treatment groups, for continuous variables, either Student's independent sample t-test or the Wilcoxon rank sum test is performed depending on the normality of the data; for categorical variables, either the Pearson chi-squared test or Fisher's exact test is performed.)

14.4 유효성 평가변수의 분석방법(Analysis Methods for Efficacy Evaluation Variables)

14.4.1 일차 유효성 평가변수의 분석(Analysis of Primary Efficacy Evaluation Variables)

1) 턱관절 통증 VAS(100mm): 연구 시작 전과 6주 후 VAS 점수의 변화
- a. 지난 1주간의 평균적인 턱관절 통증
- 시험군과 대조군 간의 평균 차이의 추정값과 95% 신뢰구간, 그리고 p-value 값을 제시한다.
- 무작위 배정을 받은 시험대상자 중 적어도 한 번 이상 중재를 받고 시험 전과 중재 시술 이후 적어도 한 번 이상 VAS 점수가 측정된 대상자를 분석군에 포함한다. VAS 점수의 결측이 발생한 경우, 결측량과 기전에 대한 진단을 먼저 시행한 후 적합한 imputation 방법을 선정하여 FAS 분석을 시행한다.
- VAS 점수의 변화에 대한 두 군의 차이를 independent t test를 통해 검증한다. 기저 값의 유의한 차이가 있는 경우는 이를 보정한 공분산 분석을 시행하고, 기타 기저 변수의 유의한 차이를 보정하기 위해서는 다중회귀분석을 시행한다.
- VAS 점수에 대한 반복 측정된 값을 대상으로 반복측정분산분석을 통해 시간과 치료간의 교호작용을 검정한다.

1) Temporomandibular joint (TMJ) pain VAS (100 mm): Change in VAS scores before the start of the study and at 6 weeks
- a. Average TMJ pain over the past week
- The estimated mean difference between the intervention and control groups, the 95% confidence interval, and the p-value are presented.
- The analysis set includes participants who were randomized, received at least one intervention, and had VAS scores measured at least once before the intervention and at

least once after the intervention procedure. If missing VAS scores occur, the extent and mechanism of missingness are first assessed, and then an appropriate imputation method is selected to conduct the FAS (full analysis set) analysis.

- The difference in VAS score changes between the two groups is tested using an independent t-test. If there is a significant difference in baseline values, analysis of covariance (ANCOVA) is performed to adjust for this. To adjust for other significant baseline differences, multiple regression analysis is conducted.
- For repeated VAS measurements, the interaction between time and treatment is tested using repeated measures analysis of variance (ANOVA)

14.4.2 이차 유효성 평가변수의 분석(Analysis of Secondary Efficacy Evaluation Variables)

1) 턱관절 통증 VAS(100mm): 연구 시작 전과 6주, 10주 후 VAS 점수의 변화 (a. 지난 1주간의 평균적인 턱관절 통증, b. 지난 1주간의 가장 심했던 턱관절 통증을 각각 분석.
2) 수직 턱관절 개구도: 시험 시작 전(baseline)과 6주, 10주 후 턱관절 개구도의 변화 (a. Pain free opening, b. Maximum unassisted opening을 각각 분석)
3) 턱기능 제한지수(JFLS-8): 시험 시작 전(baseline)과 6주, 10주 후 턱기능 제한지수 점수의 변화
4) DC/TMD 만성통증 등급 척도 2판: 시험 시작 전(baseline)과 6주, 10주 후 만성통증 등급 척도 점수의 변화
5) 치료 기대 척도: 시험 시작 전(baseline)과 6주, 10주 후 치료 기대 척도 점수의 변화
6) 전반적 평가 척도(PGIC): 시험 시작 전(baseline)과 6주, 10주 후 전반적 평가 척도 점수의 변화치의 변화

- 턱관절 통증 VAS, 수직 턱관절 개구도, 턱기능 제한지수(JFLS-8), DC/TMD 만성통증 등급 척도 2판, 치료 기대 척도, 전반적 평가 척도(PGIC)는 Shapiro-Wilk 또는 Kolmogorov-Smirnov Test 등을 활용하여 데이터 정규성을 확인하고, 정규성이 만족되는 경우 Repeated Measures ANOVA 등을, 정규성이 만족되지 않는 경우 Linear Mixed Model 등을 활용하여 분석한다. 이때, 시험 시작 전 점수가 측정된 경우는 이를 공변량으로 하며, 결측치에 대한 별도의 처리는 하지 않는 per-protocol 분석을 시행한다.

1) TMJ Pain VAS (100 mm): Change in VAS scores before the start of the study and at 6 and 10 weeks (a. Average TMJ pain over the past week, b. Worst TMJ pain over the past week, each analyzed separately).
2) Vertical TMJ Opening: Change in TMJ opening before the start of the trial (baseline) and at 6 and 10 weeks (a. Pain-free opening, b. Maximum unassisted opening, each analyzed separately).
3) Jaw Functional Limitation Scale (JFLS-8): Change in JFLS-8 scores before the start of the trial (baseline) and at 6 and 10 weeks.
4) DC/TMD Chronic Pain Grading Scale, Version 2.0: Change in chronic pain grading scale scores before the start of the trial (baseline) and at 6 and 10 weeks.
5) Treatment Expectation Scale: Change in treatment expectation scale scores before the start

of the trial (baseline) and at 6 and 10 weeks.
6) Patient Global Impression of Change (PGIC): Change in PGIC scores before the start of the trial (baseline) and at 6 and 10 weeks.

- For TMJ pain VAS, vertical TMJ opening, JFLS-8, DC/TMD chronic pain grading scale (version 2.0), treatment expectation scale, and PGIC, data normality is checked using the Shapiro-Wilk or Kolmogorov-Smirnov test, etc. If normality is satisfied, Repeated Measures ANOVA, etc. is used for analysis; if normality is not satisfied, Linear Mixed Model, etc. is used for analysis. In this case, if scores from before the start of the trial are available, they are included as covariates, and per-protocol analysis is performed without any special handling of missing data.

14.4.3 경제성 평가 분석(Economic Evaluation Analysis)

- 본 연구에서 수집된 비용 및 효과 자료를 바탕으로 비용-효과 분석(cost-effectiveness analysis)을 실시하여 시험군과 대조군 간 비용-효과성을 비교한다.(Based on the cost and effectiveness data collected in this study, a cost-effectiveness analysis will be conducted to compare the cost-effectiveness between the intervention and control groups.)
  - 분석 기간: 10주(본 연구의 추적 관찰 기간)(Analysis period: 10 weeks (the follow-up period of this study))
  - 모형(Model): Decision tree model (결정수형모형, Decision tree model)
  - 분석 관점: 보건의료체계 관점 및 사회적 관점(Analysis perspectives: Healthcare system perspective and societal perspective)
    ① 보건의료체계 관점: 비용 평가 시 의료비용을 포함(Healthcare system perspective: Includes medical costs in cost evaluation)
    ② 사회적 관점: 비용 평가 시 의료비용, 비의료비용, 생산성손실비용을 포함(Societal perspective: Includes medical costs, non-medical costs, and productivity loss costs in cost evaluation)
  - 할인율: 분석 기간이 1년 미만이므로 할인율 미적용(Discount rate: No discount rate applied as the analysis period is less than one year)

경제성평가분석은 FAS 분석을 원칙으로 하며 결측 분석 결과에 대한 민감도를 확인하기 위한 PP 분석을 같이 시행한다. 경제성평가의 비용 및 효과(Utility) 추정은 임상시험의 효과추정과 동일한 방식으로 missing data에 대한 기전 등을 분석한 후 적합한 imputation 방식을 선택하여 시행한다.(Economic evaluation analysis is primarily based on FAS analysis, and PP analysis is also conducted to check sensitivity to missing data analysis results. Cost and effectiveness (Utility) estimation for economic evaluation is performed by analyzing the mechanism of missing data in the same way as the clinical trial effect estimation and then selecting an appropriate imputation method.)

- 기본 분석 기간은 10주로 시행하며, 이후의 기간에 대한 추정이 필요할 경우 Markov model 등을 이용하여 장기 비용과 효과를 모델링한다. 총 분석 기간(Time Horizon)이 12개월 이상이 될 경우 비용단위는 2024년 대한민국 화폐단위(원)으로 통일하고, 건강보험심사평가원 경

제성평가 지침에 근거하여 4.5%의 할인율을 적용한다.(The basic analysis period is 10 weeks, and if estimation for the subsequent period is needed, long-term costs and effects are modeled using Markov models, etc. If the total analysis period (Time Horizon) is 12 months or longer, the cost unit is unified to the 2024 Korean currency unit (KRW), and a 4.5% discount rate is applied based on the Health Insurance Review & Assessment Service economic evaluation guidelines.)

- 효과 추정: 무작위배정 후 임상시험 일정표에 따라 측정한 질보정수명 및 개별 유효성 지표 결과를 바탕으로 분석한다.(Effect estimation: Analysis is based on quality-adjusted life years and individual efficacy indicator results measured according to the clinical trial schedule after randomization.)
  - 질보정수명: 타당도가 입증된 한국어 버전의 EQ-5D-5L을 사용하여 시험대상자의 삶의 질을 측정하고, Kim 등(2016)의 연구에서 개발한 한국인 대상 질가중치 점수체계(tariff)를 적용하여 시험대상자별 질보정수명(quality-adjusted life year, QALY) 산출(Quality-adjusted life years: Quality of life of subjects is measured using the validated Korean version of EQ-5D-5L, and quality-adjusted life years (QALY) for each subject are calculated by applying the Korean tariff developed by Kim et al. (2016).)
  - 개별 유효성 지표: 본 연구에서 설정한 일차 유효성 평가변수 및 이차 유효성 평가변수의 분석 결과를 바탕으로 시험대상자별 효과 크기 산출(Individual efficacy indicators: Effect sizes for each subject are calculated based on the analysis results of the primary and secondary efficacy evaluation variables set in this study.)
- 비용 추정: 비용 항목 수집 결과를 바탕으로 미시적 비용 산출방법(micro-costing appriach)을 활용하여 비용 항목별 단위비용 및 사용량을 결합하여 산출한다.(Cost estimation: Using the micro-costing approach based on the collected cost item data, unit costs and usage for each cost item are combined to calculate costs.)
  - 기관 자료: 임상시험기관에서 TBT 및 TENS 적용을 위해 소모되는 비용, 시간 등에 관한 자료로 기관에서 내부적으로 수집함(Institutional data: Data on costs and time consumed for applying TBT and TENS at the clinical trial institution, collected internally by the institution.)
  - 시험대상자 설문: 무작위배정 후 0주차, 6주차, 10주차에 시험대상자에게 설문을 시행하여 '지난 6주간 소요된 비용'을 수집함(Subject survey: Surveys are conducted with subjects at weeks 0, 6, and 10 after randomization to collect 'costs incurred over the past 6 weeks.')

  ※ 생산성손실비용(Productivity loss costs): WPAI:GH 2.0 도구는 '지난 7일 동안' 발생한 생산성 손실에 관한 설문조사이므로, 적절한 내삽법(interpolation)을 활용하여 6주간 발생한 생산성손실비용을 추계함(Since the WPAI:GH 2.0 tool surveys productivity loss over the past 7 days, appropriate interpolation is used to estimate productivity loss costs over 6 weeks.)
- 경제성 평가 결과 지표(Economic evaluation outcome indicators)
  - 점증적 비용효용비(incremental cost-utility ratio, ICUR): EQ-5D-5L 결과 및 비용 평가 결과

를 바탕으로 다음과 같이 산출함(Incremental cost-utility ratio (ICUR): Calculated based on EQ-5D-5L results and cost evaluation results as follows)

$$ICUR = \frac{전체비용_{CP적용군} - 전체비용_{일반치료군}}{질보정수명_{CP적용군} - 질보정수명_{일반치료군}}$$

- 점증적 비용효과비(incremental cost-effectiveness ratio, ICER): 개별 유효성 지표 및 비용 평가 결과를 바탕으로 다음과 같이 산출함(Incremental cost-effectiveness ratio (ICER): Calculated based on individual efficacy indicators and cost evaluation results as follows)

$$ICER = \frac{전체비용_{CP적용군} - 전체비용_{일반치료군}}{효과_{CP적용군} - 효과_{일반치료군}}$$

- 민감도 분석: 가능한 모든 모수들에 대하여 일원민감도분석(deterministic sensitivity analysis)을 시행하여 토네이도 다이어그램을 제시하고, 가능한 모든 모수들의 분포 및 대푯값을 사용하여 확률적 민감도분석(probabilistic sensitivity analysis)을 시행한다.(Sensitivity analysis: Deterministic sensitivity analysis is performed for all possible parameters, presenting a tornado diagram, and probabilistic sensitivity analysis is conducted using distributions and representative values of all possible parameters.)

14.5 안전성 변수의 분석방법(Analysis Methods for Safety Variables)

- 평가 검사의 결과치에 대해 시험 전과 비교하여 임상적으로 어떤 변화가 있었는지를 평가한다. 발현된 모든 이상반응은 자세한 설명과 함께 나열한다. 발현된 모든 이상반응은 자세한 설명과 함께 나열한다. 중대한 이상반응은 모두 서술적으로 기술된다. 이상반응은 환자의 증상 보고, 시험자의 관찰 등을 통해 수집된다. 시험약과 연관성이 있는 이상반응과 연관성이 없는 이상반응의 빈도를 기록하며, 기술통계량으로 제시한다.(The results of the evaluation tests are compared to those before the trial to assess whether there have been any clinically significant changes. All adverse events that occur are listed along with detailed descriptions. All serious adverse events are described narratively. Adverse events are collected through patient symptom reports and investigator observations. The frequencies of adverse events related and unrelated to the investigational drug are recorded and presented as descriptive statistics.)
- 이상반응의 빈도, 발현율, 각각의 목록, 상세한 발현시간, 심각한 정도 및 시험약물과의 인과관계 등을 제시하며, 필요한 경우 그래프 형태로 보고한다. 통계적 분석이 필요한 경우는 변수의 특성과 목적에 따라 paired t-test, Mcnemar test, ANOVA, t-test, chi-square test 혹은 Fisher's exact test 등을 실시한다.(The frequency, incidence, individual listings, detailed time of onset, severity, and causal relationship with the investigational drug of adverse events are presented, and reported graphically if necessary. If statistical analysis is required, paired t-test, McNemar test, ANOVA, t-test, chi-square test, or Fisher's exact test are performed according to the characteristics and purpose of the variable.)

15. 이상반응(Adverse Event/Adverse Experience, AE)

15.1 안전성 관련 용어의 정의(Definitions of safety-related terms)

1) 이상반응(Adverse Event/Adverse Experience, AE)

- 임상시험 중재를 받은 시험대상자에게 발생한 모든 유해하고 의도하지 않은 증후(sign), 증상(symptom) 또는 질병을 말하며, 해당 중재방법과 반드시 인과관계를 가져야 하는 것은 아니다.(It refers to any harmful and unintended sign, symptom, or disease that occurs in a trial participant who has received the clinical trial intervention. It is not necessarily required to have a causal relationship with the intervention method.)

2) 이상약물반응(Adverse Drug Reaction, ADR)

- 임상시험용 의약품의 임의 용량에서 발생한 모든 유해하고 의도하지 않은 반응으로서 임상시험용 의약품과의 인과관계를 부정할 수 없는 경우를 말한다. 본 임상시험에서는 의약품이 중재가 아니기 때문에 해당사항이 없다.(It refers to any harmful and unintended reaction that occurs at any dose of the investigational drug for which a causal relationship with the investigational drug cannot be denied. In this clinical trial, since the intervention is not a drug, this does not apply.)

3) 중대한 이상반응/이상약물반응(Serious AE/SAE)

- 임상시험 중재를 받으면서 발생한 이상반응 또는 이상약물반응 중에서 다음의 어느 하나에 해당하는 경우를 말한다.(It refers to any adverse event or adverse drug reaction that occurs while a participant is receiving a clinical trial intervention and meets any one of the following criteria)

① 사망하거나 생명에 대한 위험이 발생한 경우(Results in death or poses a risk to life)
② 입원할 필요가 있거나 입원 기간을 연장할 필요가 있는 경우(Requires hospitalization or prolongation of existing hospitalization)
③ 영구적이거나 중대한 장애 및 기능 저하를 가져온 경우(Causes permanent or significant disability or functional impairment)
④ 태아에게 기형 또는 이상이 발생한 경우(Results in a congenital anomaly or abnormality in the fetus)

4) 예상하지 못한 이상약물반응(Unexpected ADR)

- 임상시험자 자료집 또는 의약품의 첨부 문서 등 이용 가능한 의약품 관련 정보에 비추어 이상약물반응의 양상이나 위해의 정도에서 차이가 나는 것을 말한다. 본 임상시험에서는 의약품이 중재가 아니기 때문에 해당사항이 없다.(It refers to any adverse drug reaction whose nature or severity is inconsistent with the available drug-related information, such as the investigator's brochure or the drug's accompanying documentation. In this clinical trial, since the intervention is not a drug, this does not apply.)

15.2 이상반응의 평가(Assessment of Adverse Events)

15.2.1 안전성의 평가(Safety Assessment)

- 부작용, 활력징후, 면담을 수행하고 내용을 기록한다.(Adverse events, vital signs, and interviews

are conducted, and their contents are recorded.)

- 본 임상시험의 중재를 1회 이상 받은 모든 시험대상자를 안전성 평가 대상으로 한다.(All participants who have received the intervention at least once in this clinical trial are included as the safety assessment population.)

15.2.2 이상반응의 평가 원칙(Principles of Adverse Event Evaluation)

- 이상반응은 중재를 받기 전에 관찰되지 않은 증상이 중재 기간 중에 새로 나타난 증상으로서 중재 방법과의 인과관계에 상관없이 의도하지 않았던 증후 및 증상과 약물 사용과 관련된 일시적인 현상 등을 총칭한다.(An adverse event refers to any symptom that was not observed before the intervention but newly appears during the intervention period, encompassing unintended signs and symptoms regardless of causal relationship with the intervention method, as well as temporary phenomena related to drug use.)
- 중재 방법의 이상반응으로 예상되는 현상(증상, 시작일, 지속기간 등)은 이상반응 보고서에 빠짐없이 기록하도록 한다.(Expected phenomena as adverse events of the intervention method (such as symptoms, onset date, duration, etc.) should be thoroughly recorded in the adverse event report.)
- 이상반응 정도에 대한 평가는 시험담당자가 평가 기준을 참고하여 증상의 경중에 따라 단계별로 평가하는 것을 원칙으로 한다.(The severity of adverse events should be evaluated by the investigator according to evaluation criteria, with a stepwise assessment based on the intensity of symptoms as a principle.)
- 중재 방법과의 인과관계는 시험담당자가 평가 기준에 따라 6단계로 분류하여 평가한다.(The causal relationship with the intervention method should be assessed by the investigator and classified into six levels according to the evaluation criteria.)

15.2.3 중증도 평가(Severity Assessment)

- 이상반응의 중증도는 최대강도(maximum intensity)에 의거하여 아래의 기준에 의하여 분류한다. 아래의 기준에 의하여 분류가 어려운 경우에 CTCAE Version 4.0을 참고할 수 있다.(The severity of adverse events is classified according to the following criteria based on the maximum intensity observed. If classification is difficult using the criteria below, the Common Terminology Criteria for Adverse Events (CTCAE) Version 4.0 may be referenced for detailed grading.)

1 = 경증<br>(Mild)	시험대상자가 거의 느끼지 못할 정도로 정상적인 일상생활(기능)을 저해하지 않는 정도, 대부분 치료가 필요하지 않은 정도<br>(The participant barely notices the symptoms, which do not interfere with normal daily activities (function). In most cases, no treatment is required.)
2 = 중등증<br>(Moderate)	시험대상자가 불편감을 느낄 수 있으며, 정상적인 일상적인(기능)을 저해하는 정도, 시험대상자가 시험을 계속할 수는 있으나 치료가 필요할 수도 있는 경우<br>(The participant may feel discomfort, and the symptoms interfere with normal daily activities (function). The participant may be able to continue the trial, but treatment may be required.)
3 = 중증<br>(Severe)	시험대상자가 매우 불편하여 일상생활(기능)이 불가능하고, 시험의 지속적인 참여가 불가능한 정도, 치료나 입원이 필요할 수 있는 정도<br>(The participant experiences significant discomfort, making daily activities (function) impossible and continuous participation in the trial unfeasible. Treatment or hospitalization may be required.)

15.2.4 시험치료법과의 인과관계 평가(Assessment of Causality with the Investigational Treatment)

- 임상시험 중재 방법과의 인과관계는 통계학적으로 검정할 수 없으므로 개인적 상황, 의학적(생리학적, 병리학적, 약리학적) 가능성, 문헌 등을 이용하여 판단하며, 시간적 관계를 고려하는 것도 도움이 된다. 또한, 처치 중지 및 재처치에 의한 소실, 재발현 여부, 병용요법 및 복용 약제 등을 고려한다.(Causality with the clinical trial intervention cannot be assessed statistically, so it is determined by considering individual circumstances, medical (physiological, pathological, pharmacological) plausibility, and literature. Temporal relationships should also be taken into account. Furthermore, the disappearance or recurrence of the event upon withdrawal or re-administration of the intervention, as well as concomitant therapies and medications, should be considered.)
- 이상반응과 임상시험 중재 방법과의 관계(또는 기타 원인 - 기저 질환의 진행, 병용 치료 등)의 확실성의 정도는 아래의 관점에서 이상반응이 얼마나 잘 설명될 수 있는가에 따라 결정한다.(The certainty of the relationship between the adverse event and the clinical trial intervention (or other causes—such as progression of underlying disease, concomitant treatment, etc.) is determined based on how well the adverse event can be explained from the following perspectives.)
  - 임상시험 중재 방법에 대해 알려진 효과 및 기전(Known effects and mechanisms of the clinical trial intervention)
  - 임상시험용 중재 방법과 유사한 치료법에서 이전에 관찰되었던 유사한 작용(Similar effects previously observed with therapies similar to the investigational intervention)
  - 유사한 중재 방법과 관련 있다고 자주 보고된 반응들(Reactions frequently reported in association with similar interventions)
  - 중재 방법 시술의 시간과 연관되어 나타나는 반응(중지 시 없어지고, 재시술 시 다시 발생)(Reactions that occur in association with the timing of the intervention (disappearance upon discontinuation and recurrence upon re-administration))

1) 관련성이 명백함(Definitely related)
   - 중재 방법과 이상반응 발현의 시간적 순서가 타당한 경우(When the temporal sequence between the intervention and the onset of the adverse event is plausible)
   - 이상반응이 다른 어떤 이유보다 중재 방법에 의해 가장 개연성이 있게 설명되는 경우(When the adverse event is most likely to be explained by the intervention method than by any other cause)
   - 중재 방법 시술 중단으로 이상반응이 사라지는 경우(When the adverse event disappears after discontinuation of the intervention method)
   - 재치료(rechallenge, 가능한 경우에만 실시) 결과가 양성인 경우(When rechallenge (only if feasible) results in a positive outcome)
   - 이상반응이 동일한 중재 방법에 대해 이미 알려져 있는 정보와 일관된 양상을 보이는 경우(When the adverse event shows a pattern consistent with information already known about the same intervention method)

2) 관련성이 많음(Probably related)
   - 중재 방법과 이상반응 발현의 시간적 순서가 타당한 경우(When the temporal sequence between the intervention and the onset of the adverse event is plausible)
   - 이상반응이 다른 원인보다 중재 방법에 의해 더욱 개연성이 있게 설명되는 경우(When the adverse event is more likely to be explained by the intervention method than by any other cause)
   - 중재 방법 시술 중단으로 이상반응이 사라지는 경우(When the adverse event disappears after discontinuation of the intervention method)

3) 관련성이 의심됨(Possibly related)
   - 중재 방법과 이상반응 발현의 시간적 순서가 타당한 경우(When the temporal sequence between the intervention and the onset of the adverse event is plausible)
   - 이상반응이 다른 가능성이 있는 원인들과 같은 수준으로 중재 방법에 기인한다고 판단되는 경우(When the adverse event is judged to be attributable to the intervention method to the same extent as to other possible causes)
   - 중재 방법 시술 중단으로(실시된 경우) 이상반응이 사라지는 경우(When the adverse event disappears after discontinuation of the intervention method (if carried out))

4) 관련성이 적음(Probably not related)
   - 이상반응에 대해 보다 가능성 있는 원인이 있는 경우(When there is a more probable cause for the adverse event)
   - 중재 방법 시술 중단 결과(실시된 경우)가 음성이거나 모호한 경우(When the result of discontinuing the intervention method (if carried out) is negative or ambiguous)
   - 재치료(실시된 경우) 결과가 음성이거나 모호한 경우(When the result of rechallenge (if carried out) is negative or ambiguous)

5) 관련성이 없음(Definitely not related)

- 시험대상자가 중재 방법을 시술받지 않은 경우(When the participant did not receive the intervention method)
- 중재 방법과 이상반응 발현 간의 시간적 순서가 타당하지 않은 경우(When the temporal sequence between the intervention and the onset of the adverse event is not plausible)
- 이상반응에 대해 다른 명백한 원인이 있는 경우(When there is another clear cause for the adverse event)

6) 평가 불가능(Unknown)
- 정보가 불충분하거나 상충되어 판단할 수 없고 이를 보완하거나 확인할 수 없는 경우(When information is insufficient or conflicting, and it cannot be supplemented or confirmed)

15.3 이상반응 보고(Adverse Event Reporting)

15.3.1 이상반응 교육(Adverse Event Education)

- 시험책임자는 시험담당자 및 시험대상자 또는 보호자에게 중재 방법 시술 후 나타날 수 있는 모든 이상반응에 대하여 설명하고 치료 후 나타나는 모든 현상에 대하여 보고하도록 교육을 실시한다.(The principal investigator educates the trial staff and the trial participants or their guardians about all possible adverse events that may occur after the intervention procedure and instructs them to report all phenomena that appear after treatment.)

15.3.2 중대한 이상반응의 보고(Reporting of Serious Adverse Events)

- 시험담당자는 시험 기간 중 발생한 모든 중대한 이상반응을 중재와의 관련성 여부와 상관없이 24시간 이내에 시험책임자에게 보고하도록 한다. 이외에도 시험담당자가 중대하다고 간주하거나, 중재와 연관 지을 수 있는 유의한 위험, 금기, 부작용, 주의사항을 시사하는 사건 등도 중대한 이상반응으로 증례기록지에 기록한다.(The trial staff must report all serious adverse events (SAEs) occurring during the trial period to the principal investigator within 24 hours, regardless of their relationship to the intervention. In addition, any event that the trial staff deems serious or that suggests significant risk, contraindication, adverse effect, or precaution related to the intervention should also be recorded as a serious adverse event in the case report form.)
- 중대한 이상반응이 발생하는 경우에는 시험책임자는 계획서나 임상시험자 자료집 등에서 즉시 보고하지 않아도 된다고 명기한 것을 제외한 모든 중대한 이상반응을 즉시 의뢰자에게 알리고, 발생 5일 이내에 문서로 상세한 내용이 포함된 추가 보고를 실시한다. 별도의 지시가 있을 때까지 일단 임상 시험을 중지한다.(When a serious adverse event occurs, the principal investigator must immediately notify the sponsor of all serious adverse events, except those explicitly stated in the study protocol or investigator's brochure as not requiring immediate reporting. A detailed written report must be submitted within 5 days of occurrence. The clinical trial should be temporarily halted until further notice.)
- 의뢰자는 기타 관련된 시험자, 기관생명윤리위원회에게 중대하고 예상하지 못한 모든 이상 반응을 다음 각 호에서 정한 기간 내에 신속히 보고한다.(The sponsor must promptly report all serious and unexpected adverse events to other relevant investigators and the Institutional

Review Board (IRB) within the following timeframes)

① 사망을 초래하거나 생명을 위협하는 경우에는 의뢰자가 이 사실을 보고 받거나 알게 된 날로부터 7일 이내, 다만, 이 경우 상세한 정보를 최초 보고일로부터 8일 이내로 추가로 보고한다.(For events resulting in death or posing a threat to life: Within 7 days of the sponsor becoming aware of the event. In this case, detailed information must be additionally reported within 8 days of the initial report.)

② 다른 모든 중대하고 예상하지 못한 이상반응의 경우에는 의뢰자가 이 사실을 보고 받거나 알게 된 날로부터 15일 이내에 추가로 보고한다.(For all other serious and unexpected adverse events: Within 15 days of the sponsor becoming aware of the event.)

- 최종 보고 시에는 가능하다면 다음의 정보가 제공되어야 한다: 발생시간, 정도, 처치, 경과, 중재와의 인과관계 등에 대한 정보를 중대한 이상반응 보고서에 기록하여야 한다.(In the final report, the following information should be provided if possible: time of occurrence, severity, management, outcome, and causal relationship with the intervention, all of which should be recorded in the serious adverse event report.)
- 의뢰자는 식품의약품안전처장에게 이상약물반응을 보고할 경우, 이상약물반응 보고서에 임상시험책임자 또는 임상시험담당자로부터 보고 받은 내용을 첨부하여 보고한다. 또한, 해당 이상약물반응이 종결(해당 이상약물반응의 소실 또는 추적조사의 불가 등)될 때까지 추가적인 안전성 정보를 주기적으로 보고한다. 복수의 실시기관에서 임상시험을 실시하는 경우에는 해당 실시기관에 즉시 통보해야 한다. 단, 본 임상시험은 의약품이 중재 방법이 아니기 때문에 해당하지 않는다.(When the sponsor reports an adverse drug reaction to the Ministry of Food and Drug Safety (MFDS), the contents reported by the principal investigator or trial staff must be attached to the adverse drug reaction report. In addition, periodic updates on additional safety information should be reported until the adverse drug reaction is resolved (i.e., the adverse drug reaction disappears or follow-up becomes impossible). If the clinical trial is conducted at multiple sites, the relevant sites must be immediately notified. However, since this clinical trial does not use a drug as the intervention method, this does not apply.)

15.3.3 이상반응 발생 시 조치사항(Actions to Take in Case of Adverse Events)

- 본 시험기간 중 시험책임자, 시험담당자는 시험대상자의 안전에 만전을 기하여야 하며, 예측되지 않은 중대한 이상반응 발생 시에는 신속하고 적절한 조치를 취하여 이상반응을 최소화하여야 한다.(During the trial period, the principal investigator and trial staff must take all possible measures to ensure the safety of the participants. In the event of an unexpected serious adverse event, prompt and appropriate action must be taken to minimize the adverse event.)

15.3.4 이상반응의 추적관찰(Follow-up of Adverse Events)

- 이상반응 발생으로 인하여 조기에 시험을 중지하여야 할 경우에는 이상반응 발생보고서를 작성하고 필요한 경우, 혈액검사 등을 실시한다.(If the trial must be discontinued early due to the occurrence of an adverse event, an adverse event report should be prepared and, if necessary, blood tests or other relevant examinations should be performed.)
- 시험담당자는 이상반응이 나타난 시험대상자에 대해 증상이 소실되고 상태가 안정될 때까지 추

적 관찰해야 하며, 의뢰자가 요청하는 경우 이상반응의 이후 진행 경과에 대한 보고서를 제출하여야 한다.(The trial staff must follow up with the participant who experienced the adverse event until the symptoms have resolved and the participant's condition is stable. If requested by the sponsor, a report on the subsequent course of the adverse event must be submitted.)

16. 시험자의 의무(Investigator's Obligations)

16.1 임상시험기록과 근거문서(Clinical Trial Records and Source Documents)

- 시험자는 모든 임상시험 관련 통신, 시험대상자의 기록, 동의서, 약품의 사용 및 투약기록, 증례기록지 사본을 연구 완료 후 임상시험 실시기관 문서보관실에 3년간 보관한다.(Investigators must retain all clinical trial-related correspondence, participant records, consent forms, records of drug use and administration, and copies of case report forms for 3 years after study completion in the clinical trial site's document archive.)

16.2 임상시험계획서의 수정(Amendment of the Clinical Trial Protocol)

• 시험책임자는 임상시험계획서의 수정이 필요한 경우 기관생명윤리위원회에 이를 알려야 한다.(The principal investigator must notify the Institutional Review Board (IRB) if any amendments to the clinical trial protocol are required.)

16.3 근거자료 공개(Disclosure of Source Data)

- 본 임상시험의 결과로 얻어진 개별 시험대상자의 의학적 정보는 기밀 사항으로 간주되며, 다음에 명시된 경우를 제외하고 제3자에게 공개하는 것을 금한다. 그러한 의학적 정보는 시험대상자 개인의 의사나 시험대상자의 안녕에 책임이 있는 다른 의료인에게 제공될 수 있다. 본 임상시험의 결과로 생성된 근거자료는 모니터링이나 정도 관리, 감사 또는 실태조사를 위해 임상시험수탁기관, 기관생명윤리위원회 또는 식품의약품안전처에 공개될 것이다. 전자증례기록지를 통해 수집된 데이터는 한국한의약진흥원의 접근제어, 이중보안, 데이터베이스의 암호화등의 개인정보보호 정책에 의해 보관되어 활용되며, 최대 10년의 보유 기간이 끝난 후 파기될 것이다.
- Medical information of individual participants obtained as a result of this clinical trial is considered confidential and must not be disclosed to third parties except under the circumstances specified below. Such medical information may be provided to the participant's physician or other healthcare professionals responsible for the participant's well-being. Source data generated as a result of this clinical trial will be disclosed to the clinical trial contract research organization, the Institutional Review Board, or the Ministry of Food and Drug Safety for monitoring, quality control, auditing, or inspection purposes. Data collected through electronic case report forms will be stored and utilized in accordance with the National Development Institute of Korean Medicine's personal information protection

policy, which includes access control, dual security, and database encryption, and will be destroyed after a maximum retention period of 10 years.

17. 시험의 윤리적 수행과 대상자의 안전보호에 관한 대책 (Ethical Conduct of the Trial and Measures for Subject Safety Protection)

17.1 시험의 윤리적 수행(Ethical Conduct of the Trial)

- 본 시험은 ICH GCP 가이드라인, 헬싱키선언(서울 2008), 한국 GCP 가이드라인, 한국 약사법, 기관생명윤리위원회(IRB), 자료 보호에 관한 규정 등 모든 적용 가능한 규정을 준수하여 수행한다.(This trial will be conducted in accordance with all applicable regulations, including the ICH GCP guidelines, the Declaration of Helsinki (Seoul 2008), Korean GCP guidelines, the Korean Pharmaceutical Affairs Act, Institutional Review Board (IRB) requirements, and data protection regulations.)

17.2 시험자의 역할(Role of the Investigator)

- 시험자(Investigator)는 시험책임자, 시험담당자를 말한다.(The investigator refers to the principal investigator and trial staff.)
- 시험 개시 이전에 본 시험계획서(개정판 포함), 서면 환자동의서, 동의 양식의 개정, 환자 등록 절차(예: 광고), 환자에게 제공되는 서면 정보, GCP 요건을 준수하겠다는 서약서에 대하여 IRB의 승인을 서면으로 날짜와 함께 받는다. IRB 승인서에서 계획서의 버전과 검토된 문서들이 확인되도록 한다.(Prior to trial initiation, written approval from the IRB, including the date, must be obtained for this trial protocol (including amendments), written informed consent forms, revisions to consent forms, subject enrollment procedures (e.g., advertisements), written information provided to subjects, and a commitment to comply with GCP requirements. The IRB approval document must confirm the protocol version and the documents reviewed.)
- 시험자는 계획서를 준수하여 임상시험을 실시하여야 한다. 임상시험 중 또는 임상시험 이후에도, 시험자는 임상적으로 의미 있는 모든 이상반응에 대해 시험대상자가 적절한 의학적 처치를 받을 수 있도록 조치하여야 하고, 시험자가 알게 된 시험대상자의 병발 질환에 대해 의학적 처치가 필요한 경우 이를 시험대상자에게 알려주어야 한다. 시험자는 임상시험계획을 정확히 분석 및 숙지하고 대상 시험대상자의 문제점에 적극적으로 대응한다.(The investigator must conduct the clinical trial in accordance with the protocol. During or after the clinical trial, the investigator must ensure that subjects receive appropriate medical management for all clinically significant adverse events and inform subjects if medical management is required for any concurrent illnesses the investigator becomes aware of. The investigator must accurately analyze and understand the clinical trial plan and proactively address any issues related to the subjects.)

17.3 임상시험 실시기관의 역할(Role of the Clinical Trial Site)

- 실시기관의 장은 각 임상시험 단계별로 해당 임상시험의 실시에 필요한 임상시험실 및 설비와 전문인력을 갖추고 해당 임상시험을 적정하게 실시할 수 있도록 준비를 철저히 해야 한다.(The head of the site must thoroughly prepare at each stage of the clinical trial by ensuring that the necessary clinical trial facilities, equipment, and professional personnel are available to properly conduct the trial.)

17.4 임상시험 계획서의 승인 및 수정(Approval and Modification of the Clinical Trial Protocol)

- 임상시험계획서는 기관생명윤리위원회에 보고하고 승인받아야 한다. 승인 이전에 시험대상자를 임상시험에 참여시킬 수 없다.(The clinical trial protocol must be reported to and approved by the IRB. Subjects cannot participate in the clinical trial before approval.)
- 임상시험계획서를 시험절차가 더 광범위해지거나 위험도가 높아지거나 시험대상자 선정기준에 변화가 있거나 추가적인 안전성 정보로 인해 임상시험계획서를 변경하는 경우에는 수정을 해야 한다. 임상시험계획서를 수정할 때에는 개정 일자, 개정 이유, 개정 내용 등을 기록하여 보관하여야 하며 기관생명윤리위원회에 보고해야 한다.(The protocol must be revised if the trial procedures become more extensive, the risk increases, the subject selection criteria change, or additional safety information is obtained. When modifying the protocol, the date, reason, and content of the revision must be recorded and retained, and the IRB must be notified.)
- 시험자는 시험대상자에게 발생한 즉각적 위험 요소의 제거가 필요한 경우를 제외하고는, 계획서와 다르게 임상시험을 실시하여서는 안 된다.(Except in cases where immediate risk to subjects must be removed, the investigator must not conduct the clinical trial in a manner different from the protocol.)

17.5 임상시험 계획서의 숙지(Familiarization with the Clinical Trial Protocol)

- 시험책임자 및 담당자들은 시험계획을 정확히 분석 및 숙지하고 임상시험 대상 시험대상자의 문제점을 적극적으로 대응한다.(The principal investigator and trial staff must accurately analyze and understand the trial plan and proactively address any issues related to the subjects.)

17.6 시험대상자 동의(Subject Consent)

- 시험대상자 동의(Patient Informed Consent)는 시험대상자가 임상시험 참여 여부를 결정하기 전(시험과 관련된 임의의 모든 절차를 시작하기 전)에 서면을 통하여 이루어진다.(Subject consent (Patient Informed Consent) must be obtained in writing before the subject decides to participate in the trial (before any trial-related procedures begin.)
- 의뢰자는 동의서 요건들을 충족하면서 시험대상자가 쉽게 이해할 수 있게 기술된 동의서 견본을 제공하여 기관생명윤리위원회의 승인을 얻는다.(The sponsor will provide a consent form template that meets the requirements and is written in language easily understood by subjects, for IRB approval.)

- 시험대상자 본인 또는 대리인이 동의서 서식, 시험대상자 설명서 및 기타 문서화된 정보를 읽을 수 없는 경우에는 공정한 입회자가 동의를 얻는 전 과정에 참석하여야 한다.(If the subject or their legal representative is unable to read the consent form, subject information sheet, or other documented information, an impartial witness must be present throughout the entire consent process.)
- 시험자는 시험대상자를 위한 설명서를 통해 본 시험에 참여함에 따른 이익과 위험을 포함하여 임상시험과 관련된 모든 정보를 제공하고, 서명과 서명 날짜가 포함된 문서에 서명하도록 하여 본인이 자발적으로 임상시험에 참여함을 확인하여야 한다.(The investigator must provide all information related to the clinical trial, including the benefits and risks of participation, through the subject information sheet, and require the subject to sign a document with their signature and date to confirm voluntary participation.)
- 시험자는 시험대상자 또는 시험대상자의 법정 대리인이 직접 서명하고 날짜를 쓴 동의서 원본을 보관하고 사본을 제공하여야 한다.(The investigator must retain the original consent form signed and dated by the subject or their legal representative and provide a copy to the subject.)
- 동의를 얻기 전에 시험자는 시험대상자 또는 대리인이 임상시험의 세부 사항에 대해 질문하고 해당 임상시험의 참여 여부를 결정할 수 있도록 충분한 시간과 기회를 주어야 하며, 모든 임상시험 관련 질문에 대해 시험대상자 또는 대리인이 만족할 수 있도록 대답해 주어야 한다.(Before obtaining consent, the investigator must give the subject or their representative sufficient time and opportunity to ask questions and decide on participation, and must answer all clinical trial-related questions to the satisfaction of the subject or their representative.)
- 동의서 양식은 시험 기간 중 시험대상자의 안전과 관련된 중요한 정보가 입수되는 경우 개정될 수 있다. 이 경우 추가 정보에 관하여 항상 IRB와 기존 시험대상자에게 알리고 동의를 다시 얻어야 하며 이는 이전과 같은 방법으로 기록된다.(The consent form may be revised if important information related to subject safety is obtained during the trial. In this case, the additional information must always be communicated to the IRB and existing subjects, and consent must be obtained again, recorded in the same manner as before.)
- 시험자는 시험대상자의 동의하에 시험대상자의 주치의에게 시험대상자의 임상시험 참가를 알린다.(With the subject's consent, the investigator will inform the subject's primary physician of the subject's participation in the trial.)

17.6.1 인지능력이 손상된 취약한 시험대상자를 포함하는 경우 보호조치 방안 (Protection Measures for Vulnerable Subjects with Impaired Cognitive Capacity)

- 시험대상자의 동의 능력을 평가하게 되며 동의 능력에 대한 기준은 다음과 같다.(**The subject's capacity to consent will be assessed, and the criteria for consent capacity are as follows.)**

  ① 선택했다는 사실을 증명할 수 있는 능력을 확인: '예' 또는 '아니오'라고 의사소통한다. (Ability to demonstrate choice: The subject can communicate 'yes' or 'no.')

  ② 관련 정보를 이해할 수 있는 능력: 시험대상자가 연구 절차, 동의과정에서 제공된 정보를

눈높이에 맞추어 쉬운 단어와 설명으로 이해할 수 있게 한다.(Ability to understand relevant information: The subject can understand the research procedures and information provided during the consent process using simple words and explanations tailored to their level of understanding.)

③ 대리인의 동의는 최소 위험 연구이기 때문에 대리인의 결정은 해당 시험대상자가 판단 능력이 있었을 때 가지고 있었을 관점을 반영하도록 하며 시험대상자의 생각을 알 수 없을 시, 대상자에게 최선의 이익이 될지에 따라 대리인의 결정이 이루어지도록 한다.(Proxy consent: For minimal risk research, the proxy's decision should reflect the perspective the subject would have had if capable, and if the subject's wishes are unknown, the proxy's decision should be made in the subject's best interest.)

- 대상자의 인지저하 및 판단 능력을 알 수 없다고 판단될 때, 임상시험 동의 취득 시 대상자 본인과 보호자 또는 대리인, 참관인의 동의를 함께 받을 것이다.(If the subject's cognitive decline or decision-making capacity is unclear, consent will be obtained from the subject, their guardian or proxy, and an observer together.)
- 인지장애 질환의 특성상 참여시에 자발적 동의를 얻었더라도 증상이 악화되어 자발적 참가 동의 유지 여부를 확인하기 어려운 경우가 발생할 수 있으며, 이 경우 대리인의 도움을 요청할 수 있다.(Due to the nature of cognitive disorders, even if voluntary consent is obtained at the time of participation, symptoms may worsen, making it difficult to confirm continued voluntary consent. In such cases, assistance from a proxy may be requested.)

17.7 정확한 시험대상자의 선정(Accurate Subject Selection)

- 본 임상시험에 앞서 시험대상자와의 충분한 면담 및 검사를 통하여 시험대상자 적합 여부에 대하여 철저히 평가하여야 한다.(Prior to this clinical trial, thorough evaluation of subject eligibility must be conducted through sufficient interviews and examinations with the subject.)

17.8 임상시험의 진행 점검(Monitoring of Trial Progress)

- 임상시험책임자는 의뢰자에게 주기적으로 이상반응, 시험진행, 상황, 결과 등에 대하여 보고하며 임상시험 의뢰자는 주기적으로 임상시험 진행 상황에 대하여 점검을 시행한다.(The principal investigator will periodically report adverse events, trial progress, status, and results to the sponsor, and the sponsor will periodically review the progress of the clinical trial.)

17.9 모니터링의 역할(Role of Monitoring)

- 모니터링(Monitoring)은 임상시험 진행 과정을 감독하고, 해당 임상시험이 계획서, 표준작업지침서, 임상시험 관리기준 및 관련 규정에 따라 실시, 기록되는지를 정기적으로 검토, 확인한다.(Monitoring supervises the progress of the clinical trial and regularly reviews and confirms that the trial is conducted, recorded, and documented in accordance with the protocol, standard operating procedures, GCP, and relevant regulations.
- 임상시험에 대한 모니터링은 모니터링 담당자의 정기적인 시험자 방문과 전화로 이루어지도록 한다. 방문 시 모니터는 시험대상자 기록 원본, 자료보관(연구 파일) 등을 확인한다. 또한, 모니

터는 임상시험 진행 과정을 잘 살피고, 문제가 있으면 시험자와 상의한다.(Monitoring for the clinical trial is conducted through regular site visits and telephone calls by the monitor. During visits, the monitor reviews original subject records and study files. The monitor also closely observes the progress of the clinical trial and consults with the investigator if any issues arise.)

- 본 임상시험에서는 임상시험책임자가 연구 개시 이후 최초 1명 모집 후, 이후 15명 단위로 모집이 완료되었을 때마다 모니터링을 시행하기로 한다.(In this clinical trial, monitoring will be conducted after the recruitment of the first subject and then after every 15 subjects are recruited.)

17.10 시험대상자 기록의 비밀보장(Confidentiality of Subject Records)

- 시험대상자의 신원을 파악할 수 있는 기록은 비밀로 보장될 것이며, 임상시험의 결과가 출판될 경우에도 시험대상자의 신원을 비밀상태로 유지한다.(Records that can identify subjects will be kept confidential, and subject identities will remain confidential even if trial results are published.)
- 본 시험에 관련된 의뢰자, 모니터 및 점검자는 본 시험의 모니터링과 점검 및 진행 사항 관리를 위한 목적으로 시험대상자의 기록을 열람할 수 있다. 시험자는 본 계획서에 서명함으로써, 국내의 법규와 윤리적 측면에서 임상시험 의뢰자 또는 임상시험수탁기관의 모니터 및 점검자가 시험대상자의 차트와 증례기록지 기록을 검증하기 위하여 해당 문서를 검토하거나 복사할 수도 있음을 인정한다. 이러한 정보들은 기밀로 보관되어야 하며, 기밀 보관을 위한 시설과 그 관리 기준을 갖추고 있어야 한다. 연구 자료는 잠금 장치에 보관하며, 접근이 제한된 컴퓨터에 저장한다.(The sponsor, monitor, and auditor may review subject records for the purpose of monitoring, auditing, and managing trial progress. By signing this protocol, the investigator acknowledges that, in accordance with domestic laws and ethical considerations, the sponsor or contract research organization's monitor and auditor may review or copy subject charts and case report forms. This information must be kept confidential, and facilities and management standards for confidentiality must be in place. Research data will be stored in locked cabinets and on computers with restricted access.)
- 한편, 증례기록지 등 임상시험에 관련된 모든 서류에는 시험대상자 이름이 아닌 시험대상자 식별코드(일반적으로 시험대상자 이니셜)로 기록하고 구분한다. 증례기록지 등 임상시험에 관련된 서류는 연구 종료 후 3년 이후 폐기한다.(All clinical trial documents, such as case report forms, will be identified and distinguished by subject identification codes (usually subject initials), not by names. Clinical trial documents such as case report forms will be destroyed three years after the end of the study.)

17.11 이상반응 발생 시 조치(Actions in Case of Adverse Events)

- 이상반응 발생 시 즉시 담당의사로부터 필요 검사 및 치료를 받을 수 있도록 관리한다. 중대한 이상반응 발생 시에는 시험을 중지하고 15.3.3항의 이상반응 발생 시 조치사항에 따라 신속하고

적절한 조치를 취한다.(If an adverse event occurs, subjects will be promptly managed to receive necessary tests and treatment from the responsible physician. In case of a serious adverse event, the trial will be stopped, and prompt and appropriate actions will be taken in accordance with section 15.3.3 (Actions in Case of Adverse Events).)

17.12 자료의 보관(Data Retention)

• 임상시험 실시와 관련된 각종 자료 및 기록을 잘 보존하도록 보관하는 장소가 따로 준비되어 있고 보안을 유지하도록 한다. 결과보고서 작성 이후에는 임상시험 관련 문서를 임상시험 실시기관 문서보관실에 임상시험 완료일로부터 3년간 보관한다.(A separate, secure location will be prepared to preserve and safeguard all trial-related data and records. After the final report is prepared, all clinical trial documents will be stored in the clinical trial site’s document archive for three years from the date of trial completion.)

18. 임상시험 담당자(Clinical Trial Staff)

[별첨1] 임상시험 담당자

[Appendix 1] Clinical Trial Staff

19. 피해자 보상에 대한 규약(Compensation for Damage)

[별첨2] 피해자 보상에 대한 규약

[Appendix 2] Compensation for Damage

20. 시험 대상자 설명문 및 동의서(Subject Information Sheet and Consent Form)

[별첨3] 시험 대상자 설명문 및 동의서

[Appendix 3] Subject Information Sheet and Consent Form

21. 증례기록지(Case Report Form)

[별첨4] 증례기록지

[Appendix 4] Case Report Form

22. 모집 광고문(Recruitment Advertisement)

[별첨5] 모집 광고문

[Appendix 5] Recruitment Advertisement

23. 참고문헌 (References)

1) Awan, K. H., & Patil, S. (2015). The role of transcutaneous electrical nerve stimulation in the management of temporomandibular joint disorder. J Contemp Dent Pract, 16(12), 984-6.
2) Andre A, Kang J, Dym H. Pharmacologic treatment for temporomandibular and temporomandibular joint disorders. Oral and Maxillofacial Surgery Clinics. 2022;34(1):49-59.
3) Anupriya, C., Nahar, P., Singh, M. P., Bhuvaneshwari, S., Goel, S., & Mathur, H. (2023). TENS therapy or low-level laser therapy? In the management of morbidities associated with temporomandibular joint disorders: A comparative study. Journal of Indian Academy of Oral Medicine and Radiology, 35(2), 187-190.
4) Chen L, Zhang S, Tan Y, Zheng Y, Fang S, Yi Y, Xiong X. Anxiety mediates association between sex and jaw function limitation in temporomandibular disorder patients from China. Frontiers in Neurology. 2024;15:1398788.
5) Chellappa, D., & Thirupathy, M. (2020). Comparative efficacy of low-Level laser and TENS in the symptomatic relief of temporomandibular joint disorders: A randomized clinical trial. Indian Journal of Dental Research, 31(1), 42-47.
6) Kirupa, K., Divya Mary, S., Vaishnavi, G., Nisha, R. N., Mercy, J. R., & Jaiganesh, G. (2019). A comparative study of ultrasound therapy and transcutaneous electrical nerve stimulation in reducing pain for temporomandibular joint disorder. Drug Invent Today, 12(3), 515-7.
7) Kmeid E, Nacouzi M, Hallit S, Rohayem Z. Prevalence of temporomandibular joint disorder in the Lebanese population, and its association with depression, anxiety, and stress. Head & face medicine. 2020 Dec;16:19.
8) Mishra, S., Bajoria, A. A., Sangamesh, N. C., Swain, A. K., Sahoo, S. K., & Mohapatra, A. (2024). Low-Level Laser and TENS Therapy Assessment for the Treatment of Temporomandibular Joint Disorder. Journal of Pharmacy and Bioallied Sciences, 16(Suppl 3), S2179-S2181.
9) Murphy MK, MacBarb RF, Wong ME, Athanasiou KA. Temporomandibular disorders: a review of etiology, clinical management, and tissue engineering strategies. The International Journal of Oral & Maxillofacial Implants. 2013 Nov-Dec;28(6):e393-414.
10) Ohrbach R, Gonzalez Y, List T, Michelotti A, Schiffman E. Diagnostic criteria for temporomandibular disorders (DC/TMD) clinical examination protocol.: Version 02June2013. Available online at: www. rdc-tmdinternational. org (accessed Oct 14, 2024).
11) Ohrbach R, editor. Diagnostic Criteria for Temporomandibular Disorders: Assessment Instruments. Version 15May2016. [측두하악장애 진단기준 (DC/TMD) 평가도구: Korean Version 27Oct2019] JW Chung, JW Park,Trans. www.rdc-tmdinternational.org Accessed on <date>.
12) Rezaie K, Amiri A, Ebrahimi Takamjani E, Shirani G, Salehi S, Alizadeh L. The Efficacy of Neck and Temporomandibular Joint (TMJ) Manual Therapy in Comparison With a Multimodal Approach in the Patients with TMJ Dysfunction: A Blinded Randomized Controlled Trial. Med J Islam Repub Iran. 2022;36:45.
13) Valesan LF, Da-Cas CD, Réus JC, Denardin AC, Garanhani RR, Bonotto D, Januzzi E, de Souza BD. Prevalence of temporomandibular joint disorders: a systematic review and meta-analysis. Clinical Oral Investigations. 2021;25:441-53.
14) 이영준 지음. 턱관절 균형요법. 파주: 물고기숲, 2019.
15) 한의표준임상진료지침개발사업단. (2021). 턱관절 장애 한의표준임상진료지침.
